# Effects of isoflavones on breast tissue and the thyroid hormone system in humans: a comprehensive safety evaluation

**DOI:** 10.1007/s00204-018-2279-8

**Published:** 2018-08-21

**Authors:** S. Hüser, S. Guth, H. G. Joost, S. T. Soukup, J. Köhrle, L. Kreienbrock, P. Diel, D. W. Lachenmeier, G. Eisenbrand, G. Vollmer, U. Nöthlings, D. Marko, A. Mally, T. Grune, L. Lehmann, P. Steinberg, S. E. Kulling

**Affiliations:** 1 Institute for Food Toxicology, Senate Commission on Food Safety, University of Veterinary Medicine Hannover, Hannover, Germany; 2 Department of Experimental Diabetology, German Institute of Human Nutrition (DIfE), Nuthetal, Germany; 3 Department of Safety and Quality of Fruit and Vegetables, Max Rubner-Institut, Federal Research Institute of Nutrition and Food, Haid-und-Neu-Str. 9, 76131 Karlsruhe, Germany; 4 Institut für Experimentelle Endokrinologie, Charité-Universitätsmedizin Berlin, Corporate Member of Freie Universität Berlin, Humboldt-Universität zu Berlin, Berlin Institute of Health, CVK, Berlin, Germany; 5 Department of Biometry, Epidemiology and Information Processing, University of Veterinary Medicine Hannover, Hannover, Germany; 6 Department of Molecular and Cellular Sports Medicine, Institute of Cardiovascular Research and Sports Medicine, German Sport University Cologne, Cologne, Germany; 7 Chemisches und Veterinäruntersuchungsamt Karlsruhe, Karlsruhe, Germany; 8 Division of Food Chemistry and Toxicology, Molecular Nutrition, Department of Chemistry, Technische Universität Kaiserslautern, Kaiserslautern, Germany; 9 Department of Biology, Molecular Cell Physiology and Endocrinology, Technische Universität Dresden, Dresden, Germany; 10 Department of Nutrition and Food Sciences, Nutritional Epidemiology, Rheinische Friedrich-Wilhelms University Bonn, Bonn, Germany; 11 Department of Food Chemistry and Toxicology, Faculty of Chemistry, University of Vienna, Vienna, Austria; 12 Department of Toxicology, University of Würzburg, Würzburg, Germany; 13 Department of Molecular Toxicology, German Institute of Human Nutrition (DIfE), Nuthetal, Germany; 14 Department of Food Chemistry, Institute for Pharmacy and Food Chemistry, University of Würzburg, Würzburg, Germany; 15 Institute for Food Toxicology, University of Veterinary Medicine Hannover, Hannover, Germany; 16 Present Address: Max Rubner-Institut, Federal Research Institute of Nutrition and Food, Haid-und-Neu-Str. 9, 76131 Karlsruhe, Germany

**Keywords:** Isoflavones, Human intervention studies, Observational studies, Breast tissue, Thyroid hormone system, Safety evaluation

## Abstract

Isoflavones are secondary plant constituents of certain foods and feeds such as soy, linseeds, and red clover. Furthermore, isoflavone-containing preparations are marketed as food supplements and so-called dietary food for special medical purposes to alleviate health complaints of peri- and postmenopausal women. Based on the bioactivity of isoflavones, especially their hormonal properties, there is an ongoing discussion regarding their potential adverse effects on human health. This review evaluates and summarises the evidence from interventional and observational studies addressing potential unintended effects of isoflavones on the female breast in healthy women as well as in breast cancer patients and on the thyroid hormone system. In addition, evidence from animal and in vitro studies considered relevant in this context was taken into account along with their strengths and limitations. Key factors influencing the biological effects of isoflavones, e.g., bioavailability, plasma and tissue concentrations, metabolism, temporality (pre- vs. postmenopausal women), and duration of isoflavone exposure, were also addressed. Final conclusions on the safety of isoflavones are guided by the aim of precautionary consumer protection.

**Electronic supplementary material:**

The online version of this article (10.1007/s00204-018-2279-8) contains supplementary material, which is available to authorized users.

## Introduction

Isoflavones are secondary plant constituents mainly occurring in soy and red clover. Soy bean, soy germ, and red clover extracts or other isoflavone-containing preparations are marketed, e.g., as food supplements as well as ‘dietary foods for special medical purposes’ and other commodities and are available over the counter in pharmacies, health food shops, supermarkets, and on the internet. According to the European Food Safety Authority (EFSA) (EFSA 2015) and the Federal Institute for Risk Assessment (BfR) (BfR 2015), dosages of soy isoflavones of up to 100 mg per day for a duration of up to 10 months were suggested to be acceptable in products intended for healthy postmenopausal women. Based on the fact that some isoflavones may exhibit an estrogenic activity, these products were often advertised as ‘natural compounds being able to mitigate menopausal symptoms’. However, EFSA concluded that the scientific evidence was insufficient to substantiate this claimed effect (EFSA 2011a, 2012). The same holds true for all the other isoflavone-related claims submitted to EFSA, e.g., regarding their association with bone health, cardiovascular health, prostate function, female hormonal balance, as well as antioxidant effects (EFSA 2009, 2010, 2011a, b, c, 2012). For this reason, advertisement of health effects regarding isoflavones in food supplements and other foods is no longer authorized in the European Union.

The Permanent Senate Commission on Food Safety of the German Research Foundation (SKLM) evaluated the safety of isoflavones in food supplements and in supplemental balanced diets in 2006 and 2009 [adopted on 10th November 2006 (SKLM 2007; English version adopted on 23rd May 2007), and updated on 20th February 2009]. Data on the isoflavone content in food and food supplements as well as the dietary intake of isoflavones, their bioavailability, metabolism, and biological effects were taken into account. These previous opinions focused on potential adverse effects of isoflavones on the breast and the thyroid gland considered as the most relevant target organs in postmenopausal women. This consumer group is expected to be primarily exposed to isoflavone preparations.

Guided by the principle of precautionary consumer protection, the SKLM expressed the concern that specifically in an isolated form isoflavones might negatively influence already existing breast tumour cells, may enhance the risk to develop a subclinical hypothyroidism, or may contribute to the development of goitre under specific circumstances such as iodine deficiency. This evaluation was mainly based on results from cell culture and animal studies. Because of the very limited results from human studies at that time, the available data did not allow a conclusive assessment of the effects of isoflavones in humans.

Therefore, the SKLM decided to renew their evaluation of the effects of isoflavones on the female breast and thyroid gland in humans. The focus was placed on the available human studies in both pre- as well as postmenopausal women, considering predominantly isoflavone exposure derived from soy-based supplements and soy food, including soy protein preparations. Results from recent animal and in vitro studies, which were of particular relevance to understand the mechanism(s) or mode(s) of action of isoflavones in the breast and thyroid gland are also discussed.

## Isoflavone classification

**Fig. 1:**
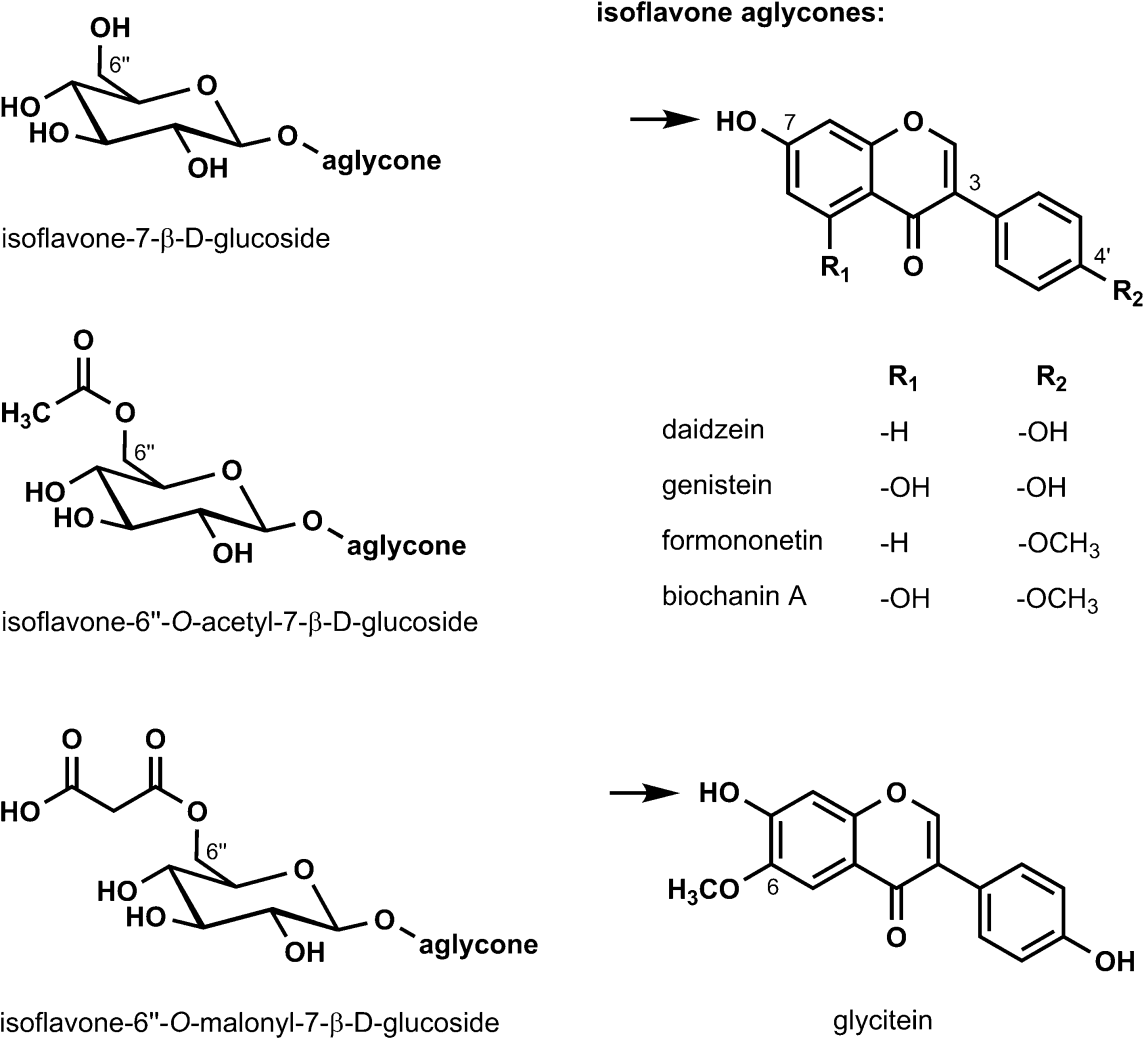
Chemical structures of isoflavones in soy and red clover and their glucose conjugates (left row). The 7-*O*-ß-d-glucosides of daidzein, genistein, and glycitein are named daidzin, genistin, and glycitin. The arrows in the figure indicate the position of glucose conjugation (SKLM 2009)

Soybeans and soy-based food supplements contain the isoflavones genistein, daidzein, and glycitein (aglycones) (Fig. 1), which are mainly present as glucosides (e.g., genistin, daidzin, and glycitin) or esterified glucosides and only in minor amounts in their free form as aglycones (Andres et al. 2015; Delmonte and Rader 2006). The major constituents of red clover and red clover-based food supplements are formononetin and biochanin A (Spagnuolo et al. 2014) (Fig. 1), which are essentially present in the aglycone form (Maul et al. 2005; Setchell et al. 2001), but other isoflavones, such as genistein, daidzein, glycitein, prunetin, and irilone, were also identified (Andres et al. 2015; Tsao et al. 2006; Wu et al. 2003). Because soy and red clover extracts are not produced according to standardised production processes, the preparations might differ in quantity and quality with respect to their associated matrix (e.g., further bioactive components, such as lysophosphatides and saponines) (Fang et al. 2004). Independently of the raw material and the extraction process, the preparations may also vary regarding the formulations, including added ingredients, e.g., vitamins, minerals, or other plant extracts (SKLM 2007, 2009).

## Isoflavone exposure

The highest levels of isoflavones are present in soy and soy-based food, but lower amounts were also found in other foods of plant and animal origin (EFSA 2015). In particular, soy-based products, such as soy milk, tofu, or soy yoghurt, might contain high levels of isoflavones (e.g., approximately 10–84 mg isoflavones/100 g). However, the isoflavone content reported in the literature varies considerably within the same food group, which probably reflects the fact that the spectrum of analysed isoflavones differs between studies. Levels of isoflavones in food were already summarised in detail in the opinions of SKLM (SKLM 2009) and EFSA (EFSA 2015).

Whether isoflavones are predominantly present as glucosides or as aglycones depends on the type of soy food and food processing. In non-fermented soy products, such as soy milk (also referred to as soy drink or soybean juice) and most of the tofu sold in Western countries, isoflavone glucosides predominate, whereas aglycones occur primarily in fermented soy products. In Asian countries, mainly fermented soy products, such as tempeh, miso, or natto, are part of the traditional diet. This leads to a daily isoflavone intake of about 16–70 mg, mainly as aglycones (Frankenfeld et al. 2004; Hirose et al. 2005; Hu et al. 2014; Iwasaki et al. 2009; Lee et al. 2007). It is assumed that in these geographical areas, the consumption of soy foods normally starts early in life and consumers are exposed to isoflavones throughout their entire lifetime. In contrast, soy products are not foods traditionally consumed in Western countries, whereby the average daily intake is estimated to be less than 1–2 mg isoflavones/day (EFSA 2015; SKLM 2009) with the exception of the UK, where consumption is estimated to be up to 3 mg/day (EFSA 2015). Vegetarians and consumers preferring a soy-rich diet within Western populations on average have higher intake estimates of up to 20 mg/day (EFSA 2015). Since isoflavone-containing foods are not part of the traditional Western diet, it can be assumed that consumption generally starts later in life and is often limited to special occasions or for rather short periods of time. Moreover, even in Asia, nutritional habits as well as food preparation (cooking) and processing techniques of soy beans and products thereof were different in the past, which may also have led to lower intakes than today. For example, traditional soy food preparation was based on soaking, rinsing, and prolonged simmering in water, all of which lead to a depletion of glycosylated isoflavones in the product (Fernandez-Lopez et al. 2016).

The daily dose of isoflavones from food supplements, as recommended by the manufacturers, ranges from 20 to 150 mg/day for soy-based supplements and from 40 to 80 mg/day for red clover-based supplements (EFSA 2015). However, the daily intake of isoflavones from food supplements was recently estimated based on the measured content of isoflavones in various food supplements available on the market and the recommended number of servings (EFSA 2015). It was reported to be extremely variable and ranged from approximately 0.1 to 100 mg/day for soy isoflavones and from 30 to 160 mg/day for isoflavones from red clover (expressed as aglycone equivalents). Overall, there are still uncertainties with regard to the actual content and the relative intake of isoflavones from food supplements (EFSA 2015). Furthermore, the intake, expressed on a body weight basis, may vary considerably.

These data indicate that the isoflavone intake from the diet by European women is considerably below the minimal daily intake of soy isoflavones from food supplements recommended by the manufacturers (20–35 mg/day). However, it was noted that the intake of isoflavones by people preferring the consumption of certain soy-based food products might be within the same order of magnitude estimated for users of food supplements (EFSA 2015). Intake estimates from the diet of the Asian population are within the same range as daily doses for food supplements recommended by the manufacturers.

EFSA (2015) noted that the presence of soy in several processed foods may lead to an additional intake of isoflavones. Because of a lack of data, this additional intake was not included in the latest available exposure estimations, which may lead to an underestimation of dietary isoflavone exposure (EFSA 2015).

## Isoflavone metabolism and concentrations in plasma and target tissues

### Metabolism

Various isoflavones are metabolised in different ways. The biotransformation of daidzein and genistein is well documented (Mortensen et al. 2009; Rafii 2015; Soukup et al. 2016a, b; Yang et al. 2012). It is noteworthy that substantial differences in the phase II and microbial metabolism were reported for these two soy isoflavones (Soukup et al. 2016a, b).

**Fig. 2:**
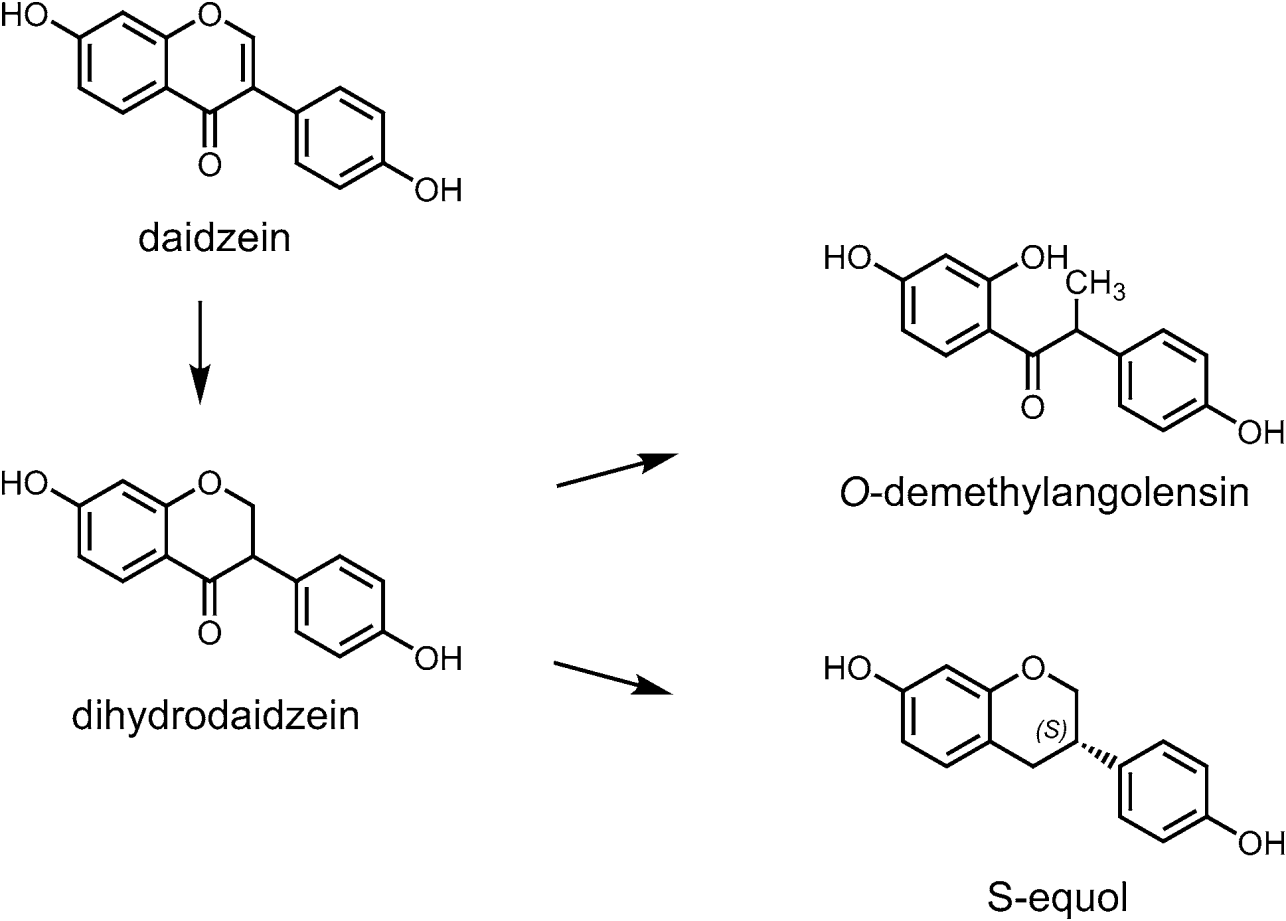
Metabolism of daidzein by intestinal microbiota (SKLM 2009)

**Fig. 3:**
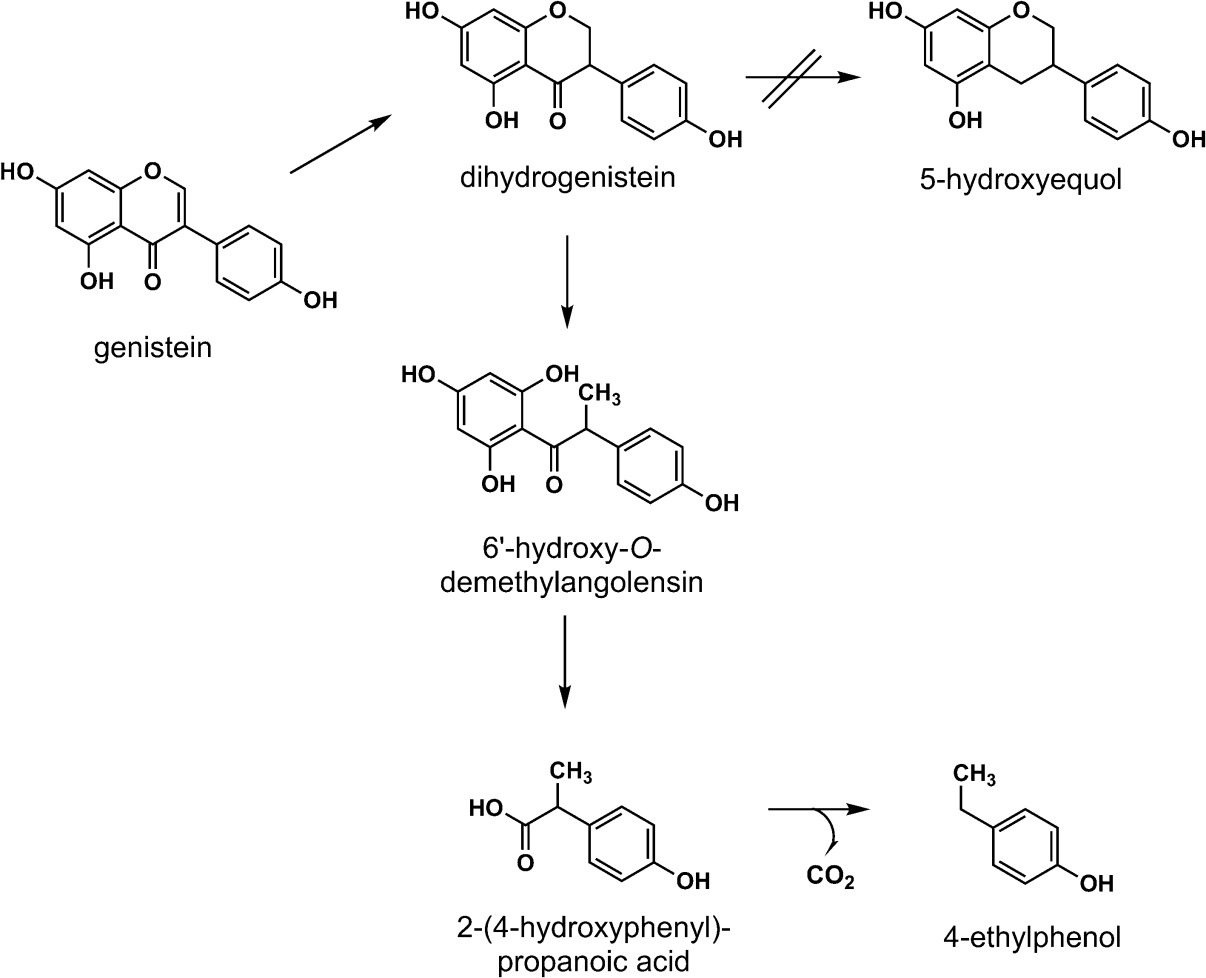
Metabolism of genistein by intestinal microbiota (SKLM 2009)

If isoflavones are ingested as glucosides, the β-*O*-glucosidic bonds can be cleaved by β-glucosidases in the small intestine or by the gut microbiota, which leads to the release of the respective aglycones. Following their absorption into the enterocytes, the aglycones are mainly metabolised in the intestinal tract as well as in the liver by endogenous phase I and phase II metabolising enzymes. The phase I metabolism is proposed to be of minor importance, since the extent of the cytochrome P450-catalysed hydroxylation to the catechol metabolites 6-, 8-, and 3′-hydroxy-daidzein as well as 6-, 8-, and 3′-hydroxy-genistein is found to be low (Kulling et al. 2002; Rüfer et al. 2005). In contrast, the phase II metabolism resulting in sulfonated and glucuronidated conjugates as well as transformation reactions mediated by the intestinal microbiota plays an important role. In general, daidzein can be reduced by gut microbiota to dihydrodaidzein, and further converted to *O*-demethylangolensin (*O*-DMA) and/or *S*(−)-equol (from now on referred to as equol); genistein can be metabolised to dihydrogenistein and then to 6′-hydroxy-*O*-DMA (6′-OH-*O*-DMA), which in turn can be degraded to 4-ethylphenol (Figs. 2, 3). The metabolism of dihydrogenistein to 5-hydroxyequol has not been observed in humans until now. While the formation of equol is well documented, little is known so far regarding the relevance of the degradation reaction of genistein to 4-ethylphenol in humans.

A comprehensive comparison of the phase I/II and microbial metabolism of daidzein and genistein in adult humans, rats, and mice revealed considerable differences between the investigated species (Hosoda et al. 2011; Soukup et al. 2014, 2016a, b). Considering the phase II metabolism, the 4′-sulfo-7-glucuronides (39–49%) and in case of genistein also the diglucuronide (34% of the measured genistein and genistein phase II metabolites) were reported to be the major metabolites in human plasma (Hosoda et al. 2011; Soukup et al. 2014, 2016a, b). In mice, monoglucuronides (30–40%) and monosulfates (33–41%) of daidzein and genistein predominated. In addition, sex-specific differences were observed in rats (Soukup et al. 2016a, b). In male rats, the major conjugates were the disulfates (23–62%) and 4′-sulfo-7-glucuronides (19–54%), whereas in females, mainly, 7-glucuronides were observed. However, it should be taken into account that the proportion of sulfonated vs. glucuronidated metabolites might be influenced by the isoflavone dose and a possible saturation of the sulfonation process (Koster et al. 1981).

The aglycones are of particular interest, since they are regarded as the biologically most active form (see “Mechanistic background”). The amount of aglycones in plasma was low in humans (0.5–1.3%) and rats (0.5–3.1%), but comparatively high in mice (3.1–26.0%), particularly for daidzein (Soukup et al. 2016a, b). These findings point to the difficulties in transferring the results obtained in animal experiments to the human situation. Furthermore, it has been reported that phase II conjugation does not automatically lead to biologically inactive metabolites in each case, since e.g., the daidzein-7-sulfate possesses a higher estrogenic activity if compared to daidzein itself (Pugazhendhi et al. 2008; see “Mechanistic background”). Notably, certain isoflavone conjugates might represent an intracellular reservoir releasing aglycones into target cells as reported for resveratrol-sulfates (Patel et al. 2013).

Since the gut microbiota plays an important role in the metabolism of isoflavones, factors influencing the composition of the gut microbiota may have a substantial impact on plasma concentrations of isoflavones and their metabolites. These factors comprise, e.g., the use of antibiotics and other drugs, bowel diseases as well as the diet, which may influence gut motility, the intestinal transit time, the redox potential, and pH of the intestine and the bile secretion (Mortensen et al. 2009). Further host factors that may influence biotransformation of isoflavones in the gut are age, sex, genetics, and ethnicity (Mortensen et al. 2009). In humans, marked inter-individual variations in the microbial metabolism were observed, particularly with respect to the formation of equol, but also regarding the microbial degradation of genistein to 4-ethylphenol. Notably, equol is proposed to be a biologically active key metabolite of daidzein, whereas 4-ethylphenol lacks estrogenic activity (see “Mechanistic background”; Lampe et al. 1998; Setchell et al. 1984, 2002). Equol is formed in the intestinal tract of only about one-third of the human population, referred to as equol producers. Humans who are not able to form equol in an appreciable concentration, the so-called non-equol producers, metabolise in part daidzein to dihydrodaidzein and *O*-DMA (Fig. 2). In contrast, rats and mice were reported to be 100% equol producers (Soukup et al. 2016a, b). The highly variable formation of microbial metabolites in humans might lead to substantial differences with respect to biological effects, in particular estrogenic and anti-thyroidal activities as reported in in vitro studies.

### Concentrations in plasma and target tissues

#### Plasma levels and inter-individual variations

An average non-vegetarian diet in European countries is usually low in soy food. As a consequence, the mean isoflavone intake is marginal (e.g., 0.9 ± 2.3 mg/day; Zamora-Ros et al. 2013a), and the measured mean total isoflavone (sum of daidzein and genistein) plasma concentrations were below 10 nmol/L (Peeters et al. 2007).

Even in the case of European vegetarians and vegans, mean isoflavone plasma concentrations are lower than those of Asians eating a traditionally based diet. In the European Prospective Investigation into Cancer and Nutrition (EPIC) Oxford cohort, representing vegetarians and vegans throughout the UK, mean plasma concentrations of 79 and 148 nmol/L for daidzein and genistein, respectively, were reported (Peeters et al. 2007). In contrast, in Asian populations consuming a traditional diet including soy-based food, mean plasma concentrations of total isoflavones (sum of genistein and daidzein) are in the range of 525–775 nmol/L (Morton et al. 2002; Uehara et al. 2000; Yamamoto et al. 2001). Moreover, mean intakes of daidzein and genistein were reported to be 18.3 and 31.4 mg/day, leading to plasma concentrations of 120 and 480 nmol/L, respectively (Yamamoto et al. 2001).

Several human intervention studies investigated isoflavone plasma concentrations after intake of a defined amount of isoflavones from soy foods or from soy extract (SKLM 2009). However, the isoflavone concentration in plasma can hardly be predicted based on the dose of ingested isoflavones, since several extrinsic and intrinsic factors clearly influence their bioavailability. Identified extrinsic factors are directly related to the isoflavone source, such as food texture (solid or liquid) and nutrient composition, formulation of the dietary supplement, or the chemical form in which the isoflavones are present (Cassidy et al. 2006; Mortensen et al. 2009; Ruefer et al. 2008; Setchell et al. 2005; Yuan et al. 2012b).

Furthermore, inter-individual variability regarding the absorption efficiency and the rate of microbial biotransformation of isoflavones are suggested to be important intrinsic factors. Large inter-individual variation in plasma concentration after a defined isoflavone intake was demonstrated in several human intervention studies (Mathey et al. 2006; van der Velpen et al. 2014; Wiseman et al. 2004). Mathey et al. (2006) reported that the plasma concentrations of genistein and daidzein were highly variable in postmenopausal women after intake of 100 mg/day soy isoflavones (as cereal bars and yoghurt enriched with soy-extract) for 30 (*n* = 27) and 60 days (*n* = 12). The mean total daidzein plasma concentration (after 30 days) was reported to be 1.53 µmol/L, with the minimum and maximum values ranging from 0.43 to 4.27 µmol/L. The mean genistein plasma concentration was found to be 2.44 µmol/L, with minimum and maximum values ranging from 0.92 to 6.08 µmol/L (Mathey et al. 2006).

However, up to date little is known regarding the mechanisms responsible for differences in the uptake efficiency of isoflavones. One may speculate that genetic variations in transporters and phase II enzymes relevant for isoflavone conjugates and isoflavone aglycones, respectively, are involved (Wakeling and Ford 2012). Epigenetic mechanisms that control the expression of such genes may also contribute to the large inter-individual variabilities in the kinetics observed (Hirota et al. 2017). Furthermore, the biotransformation and degradation by the human gut microbiota seem to play a key role. Since the composition of the gut microbiota considerably differs between individuals, the type and extent of transformation reactions catalysed by microbial enzymes are highly variable and result in quantitatively and qualitatively different isoflavone derived microbial metabolite profiles.

This is shown in detail for equol, leading to the scientifically accepted classification of the equol producer and non-equol producer as already mentioned (see “Metabolism”). However, it is also relevant for other microbial degradation products, such as dihydrogenistein, dihydrodaidzein, *O*-DMA, 6′-OH-*O*-DMA, and 4-ethylphenol, although less extensive datasets are available for these metabolites.

#### Tissue levels in experimental animals and in humans

To assess the biological activity and potential health risks, it is crucial to know whether ingested isoflavones actually reach the target tissues, and, if so, which isoflavone metabolites are qualitatively and quantitatively present in the corresponding tissue. However, up to now isoflavones and their metabolites have only rarely been determined in human or even in experimental animal tissues.

A study by Chang et al. (2000) showed a dose-dependent increase in the total genistein levels in plasma and selected tissues including breast and thyroid gland after exposure of female Sprague Dawley rats to a diet fortified with various genistein levels (0, 5, 100, or 500 ppm). It was reported that the total genistein concentrations in plasma of female rats measured after enzymatic deconjugation were less than 0.01, 0.10 ± 0.008, 0.94 ± 0.21, and 7.94 ± 2.47 µmol/L, respectively. In line with this observation, the total genistein concentration increased in a statistically significant and dose-dependent manner, reaching 2.39 ± 0.34 pmol/mg (= µmol/kg) in the mammary gland and 1.15 ± 0.23 pmol/mg in the thyroid gland in the group exposed to the diet with 500 ppm genistein. The fraction of free genistein (aglycone) was 49% in the mammary gland tissue and 18% in the thyroid gland when compared to less than 5% in the plasma (Chang et al. 2000). Data in humans also indicate that isoflavones are able to reach the thyroid gland and enter follicles, where they are iodinated and then secreted. The study by Sosvorova et al. (2012) reported that iodinated isoflavones in human urine were detected after 3-month supplementation (one pill containing 40 mg of isoflavonoids twice a day).

Only four studies are available, all with a small number of volunteers, in which the isoflavone levels in breast tissue of women after aesthetic breast surgery were measured. The two older studies measured isoflavone levels after enzymatic cleavage of the isoflavone conjugates with glucuronidase/sulfatase, therefore, not being able to differentiate between the aglycone and the corresponding phase II metabolite (Hargreaves et al. 1999; Maubach et al. 2004). Furthermore, it was shown that isoflavone contents in tissue, e.g., breast tissue, can be substantially underestimated using enzymatic hydrolysis (Gu et al. 2005). The more recently published studies also provided data of non-hydrolysed sample measurements (Bolca et al. 2010; Kulling et al. 2017). The study results are summarised in Table 1 and will be briefly discussed.

**Table 1:** Summary of the results of human studies reporting isoflavone concentrations in serum or plasma and breast tissue in the case of women undergoing an aesthetic breast reduction surgery. In all studies, the women ingested a defined dose of isoflavones for a few days before surgery

Study	IF source, daily IF doses, study duration (days)	No. of volunteers	IF serum/plasma concentration in nmol/L (in total, calculated as aglycone equivalents)	IF breast tissue level in pmol/g (= nmol/kg) (in total, calculated as aglycone equivalents)
Hargreaves et al. (1999**)**	Soy protein (60 g) 45 mg IF^a^/day 14 days	*n* = 27 (serum) *n* = 8 (breast tissue)		*Breast tissue* ^d^
			GEN (mean): = 555^b,c^	GEN (mean): NA^c,f^
			DAI (mean): = 315^b,c^	DAI (mean): 107 ± 92^c^
			Equol (mean): = 124^b,c^	Equol (mean): 22 ± 91^c^
Maubach et al. (2004**)**	Soy supplement 100 mg GEN + GENG^g^/day 37 mg DAI + DAIG^g^/day 5 days	*n* = 9		*Breast tissue* ^d,e^
			GEN (median): 120^c^	GEN (median): ND**^,c^
			GEN (max): 440^c^	GEN (max): ND**^,c^
			DAI (median): ND*^,c^	DAI (median): 7^c^
			DAI (max): 740^c^	DAI (max): 111^c^
			Equol (median): ND^c^	Equol (median): 2^c^
			Equol (max): 4210^c^	Equol (max): 36^c^
			Time of blood sampling	Time of the surgery
			> 12 h after last IF intake	> 12 h after last IF intake
Bolca et al. (2010**)**	Soy milk 51 mg GEN^h^/day 16 mg DAI^h^/day 5 days	*n* = 11		*Glandular tissue*
			GEN (mean): 797 ± 237^c^	GEN (mean): 284 ± 36^c^
			DAI (mean): 196 ± 53^c^	DAI (mean): 57 ± 11^c^
			Equol (n = 4, mean): 591 ± 118^c^	Equol (n = 1): 559^c^
	Soy supplement 16 mg GEN^h^/day 53 mg DAI^h^/day 5 days	*n* = 10	GEN (mean): 218 ± 28^c^	GEN (mean): 149 ± 11^c^
			DAI (mean): 316 ± 100^c^	DAI (mean): 89 ± 16^c^
			Equol (n = 3, mean): 901 ± 81^c^	Equol (n = 1): 446^c^
			*Main metabolites*	*Main metabolites*
			GEN-7-glucuronide	GEN-7-glucuronide
			DAI-7-glucuronide	DAI-7-glucuronide
Kulling et al. (2017**)**	Soy supplement 1.0–1.4 mg IF^i^/kg bw 7 days	*n* = 6		*Glandular tissue*
			GEN (mean): 1098 ± 519	GEN (mean): 427 ± 367
			DAI (mean): 643 ± 248	DAI (mean): 294 ± 143
			*Main metabolites*	*Main metabolites*
			DAI-7-glucuronide-4′-sulfate	DAI-7-glucuronide-4′-sulfate
			GEN-7-glucuronide-4′-sulfate	GEN-7-glucuronide-4′-sulfate
			GEN-7,4′-diglucuronide	GEN-7,4′-diglucuronide

*IF* isoflavone, *ND* not detectable, *GEN* genistein, *GENG* genistin, *DAI* daidzein, *DAIG* daidzin, *GLY* glycitein, *GLYG* glycitin, *bw* body weight

*< 6.25 nmol/L; **< 12.5 nmol/L

^a^Not further specified

^b^Approximately value taken from the graph in (Hargreaves et al. 1999)

^c^After enzymatic hydrolysis with glucuronidase/sulfatase

^d^Breast tissue not further specified, mixture of glandular and adipose tissue

^e^Values are given in nmol/L

^f^NA, not available because of technical problems during measurement

^g^More than 90% as glycosides

^h^Values given as aglycone equivalents; no information about the aglycone:glucoside ratio provided

^i^1 mg IF comprises of: 472 µg GENG, 361 µg DAIG, 7.8 µg GLYG, 2.9 µg acetyl-GENG, 2.7 µg acetyl-DAIG, 1.5 µg DAI, 0.8 µg GEN, 0.5 µg acetyl-GLYG; 0.5 µg GLY

Hargreaves et al. (1999) provided data from a study in which 27 women ingested a dietary soy supplement containing 45 mg isoflavones (not further characterized) for 14 days. The daidzein concentration in plasma was approximately 80 ng/mL (= 315 nmol/L, taken from the graph in the publication) and the daidzein level in the breast tissue was 27.3 ± 23.3 ng/g (*n* = 8; = 107 ± 92 nmol/kg). The genistein concentration in plasma was approx. 150 ng/mL (= 555 nmol/L, taken from the graph in the publication). As stated by the authors, genistein levels in breast tissue could not be measured due to technical problems (Table 1; Hargreaves et al. 1999).

Maubach et al. (2004) reported isoflavone plasma and breast tissue levels of women after ingestion of a soy-based dietary supplement with 100 mg genistein/genistin and 37 mg daidzein/daidzin (more than 90% as glycosides) (*n* = 9) or a placebo tablet (*n* = 19) on five consecutive evenings before aesthetic breast surgery. Only the genistein concentration was found to be significantly higher in the serum of the soy-based supplement group than in the placebo group. In breast tissue homogenates, no significant differences in the genistein or daidzein levels were reported between the two groups. In the soy-based supplement group, genistein was not detectable in breast tissue samples, whereas serum concentrations reached a maximum of 440 nmol/L (median 120 nmol/L). Daidzein concentrations reached a maximum of 110.5 nmol/L (median 7.03 nmol/L) in breast tissue homogenates and 740 nmol/L (median not detectable) in the serum. In sum, median concentrations in the breast tissue homogenates were reported to be in the low nanomolar range, whereas in the corresponding serum samples concentrations were approximately a hundred times higher (Table 1; Maubach et al. 2004). Unfortunately, single concentration values were not reported, and furthermore, no information was provided on the reliability of the analytical method, which limits the value of the study.

In a dietary intervention study by Bolca et al. (2010), healthy women were randomly allocated for 5 days to the soy milk group (*n* = 11; 16.98 mg genistein and 5.40 mg daidzein aglycone equivalents per dose, three doses per day), the soy supplement group (*n* = 10; 5.27 mg genistein and 17.56 mg daidzein aglycone equivalents per dose, three doses per day), or the control group (*n* = 10; no supplementation). The major metabolites identified in non-hydrolysed serum and breast tissue samples were the 7-*O*-glucuronides of genistein and daidzein, whereas monosulfates or sulfoglucuronides were not detected. An overall total glucuronidation of 98% was estimated. In some of the samples, the aglycone concentrations in serum and breast tissue were also determined, thereby showing that the amount of aglycones in both compartments is rather low. 12–18 h after soy milk or soy supplement intake, breast adipocytes and mammary gland epithelial cells were exposed to up to 20–25 pmol/g total isoflavone aglycones and 900–1150 pmol/g total isoflavone glucuronides (Bolca et al. 2010). Although the study has its strengths, some of the results are not fully comprehensible and not consistent with recent data, e.g., that sulfoglucuronides are quantitatively relevant phase II metabolites in human plasma (see “Isoflavone metabolism and concentrations in plasma and target tissues Metabolism”). Therefore, it is quite puzzling that these metabolites could not be detected at all. This might be a consequence of the possibly not suited analytical conditions used to detect this type of metabolites (e.g., a positive MS mode for the detection of negatively charged molecules).

In a recent study by Kulling et al. (2017), six women consumed a well-characterized soy supplement (1.0–1.4 mg genistein and daidzein aglycone equivalents/kg bw/day) for 7 days (Table 1). Homogenous glandular breast tissue was obtained by cryo-section of frozen breast tissue specimens from breast reduction surgeries. The phase II metabolite profile of genistein and daidzein was measured in plasma and tissue samples using a validated UHPLC-MS/MS method (Soukup et al. 2014). The mean genistein and daidzein concentrations in plasma (calculated as aglycone equivalents) were 1098 ± 519 and 643 ± 248 nmol/L, respectively, while the mean genistein and daidzein levels in the glandular tissue (calculated as aglycone equivalents) were 427 ± 367 and 294 ± 143 nmol/kg, respectively. The main phase II metabolites in plasma as well as in the breast were daidzein-7-glucuronide-4′-sulfate, genistein-7-glucuronide-4′-sulfate as well as genistein-diglucuronide. Thus, the phase II metabolite profile was comparable in plasma and breast tissue. The level of the aglycones was low in both compartments, in the plasma 0.3 ± 0.5% for genistein and 0.4 ± 0.5% for daidzein and in the breast 2.1 ± 3.3% for genistein and 3.3 ± 5.1% for daidzein.

Taken together, the extrapolation from isoflavone plasma concentrations to the corresponding levels in breast tissue is difficult due to the limited database. The available data in humans, in particular the results of the more recently conducted studies using more reliable or even validated methods to determine the isoflavone plasma and breast tissue levels, do not suggest that isoflavones themselves or certain metabolites accumulate in breast tissue.

Nevertheless, the available data are very limited (low total participant number and weaknesses in the study designs or the applied analyses) and further studies are needed to enlarge the database on the tissue levels of isoflavones and their metabolites, particularly in potential target organs such as breast and thyroid gland.

## Effects of isoflavones

Isoflavones, i.e., genistein and daidzein, can interact with various transport proteins, enzymes and receptors. These properties enable them to act on a number of different cellular targets and trigger a variety of biological effects, which is summarised, e.g., in the SKLM Opinion on ‘Aspects of potentially adverse effects of polyphenols/flavonoids used in isolated or enriched form’ (SKLM 2005) and in several reviews (Bennetau-Pelissero 2013; Rietjens et al. 2017). The present review focuses on the effects of isoflavones on the female breast tissue and the thyroid gland.

### Effects on the female breast

#### Mechanistic background

**Fig. 4:**
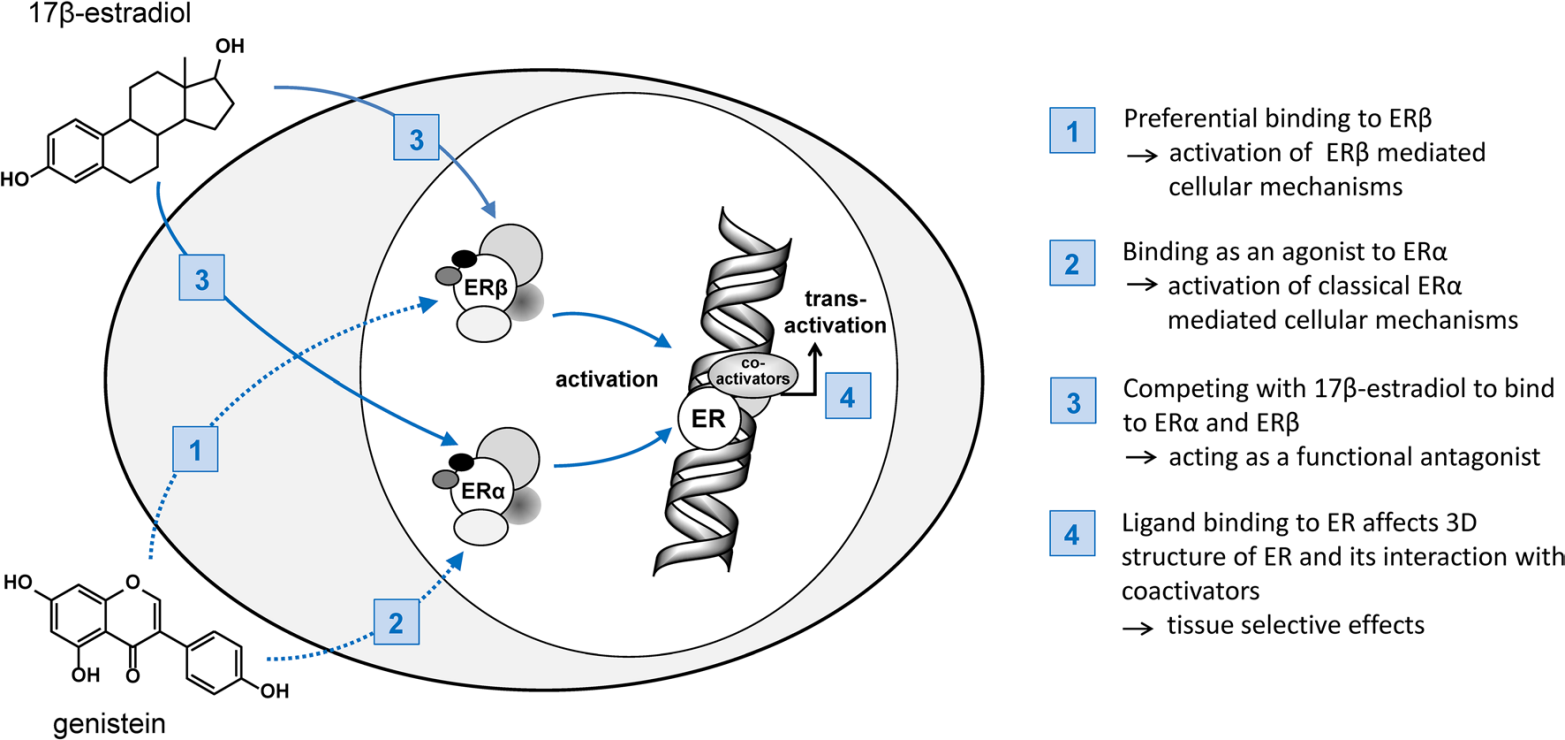
Mechanism of action of 17β-estradiol and potential interaction of isoflavones with this cascade (modified from Diel 2002). *ER* estrogen receptor, *SERM* selective estrogen receptor modulator

Upon binding to estrogens, the estrogen receptors α and β (ERα and ERβ) act as nuclear transcription factors. They show a 55% identity in their estrogen-binding domains and approximately 97% in the DNA-binding domains. Their endogenous and most potent ligand 17β-estradiol (E2) is a lipophilic steroid hormone that can passively diffuse through the cell membrane. Upon binding of E2 to ER, the ligand-binding domain undergoes conformational changes, which lead to the release of heat shock proteins that inhibit the receptors’ action in the absence of hormones. The ER subsequently translocates into the nucleus (Fig. 4). This facilitates the binding of cofactors, which promotes the interaction of ERs with their target genes. The ER makes use of a ligand-independent (AF-1) and a ligand-dependent transactivation function (AF-2), which, upon ligand binding, recruit co-regulators of gene transcription to the activated receptor (Shinkaruk et al. 2010). Both receptors interact as either homodimers or α/β heterodimers with the same conserved estrogen response elements (ERE) on the DNA (Fig. 4; Russo et al. 2016).

The two ERs are encoded by different genes and have different tissue distributions and ligand specificities. Thus, they modulate different physiological processes by regulating the transcription of the respective target genes (Paterni et al. 2014). ERα as well as ERβ are expressed in a wide range of human organs, i.e., female and male reproduction organs, lung, kidney, brain and heart. However, the ER expression pattern (ratio of the two variants and absolute expression level; Nilsson and Gustafsson 2000; Taylor and Al-Azzawi 2000) differs in various tissues or cell types of these organs (Kuiper et al. 1997). In classical estrogen-sensitive target tissues, such as the uterus and the mammary gland (Weihua et al. 2001), but also in non-classical target tissues, such as the bone (Hertrampf et al. 2007), ERα is generally assumed to be the predominantly expressed and functionally more important receptor isoform. However, one has to bear in mind that, depending on the cellular composition of an organ, e.g., in the uterus, and on the stage of the menstrual cycle, some variation in the expression of the ER may occur (Mehasseb et al. 2011). In contrast, ERβ is the dominant variant in the epithelial cells of the digestive tract (Campbell-Thompson et al. 2001; Konstantinopoulos et al. 2003; Schleipen et al. 2011), in the prostate (Weihua et al. 2001) and in the ovaries (Hegele-Hartung et al. 2004). E2 binding to ERα mediates cell proliferation (Oesterreich et al. 2001; Russo et al. 1999), e.g., in the mammary gland and uterus, and may also influence bone/skeletal homeostasis. ERβ was reported to mainly affect the central nervous and immune system. Its activation is generally considered to have anti-proliferative effects in tissues such as breast and uterus (Paterni et al. 2014). In addition, the point in time and duration of exposure to estrogens may play a pivotal role in inducing these effects. It has recently been postulated that estrogens enhance growth in breast cancer cell populations maintained in an estrogenic environment, but trigger apoptosis in cell populations adapted to long-term estrogen deprivation (Jordan 2014).

Isoflavones share structural similarities with E2 and are able to interact with both ERs (Fig. 4, no. 1–3). As a result, isoflavones are able to affect, e.g., the three-dimensional structure of ERs and, furthermore, their interaction with co-factors. Because of this mechanism and the tissue specificity, isoflavones act as partial agonists/antagonists, similar to selective estrogen receptor modulators (SERMs) (Hertrampf et al. 2005; Setchell 2001) (Fig. 4, no. 4). This in turn indicates that (anti-) estrogenic effects mediated by isoflavones might be plausible from a mechanistic point of view.

The ERα- and ERβ-specific binding affinities of some isoflavones may also be of mechanistic relevance (Fig. 4, no. 1, 2). While the natural ligand E2 possesses a similar binding affinity to both ERs, isoflavones have been shown to preferentially bind to ERβ (Kuiper et al. 1997; Setchell and Clerici 2010) (Fig. 4, no. 1). It is known that genistein possesses a higher relative binding affinity to the ER than daidzein. Equol, a microbial-derived metabolite of daidzein, has a higher binding affinity to ERα and ERβ than its precursor daidzein and its affinity to the ER is comparable to that of genistein (Muthyala et al. 2004; Pfitscher et al. 2008; Pugazhendhi et al. 2008). Mechanistically, the physiological response to equol seems to require activation of AF-1 transactivation (Shinkaruk et al. 2010). Binding preferably to a particular ER can also result in tissue-selective effects, depending on the tissue-specific expression of ERα and ERβ. In general, isoflavones and their metabolites exhibit a markedly lower affinity to both ERs when compared to E2 (Vitale et al. 2013). However, in contrast to E2, the serum concentrations of isoflavones in human beings exposed to these compounds exceed the physiological concentrations of E2.

In addition to ERα and ERβ, there are also reports on the involvement of GPER/GPCR30, a specific membrane ER belonging to the seven trans-membrane G-protein coupled receptors of the rhodopsin receptor family, in mediating responses of phytoestrogens. This receptor interacts with classical ER ligands like E2, but also with phytoestrogens (Petrie et al. 2013; Ren et al. 2016). The relative binding affinity of genistein to GPER was reported to be around 10% of that of E2, which in turn is three times higher than that of bisphenol A, an endocrine disruptive compound (Thomas and Dong 2006). In vitro, GPER upon activation has been demonstrated to trigger cell signalling pathways and growth-factor receptor cross-talk (Prossnitz et al. 2007). Based on cell culture experiments, it has been postulated that GPER may play a role in late stage carcinogenesis processes leading to triple negative breast cancer entities (Girgert et al. 2014; Prossnitz and Barton 2014), as well as in tamoxifen resistance (Ignatov et al. 2010). Overall, its biological relevance in vivo is still controversially debated, particularly since studies in GPER knock-out mice gave no hint that estrogenic responses in reproductive organs are mediated through this receptor (Otto et al. 2009; Langer et al. 2010). However, since a potential impact of GPER in mediating responses to phytoestrogens cannot be excluded in the case of a minor proportion of breast cancer sub-types, particularly triple negative tumours, the GPER pathway should be considered as another target for proliferation control.

In the past, it was a widespread opinion that phase II metabolism results in a pronounced or complete reduction of ER-mediated biological effects of isoflavones. In recent years, it has become general consensus that conjugates are not per se inactive or biologically less active than their corresponding aglycones (Beekmann et al. 2012; Williamson et al. 2005). The impact of phase II metabolism on the estrogen activity of isoflavones depends on both the type of conjugation (glucuronidation, sulfonation) and the site of conjugation.

Compared to their aglycones, isoflavone glucuronides showed a reduced estrogen activity in cell-based assays (Islam et al. 2015; Kinjo et al. 2004; Yuan et al. 2012a; Zhang et al. 1999). This is also true for daidzein-4′-sulfate, whereas the estrogen activity of daidzein-7-sulfate is either higher than or comparable to that observed for daidzein (Pugazhendhi et al. 2008; Totta et al. 2005). Only one study explored the estrogenicity of sulfoglucuronides so far. In a cell-based assay, these metabolites showed similar activities as their aglycones (Kinjo et al. 2004). In addition, the impact of the phase II metabolism of isoflavones on their estrogenicity depends on the type of ER. While 7-glucuronidation strongly reduced both the relative binding affinity to ERβ and the activation of the ERβ ligand binding domain, these effects were less pronounced for ERα (Beekmann et al. 2015; Kinjo et al. 2004). However, not all cell-based studies examined the cellular metabolism and the active transport of conjugates into or out of the cells. Without this information, these data cannot be unambiguously interpreted, as it cannot be ruled out that artefacts may have interfered with the assay.

Overall, only a few studies have explored the estrogenic activity of isoflavone conjugates. Further research is needed, especially in the case of sulfoglucuronides, which were recently shown to be the main metabolites in humans (Hosoda et al. 2011; Soukup et al. 2016a, b).

#### Animal studies

##### Long-term studies in rats

In a 2-year carcinogenicity study performed in the frame of the US National Toxicology Program (NTP), female and male Sprague Dawley rats were exposed to 0, 5, 100 and 500 ppm genistein via the diet corresponding to doses ranging from 0.2 to 44 mg/kg bw/day (NTP 2008b). Three separate study arms were performed. During the first phase, the animals of all arms were exposed from conception until the end of weaning through their mothers, who were given feed containing genistein. Thereafter, the first group was fed an untreated control diet until the end of the experiment. Following weaning, the second group was fed the genistein diet (the same as the dams) for 20 weeks and subsequently the control diet. The third group received the genistein diet (the same as the dams) starting after weaning until termination at 2 years. The authors reported ‘some evidence of carcinogenic activity of genistein in female rats of the third group based on increased incidences of mammary gland adenoma or adenocarcinoma (combined) and pituitary gland adenoma or carcinoma’ in the highest dose group (NTP 2008b). In female rats of the second group, the pituitary gland adenoma or carcinoma rates were slightly increased, and in female rats of the first group, the rates of mammary gland adenoma or adenocarcinoma (combined) were slightly increased. In the case of the latter two groups, ‘equivocal evidence of carcinogenic activity of genistein’ was reported (NTP 2008b). An early onset of aberrant oestrus cycles was found at the highest dose. These effects were reported by the authors to be consistent with an estrogenic mechanism of toxicity. In male rats investigated with the same study protocol, no increased rates of cancer were observed in any of the three exposure scenarios of the study (NTP 2008b). Noteworthy to mention is the fact that the animals showed serum levels of genistein that are achievable in humans regularly eating soy food in considerable quantities or a high-dose isoflavone supplement (NTP 2008b).

In a multigenerational study conducted by the US NTP (NTP 2008a), Sprague-Dawley rats were continuously exposed to 0, 5, 100 and 500 ppm genistein in the diet. Exposure started from the time that the *F*_0_ generation was 6 weeks through weaning of the *F*_3_ generation. This generation and subsequent ones (*F*_4_ and *F*_5_) were maintained on control feed for the remainder of the study. The authors reported that exposure to 500 ppm genistein led to a lower body weight and some alterations in the reproductive system, such as accelerated sexual maturation and altered estrous cyclicity of female rats. There were no significant alterations in the male reproductive tract, but male rats continuously exposed to 100 or 500 ppm genistein showed increased rates of mammary gland hyperplasia and calcification of renal tubules (NTP 2008a). There was no evidence of a carry-over of genistein effects in the unexposed generations (NTP 2008a).

##### Animal models used to analyse the influence of isoflavones on breast cancer

To examine the influence of isoflavones on breast cancer, a number of different rodent tumour models are available, in which breast cancer is induced by an experimental manipulation or breast cancer cells are grown in immunodeficient rodents (Manning et al. 2016). Experimental induction of breast cancer can be achieved by genetic modification, the administration of a chemical carcinogen or by excess of estrogens. Inoculation models use the transplantation of tumour cells/tumour explants into susceptible rodents. The advantages and limitations of the different models with relevance to isoflavones are summarised in Table 2. As breast cancer is not a single disease entity, all these tumour models do not cover breast cancer in general, but rather model individual aspects of mammary gland carcinogenesis or breast cancer (e.g., those expressing different hormone receptors (ERs, progesterone receptors [PRs]) and Human Epidermal Growth Factor Receptor 2 [HER2]).

**Table 2:** Major advantages and limitations of rodent breast cancer models (modified from Manning et al. 2016)

Rodent model	Advantages	Limitations
Chemically induced tumours (e.g., by DMBA or MNU)	In rats, tumours are mostly ERα-positive, which is similar to humans The immune system of the animal is intact Tumours arise in an environment that includes stromal, vascular and immune cells^a^ Influence of isoflavones on tumour onset can be examined	Rat mammary gland tumours are histologically different from those arising in humans
Genetically engineered mice	Tumours specifically develop in the mammary tissue The immune system of the animal is intact Tumours arise in an environment that includes stromal, vascular and immune cells^a^	Limited genetic mosaicism and heterogeneity of the tumours Technical hurdles to monitor tumour development in situ Low throughput and high costs
Xenograft (human cancer cell lines transplanted into immunodeficient hosts)	Numerous well characterized human breast cancer cell lines representing a variety of different human tumour types are available Tumours arise in an environment that includes stromal, vascular and immune cells^a^ Tumours develop within a short period of time and are easily measurable	Immunodeficiency of the host mice^b^ Propagation of the tumour cells in a subcutaneous environment^c^ Species “discrepancy”^d^ Homogeneity of the tumour cell population^e^ Genetic and phenotypic drift of cell lines with increasing passage number
Xenograft (human tumour explants transplanted into immunodeficient hosts)	Heterogeneity of the transplanted tumour cells^a^ Possibility of modelling a variety of different tumour types Tumours arise in an environment that includes stromal, vascular and immune cells^a^ Tumours develop within a short period of time and are easily measurable	Immunodeficiency of the host mice^c^ Surgical intervention is required Species “discrepancy”^d^ Limited access to patient specimen Propagation of the tumour cells in a subcutaneous environment^c^
Allograft (allogenic cells/tissues transplanted into immunocompetent hosts)	Implanted cells/tissues are not rejected The immune system of the animal is intact Tumours arise in an environment that includes stromal, vascular and immune cells^a^ Tumours develop within a short period of time and are easily measurable	Limited number of available cell lines, which are only in part characterized Strong immunogenicity of some cell lines leads to spontaneous tumour regression Strong proliferation rate of some cell lines limits their use in long-term studies Genetic and phenotypic drift of cell lines with increasing passage number

DMBA, 7,12-dimethylbenz[a]anthracene; MNU, 1-methyl-1-nitrosourea

^a^It reflects the situation in humans

^b^It ignores the critical role of the immune system in tumour development

^c^Cell propagation does not occur at the organotypic site

^d^Human tumour cells are injected into mouse stroma

^e^In humans, tumour cell heterogeneity is observed

Mammary gland tumours can be induced by the administration of chemicals, such as 7,12-dimethylbenz[a]anthracene (DMBA) and 1-methyl-1-nitrosourea (MNU) (Medina 2007). In rats, the chemically-induced tumours are mostly ER-positive, which mimics the situation in humans, but the rat mammary gland tumours are histologically different from those arising in humans, i.e., mostly papillary in rats and lobular in humans (Russo 2015). As for the genetically engineered mouse models, mammary tumour induction is driven by a genetic manipulation that, e.g., specifically leads to the strong expression of an oncogene or to the disruption of a tumour suppressor gene in the mammary tissue (Manning et al. 2016; Pfefferle et al. 2013). It is important to note that the majority of human breast cancers are ER-positive, which is challenging to model in genetically engineered mice. Thus, there still is a need for the refinement of the existing models and the development of new ones (Dabydeen and Furth 2014). The so-called xenograft models were established by transplanting immortalised human cancer cell lines (e.g., MCF-7 or MDA-MB-231) or patient-derived tumour explants into immunodeficient host mice, while the allograft models are based on the transplantation of immortalised mouse tumour cell lines or mouse tumour tissue into syngeneic immunocompetent mice (Manning et al. 2016).

To simulate the hormonal status of postmenopausal women, rodents are ovariectomised. In this context, the following species-related differences have to be taken into account: (1) in animal studies, mostly young adult females are ovariectomised, while postmenopausal women in general are at an advanced age; (2) following ovariectomy, almost no E2 can be detected in the blood circulation, whereas in postmenopausal women E2 from extragonadal sources, such as fat tissue, is still present in blood; and (3) the animals are mostly nulliparous at the time of the ovariectomy, while postmenopausal women, who were pregnant earlier on, underwent one or several cycles of tissue remodelling in the mammary gland following weaning.

Taken together, there are a number of animal models to analyse the influence of isoflavones on breast cancer development. Each model may serve to illuminate some aspects of human breast cancer development and may mimic a selected subset of the huge variety of human breast tumours or special aspects of tumour initiation and/or tumour growth. Based on the described limitations of the different models, it has to be pointed out that one cannot deduce a priori that the effects observed in animals will also occur in humans. However, taking aspects of mode of action into account, they may be relevant.

To assess the relevance of findings in isoflavone-treated rodents for humans, certain peculiarities of rodent physiology with regard to isoflavones should be taken into account:

- All mice and rats metabolise the isoflavone daidzein to equol, which shows the highest affinity to the ER among all known isoflavones, while only about 30% of humans are equol producers (see “Isoflavone metabolism and concentrations in plasma and target tissues metabolism”; Soukup et al. 2016a, b).
- Major differences in the absorption, distribution and metabolism of isoflavones exist between rodents and humans, which might influence their biological activity (see “Isoflavone metabolism and concentrations in plasma and target tissues metabolism”; Soukup et al. 2016a, b).

##### Effect of isoflavones on breast cancer: evidence from animal studies

The effect of isoflavones on breast cancer risk has been studied in the last two decades using the above-mentioned rodent tumour models. The models applied can be divided into (1) those investigating the effect of isoflavones on tumour initiation, development and manifestation with isoflavone exposure starting before tumour onset (see Online Annex: chapter I.A, Table A) and (2) those examining the impact of isoflavones on the growth of pre-existing mammary cancer cells/tumours with isoflavone exposure starting after tumour onset (see Online Annex: chapter I.A, Table B). The studies strongly varied in design, particularly with regard to isoflavone source, dose, administration route (subcutaneous or intraperitoneal injection, gavage, and enriched diets), duration, and window of isoflavone treatment.

Models with isoflavone exposure starting before tumour onset primarily include carcinogen-induced tumour models (induced by DMBA, MNU, or ethyl methanesulfonate [EMS]), hormonally induced models and genetically engineered mouse models. Almost all studies used intact (non-ovariectomised) female rats and mice with (normal) physiological levels of E2, modelling the effect of isoflavones on breast cancer risk under premenopausal conditions. Despite considerable differences in design, the vast majority of studies did not find significant adverse effects when compared to the control animals according to the parameters investigated. Several studies even showed some preventive effects, especially in the case of prepubertal exposure to genistein (see Online Annex: chapter I.A, Table A; de Assis et al. 2011; Hilakivi-Clarke et al. 1999; Lamartiniere et al. 2002; Möller et al. 2016; Whitsett and Lamartiniere 2006). In this context, it has been reported that the sensitivity of the breast tissue towards estrogens depends on the developmental status of the organism, and is thought to be a critical determinant in breast cancer development (Blei et al. 2015; Lamartiniere 2000; Lamartiniere et al. 2002). Thus, the observed preventive effects were at least in part attributed to the ability of genistein to modify the morphology of the mammary gland, especially during pubertal breast growth (Brown et al. 2010; Messina and Hilakivi-Clarke 2009; Warri et al. 2008).

However, in a study starting before menarche of pubertal female cynomolgus monkeys receiving a soy-protein enriched diet (corresponding to a human-equivalent dose of 120 mg isoflavones per day, expressed as aglycone equivalents) for approximately 4.5 years, only a subtle effect of the soy diet on breast differentiation and ER activity was observed. The authors discussed that even this modest change might dampen estrogen responsiveness in the breast tissue later in adulthood (Dewi et al. 2013, 2016). This decrease in estrogen responsiveness was also observed in the mammary gland of adult rats, which were exposed pre-/peripubertally or life-long to an isoflavone-rich diet (Blei et al. 2015; Molzberger et al. 2013).

In contrast to the aforementioned models with isoflavone exposure starting before tumour onset, studies investigating the impact of isoflavones on the growth of existing mammary tumours (primarily xenograft and allograft models) provided contradictory results. In several studies using a xenograft model with female ovariectomised athymic nude mice implanted with ER-positive MCF-7 cells, genistein, which was either applied as pure substance or a genistein-containing soy extract, stimulated the growth of tumour cells. The effect of genistein was dose-dependent with no effect at low doses. Attention should be paid to the fact that the soy isoflavone daidzein caused a slight but statistically significant stimulatory effect on tumour growth, whereas its metabolite equol did not stimulate tumour growth in the same model under the same experimental conditions (see Online Annex: chapter I.A, Table B; Ju et al. 2001, 2006).

In some studies, no adverse effect of genistein was observed, which might be due to shorter durations of exposure when compared to the studies demonstrating adverse effects (2–4 and 11–29 weeks, respectively). Furthermore, the cultivation conditions of MCF-7 cells prior to their injection into the athymic mice appear to be relevant: In studies demonstrating a clear adverse effect of genistein, MCF-7 cells were cultivated in the presence of 1 nM E2, which is likely to increase the sensitivity of the cells to estrogen-induced proliferation (see Online Annex: chapter I.A, Table B).

In addition, the degree of soy processing seems to impact the adverse effects of genistein. In the MCF-7 xenograft model, soy flour admixed to animal feed affected tumour cell proliferation and gene expression differently than isolated genistin, the glycoside form of genistein, alone. Tumours in mice fed genistin were larger and exhibited a higher expression of oncogenes and a lower expression of tumour suppressor genes than tumours in mice fed soy flour (Allred et al. 2004; Liu et al. 2015). The underlying molecular mechanisms are so far not understood. It was hypothesized that other bioactives in soy (e.g., phytosterols, saponins, phytic acid) might influence the estrogenic effect of genistein (Allred et al. 2004; Liu et al. 2015). The potential role of glycitein, the third isoflavone in soy beside genistein and daidzein, has not been elucidated so far but might be of relevance.

Overall, the available data from animal models used to analyse the influence of isoflavones on breast cancer do not indicate that isoflavones exert tumour-initiating properties in experimental animals (Online Annex I.A, Table A). Some of the animal studies addressing the influence of isoflavones on breast cancer promotion observed a stimulating effect, especially of genistein, on the growth of already existing ER-positive breast cancer cells (Online Annex I.A, Table B). Long-term dietary exposure of intact rats to 500 ppm genistein in a NTP study (NTP 2008b) led to some evidence of carcinogenic activity.

Independently of the outcome, the relevance of animal studies to the human situation is unclear. The strengths and weaknesses of the main rodent breast cancer models used to investigate the effects of isoflavones were discussed in “Animal studies”. Moreover, the descriptions of the study design often lack information on (1) the breeding and/or maintenance diets of the experimental animals, especially regarding their isoflavone content; (2) a detailed description of the isoflavone source; (3) the isoflavone dose per animal; and (4) the isoflavone plasma and/or tissue concentration as an indicator of the internal exposure.

#### Human studies

It is known from large-scale clinical studies that a therapeutic treatment with estrogens is able to increase the breast cancer risk. In fact, an increased cancer risk associated with a hormone replacement therapy (HRT) in postmenopausal women was observed about 15 years ago (Beral and Million Women Study 2003; Bhavnani 2002; Shumaker et al. 2003). A global consensus document still points out that HRT should be decided on a case-by-case basis, weighing up benefits and risks, and should not be used during late menopause (de Villiers et al. 2013; Wunder and Pache 2013). Moreover, HRT is considered to be contraindicated in breast cancer patients (Wunder and Pache 2013). These statements on HRT additionally support concerns regarding the putative estrogenic effects of isoflavones due to their potential to interact with ERs (see “Mechanistic background”). However, in case of isoflavones an extremely large number of study participants would be necessary to draw conclusions analogous to HRT, since the influence on breast cancer risk by isoflavones is expected to be much smaller than that of HRT.

Before adoption of the last SKLM opinion in 2006, very few intervention studies investigated the effects of isoflavones on the mammary gland in humans (SKLM 2009). The major limitations of the studies available at that time were the rather short intervention periods, the small number of participants and a lack of specifications regarding the purity or identity of isoflavone preparations. In addition, the results from these studies were inconsistent and limited to premenopausal women.

The present re-evaluation by the SKLM considered data from interventional and observational studies with pre- and postmenopausal women. Relevant findings are summarised in the following subchapters. The SKLM decided to mainly consider meta-analyses or systematic reviews and to include data from individual studies only if they were not already considered in a summarising publication. The SKLM is aware that some meta-analyses or systematic reviews reveal certain limitations. For example, some of the published studies show a partial overlap regarding the pool of considered individual studies. Especially in meta-analyses, where statistics are applied on various types of included data to abstract overall results, the heterogeneity of the considered studies is important. Furthermore, for this study type it is also crucial to evaluate whether concluding results are biased by a specific study (or study type) by means of so-called sensitivity analyses. In addition, the SKLM notes that lack of appropriate correction for multiple testing is an important limitation of many studies, in particular of those that monitored numerous isoflavones and metabolites.

##### Clinical intervention studies in healthy women

Due to the heterogeneity of the available studies, it is challenging to provide a summarising evaluation of the results. The studies vary in design, for example with regard to the study population, study duration, number of study participants as well as the isoflavone source, preparation and dose. Furthermore, some of the studies have not been specifically designed to evaluate the effect of isoflavones on breast cancer-related endpoints.

Currently used surrogate markers to investigate the influence of compounds on the female breast comprise the mammographic density of breast tissue and molecular markers for proliferation activity in breast tissue (e.g., Ki-67 or thymidine labelling index). Although the relevance of these markers is not yet fully clear, they are considered suitable as a first indication of a potential influence of isoflavones on breast cancer risk. However, it should be stressed that currently there is no established biomarker for breast cancer risk.

***Influence on the mammographic density*** Fibroglandular breast tissue (epithelial cells, fibroblasts and connective tissue) appears radio-dense on a mammogram, whereas breast fat appears transparent or non-dense. The mammographic density of breast tissue is quantified as the absolute or percentage dense area on a mammogram. This value has been suggested to be a suitable surrogate marker for breast cancer risk, since it has been reported that a moderate to high mammographic density is associated with an increase in the risk of developing breast cancer when compared to a low mammographic density in both pre- and postmenopausal women (Bitto et al. 2010; Cummings et al. 2009; Hooper et al. 2010; Maskarinec et al. 2015; McCormack and dos Santos Silva 2006; Vachon et al. 2007a, b, 2015). Evidence from a large meta-analysis suggests that dense and non-dense areas on a mammogram may be independently associated with breast cancer risk (Lokate et al. 2011; Pettersson et al. 2014). The absolute or percentage dense area was reported to be associated with an increased breast cancer risk and the absolute or percentage non-dense area with a decreased breast cancer risk (Bertrand et al. 2015; Pettersson et al. 2014). The mechanistic relationship between mammographic density and breast cancer risk, however, is largely unknown. The mammographic density can be influenced by further extrinsic factors, e.g., dietary factors, HRT or a treatment with tamoxifen (Boyd et al. 1997, 2001; Greendale et al. 2003), or intrinsic factors, e.g., the phase of the menstrual cycle in case of premenopausal women. Therefore, these aspects need to be taken into account in the clinical study design. Worth mentioning is that ethnicity does not seem to play a role (Maskarinec et al. 2017).

Data on the influence of isoflavone exposure on mammographic density are limited, since the number of isoflavone exposed study participants is rather low. A meta-analysis including eight randomised controlled trials (RCTs) with a total of 1287 healthy pre- and postmenopausal women from Europe and the USA assessed the effects of isoflavone-rich foods and isoflavone extracts from soy and red clover on breast density (Table 3; Hooper et al. 2010); details of the individual studies considered in the meta-analysis are given in the Online Annex (chapter I.B; Table C). Most studies calculated mammographic percentage density. Marini et al. (2008) used a computer-assisted algorithm calculation named image mean index (IMI) that cannot be translated into percentage breast density. The meta-analysis of the studies assessing percentage breast density did not reveal an influence of dietary isoflavone intake on breast density considering all women combined or specifically postmenopausal women (see Online Annex: chapter I.B, Table D). However, a modest increase in mammographic density in the isoflavone-exposed group compared with unexposed controls was reported in premenopausal women (mean difference (MD) 1.83%, 95% confidence interval (CI) 0.25 to 3.40, *n* = 519, *p* = 0.02). No significant effect in the few considered perimenopausal women was observed. The authors reported an increase in breast density if compared to the controls in the long-term trials (3 years, two trials), but this effect did not reach statistical significance (MD 3.22%, 95% CI − 0.18 to 6.63, *n* = 241, *p* = 0.06). There was no evidence of heterogeneity between the studies either in the overall analysis or after subgrouping by menopausal status or duration (see Online Annex: chapter I.B, Table D). However, the studies included in this meta-analysis vary in study design, e.g., concerning menopausal status of the study population (pre- vs. postmenopausal), number of participants analysed (*n* = 15–150), duration of the intervention (26–156 weeks), isoflavone sources and specifications (soy foods, soy protein, tablets) and dose (40–120 mg/day).

**Table 3:** RCTs investigating the impact of soy, red clover or isoflavones on mammographic breast density (Hooper et al. 2010) (for references of the RCTs see Online Annex: chapter I.B, Table C)

RCTs*	Number of participants analysed	Geographic area	Menopausal status	Intervention			Method of assessment
				Source/form	Isoflavone dose^a^/day	Duration	
Atkinson (2004)	int 76 cont 84	Europe (UK)	Prem., postm. and perim.	Red clover tablets	44 mg	52 weeks	% Density
Marini (2008)	at 2 years int 150, cont 154 at 3 years int 71, cont 67	Europe (Italy)	Postm.	Genistein tablets	54 mg	156 weeks	IMI
Maskarinec (2002)	int 15 cont 15	United States	Prem.	Soy tablets	76 mg	52 weeks	% Density
Maskarinec (2004)	int 98 cont 103	United States	Prem.	Soy foods	50 mg	104 weeks	% Density
Maskarinec (2009)	int A 100 int B 109 cont 116	United States	Early postm.	Soy tablets	A: 80 mg B: 120 mg	104 weeks	% Density
Powles (2008)	int 119 cont 112	Europe (UK)	Prem., postm. and perim.	Red clover tablets	40 mg	156 weeks	% Density
Tice (2009)	int 20 cont 20	United States	Prem.	Soy protein powder	50 mg	26 weeks	% Density
Verheus (2008)	int 70 cont 56	Europe (The Netherlands)	Postm.	Soy protein powder	99 mg	52 weeks	% Density

*Int* intervention, *cont* control, *prem*. premenopausal, *perim*. perimenopausal, *postm*. postmenopausal, *IMI* image mean index

*eight RCTs published in 18 full text papers, one published manuscript and an abstract; for further details concerning single publications see Online Annex: chapter I.B, Table C

^a^Isoflavone dose is given as isoflavone aglycone equivalents

A systematic review by Fritz et al. (2013) investigated the impact of soy, red clover and isolated isoflavones on mammographic density (see Online Annex: chapter I.B, Table C) and further parameters (see Online Annex: chapter II). Most of the RCTs included in the above-mentioned review had already been analysed by Hooper et al. (2010; for further details concerning the respective publications see Online Annex: chapter I.B, Table C). No evidence of an increase in mammographic density following soy or red clover supplementation among both pre- and postmenopausal women was reported. They did not observe any estrogenic effect on estrogen-sensitive target tissues such as the breast tissue.

Three further studies published more recently were not included in the reviews of Hooper et al. (2010) and Fritz et al. (2013). In a non-comparative and open-labelled study, 189 postmenopausal women from Western countries (Australia, Belgium, France, Spain) ingested a commercially available standardised soy isoflavone extract providing a total of 70 mg isoflavones/day for 3 years (Palacios et al. 2010). Mammography was performed before and after 3 years of treatment. The mammograms neither revealed changes in density nor the development of a pathological phenotype as classified by the American College of Radiology (ACR; 4 = undetermined or suspect abnormality necessitating histological verification, 5 = abnormality evocative of cancer). Major limitations of the study are that a control group was not included and the intervention was not randomised and blinded.

In a randomised, double blind and placebo-controlled study, postmenopausal women received 37 mg isoflavone aglycone equivalents (30 mg of genistin, 30 mg of daidzin) together with *Lactobacillus sporogenes* (one billion spores), 240 mg calcium, 5 µg vitamin D_3_ and 250 mg glucosamine (*n* = 65) or placebo (calcium and vitamin D_3_ alone; *n* = 65) for 12 months (Colacurci et al. 2013). Mammographic density did not show significant differences between the groups after intervention.

In a randomised, double blind and placebo-controlled study postmenopausal women ingested either 250 mg of a standardised soy extract corresponding to 100 mg isoflavones/day (*n* = 40) or placebo (*n* = 40) for 10 months (Delmanto et al. 2013). Isoflavone consumption did not affect breast density (assessed by mammography) and the breast parenchyma (assessed by ultrasound).

Taken together, the reported studies analysing breast tissue density in the breast of peri- and postmenopausal women revealed no detrimental effects of isoflavone intake on this parameter. In the meta-analysis by Hooper et al. (2010), marginal adverse effects, i.e., a modest increase in mammographic density due to isoflavone intake and limited to premenopausal women, were described, but this finding is of unclear relevance.

***Influence on Ki-67 and [***^***3***^***H]thymidine labelling index in breast tissue*** The analysis of cell proliferation in breast tissue is utilised as an established diagnostic tool in breast cancer prognosis providing useful information for therapeutic decisions regarding breast cancer patients (Luporsi et al. 2012; Wirapati et al. 2008). It can be monitored by determining the Ki-67 or [^3^H]thymidine labelling index (LI). Ki-67 is a nuclear antigen, detectable by immunostaining, which is expressed in cells in the proliferative phases of the cell cycle (G1, S, G2 and M phase), but not in cells in the resting phase (G0 phase) (Esteva and Hortobagyi 2004; Kontzoglou et al. 2013; Pathmanathan and Balleine 2013). In addition, the number of epithelial breast cells in the S phase can be quantified by labelling with [^3^H]thymidine. Specimens for further investigation of these markers can be obtained by conducting a random fine needle aspiration (rFNA). However, it has to be carefully controlled that relevant and representative specimens are sampled from the entire tissue.

When evaluating proliferation markers, it needs to be considered that menstrual cycle, age, menopausal status and oral contraceptive use may have an effect on the [^3^H]thymidine or Ki-67 LI (Hargreaves et al. 1999; Khan et al. 2012; Potten et al. 1988). Furthermore, the specific balance between proliferation, differentiation and physiologically induced cell death (apoptosis) in the mammary gland is critical for normal tissue development and homeostasis. There is evidence that changes in the cell proliferation rate as well as the ability of the cell to respond to programmed cell death is associated with tumour development and progression (Conner 2007; Kesari et al. 2003). Therefore, studies, which exclusively determine cell proliferation without taking into account potential effects on the programmed cell death, are of limited value.

In a previous short term study (14 days) in 48 premenopausal women, either ingesting 45 mg of isoflavones per day (*n* = 19) or a normal diet (*n* = 29), an increase in the breast epithelial cell proliferation rate ([^3^H]thymidine and Ki-67 labelling) was initially observed (McMichael-Phillips et al. 1998), which could not be reproduced in the follow-up study with a larger study population (*n* = 84) (Hargreaves et al. 1999).

In a randomised, double blind, placebo-controlled study, postmenopausal women ingested 60 mg isoflavones daily (*n* = 26) or a placebo (*n* = 25) for 3 months (Cheng et al. 2007). Isoflavone treatment did not alter the expression level of Ki-67.

In a randomised, double blind, placebo-controlled intervention study, healthy pre- and postmenopausal women ingested 235 mg isoflavones (*n* = 49) or placebo (*n* = 49) per day for 6 months (Khan et al. 2012). rFNA was performed and cells were examined for Ki-67 LI and cell atypia. The change in the Ki-67 LI was similar in the soy and placebo group when the data of all women in each group were combined. Following menopausal stratification, a statistically significant increase in cell proliferation from baseline to post-intervention was exclusively observed in the group of soy-treated premenopausal women (LI 1.71 vs. 2.18, *n* = 28, *p* = 0.04) but not in untreated premenopausal women (LI 1.90 vs. 1.94, *n* = 25, *p* = 0.56). However, the authors did not report any statistically significant differences when comparing the median change in Ki-67 LI between treated and control premenopausal women (0.19%; interquartile range, LI − 0.46 to 1.07, *p* = 0.31). Mid-luteal phase timing of the menstrual cycle was confirmed by rFNA in 81% of the participants. Menstrual phase remained unclear for the rest (because of irregular menstrual cycles).

Taken together, the reported studies analysing proliferation markers in breast tissue of postmenopausal women revealed no detrimental effects of isoflavone intake on these parameters. Marginal effects on proliferation markers due to isoflavone intake in premenopausal women were reported. The relevance of these findings remains unclear at the moment.

***Influence of isoflavones on other endpoints*** Several intervention studies investigated additional parameters such as the nipple aspirate fluid (NAF) volume or endpoints that may provide further information on cellular processes being modulated by isoflavones, such as the influence on the DNA methylation pattern, estrogen metabolism, changes in genetic markers of breast cancer risk, growth factors and cell signalling, markers of oxidative stress and markers of inflammation/immune response. The relevance of these parameters regarding breast cancer development and progression is unclear. Nevertheless, the results of intervention studies dealing with endpoints other than mammographic density or Ki-67 and thymidine labelling index in healthy women were also considered (summarised in Online Annex: chapter II). The available studies reported conflicting results, which at present do not allow drawing firm conclusions on whether isoflavones are indeed able to influence the aforementioned parameters.

##### Clinical intervention studies in breast cancer patients

The only RCT investigating the effects of soy in women with invasive breast cancer scheduled for resection was published by Shike et al. (2014). In premenopausal and early postmenopausal women^1^ with a diagnosis of (early stage) breast cancer (verum = 54, placebo = 50), markers of proliferation (Ki-67) and apoptosis (caspase-3) were examined. These markers were analysed in pre- and post-treatment diagnostic core biopsies following short-term (7–30 days, median duration of soy or placebo supplementation was 14 and 15 days, respectively) soy intake (26 g of soy protein powder corresponding to approximately 100 mg of isoflavones). No statistically significant differences in the primary endpoint Ki-67 labelling between pre- and post-treatment tumour samples in the soy (*p* = 0.087) and placebo group (*p* = 0.71) were reported. Moreover, a comparison of the changes between the placebo vs. soy group did not find any statistically significant differences in Ki-67 labelling (*p* = 0.21). There were no statistically significant differences between the treatment groups with regard to patient or tumour characteristics, including ER status of the tumours. However, a paired analysis of breast cancer-related gene expression levels was performed in pre- and post-treatment samples of small subgroups (*n* = 14, verum = 8, placebo = 6). Two genes were reported to be up-regulated in the soy group (p < 0.05), thus suggesting a treatment-related effect: *FANCC*, encoding a DNA repair protein, and *UGT2A1*, encoding a phase II metabolising enzyme involved in E2 metabolism as well as in the detoxification of xenobiotics, e.g., polycyclic aromatic hydrocarbons. It has been suggested that both genes may have a potential impact on breast cancer biology; however, the consequences of their increased expression with regard to human breast cancer remain unclear. Furthermore, differential gene expression was examined as a function of plasma genistein concentrations (high genistein: > 16 ng/mL vs. low genistein: < 6.8 ng/mL) by microarray analysis of tumour samples (*n* = 34, verum = 11, placebo = 23). High genistein plasma concentrations were associated with a change in a signature consisting of 126 differentially expressed genes, which was characterized by the overexpression of genes driving cell cycle and proliferation pathways (> twofold, *p* < 0.01) (Shike et al. 2014).

Further studies are needed to confirm the above-mentioned initial data on gene expression changes associated with isoflavone intake in breast cancer patients. However, one may expect that these gene expression changes are subtle and show large variations, even if a larger number of patients is analysed.

In a double blind, randomised and placebo-controlled intervention study, breast cancer patients^2^ (*n* = 66) and women with a high risk of developing breast cancer^3^ (*n* = 29) daily ingested a soy tablet containing 50 mg isoflavones for 12 months (*n* = 46) or placebo (*n* = 49) (Wu et al. 2015a). No evidence that soy supplementation influenced mammographic density in the intervention groups was reported.

##### Observational studies

Observational studies are a valuable tool (in addition to RCTs) to evaluate the association of isoflavone intake with breast cancer risk. They allow investigating breast cancer as a primary endpoint instead of surrogate markers or intermittent endpoints used in RCTs. They also usually include a higher number of participants than a RCT and, therefore, have the power to detect weaker associations, which refer to the entire population. Furthermore, if compared to RCTs, data from a prolonged period of exposure can be considered in observational studies. However, in contrast to RCTs, observational studies cannot unequivocally prove causality; they merely report associations between an exposure and the respective endpoint. In addition, observational studies are prone to the possibility of confounding. Therefore, appropriate exposure assessment and statistical analyses to control for these biases are crucial. Specifically, an association of other risk factors for breast cancer such as age, BMI (body mass index), smoking status and alcohol consumption with soy consumption has to be taken into account if present.

To identify associations between nutritional exposure and cancer risk, different designs of observational studies have been used: (i) prospective cohort studies; (ii) retrospective case-control studies, and (iii) nested case-control studies, in which incident cases in a prospective cohort are compared with selected controls of the cohort. The results of these studies can be influenced by biases, which might be restricted to or be more pronounced in a specific study type. For example, the selection bias might exert an influence especially on retrospective case-control studies. One possible source of this bias is that the control group might differ from the case group regarding characteristics that are beyond exposure but also influence the breast cancer risk, e.g., a greater proportion of health conscious and consequently healthier participants in the control group. A specific limitation, also a type of selection bias, might occur in cohort studies if study participants are not available for a follow up. The ‘loss’ of study participants could distort the results, in particular if the participants and non-participants differ regarding specific study-relevant characteristics.

In retrospective case-control studies, exposure assessment is based on a subjective recollection that might be biased by the disease (recall bias) and thus can differ between healthy controls and cancer patients. In addition, misclassification can occur in both retro- and prospective studies, for example if the mean daily intake of soy, soy proteins or isoflavones is derived from dietary assessment methods, like food frequency questionnaires (FFQ), which is the case in most of the discussed studies. This may be stated as non-differential misclassification, i.e., this will dilute the real exposure-outcome relationship. Overall, based on the described limitations, the Centre for Evidence Based Medicine (Oxford, UK) considers the strength of evidence provided by prospective cohorts to be higher than that of retrospective case-control studies. However, the final weight of a study not only depends on the study type, but also on an adequate control of biases.

Notably, many meta-analyses published up to now do not take into account the differing study designs of the individual studies when drawing overall conclusions. It is, therefore, important to examine in a meta-analysis whether the concluding results are different in prospective and retrospective case-control studies. If the observations from retrospective case-control studies cannot be confirmed by prospective studies in a comparable study population, the overall results of the meta-analysis should be interpreted with caution.

In addition, the meta-analyses are potentially biased when they combine results from Asian and Western study populations, because these populations markedly differ regarding their daily soy food consumption and the duration of exposure as well as other risk factor patterns. Chinese and Japanese populations show the highest isoflavone intakes worldwide. Their daily intake ranges from 16 to 70 mg/day (see “Exposure”). In contrast, Western (mostly European and American) populations on average consume ≤ 2 mg isoflavones per day. Since the difference between minimal and maximal isoflavone exposure is marginal in Western populations, it appears very unlikely that an effect of isoflavones can be determined in such populations. Furthermore, only such studies that used the same parameter to estimate exposure can readily be analysed in one and the same meta-analysis. If values have to be post hoc converted by the authors of a meta-analysis, it might lead to a misclassification error.

It is just as important that observational studies are properly designed, i.e., (i) the scientific question (based on comprehensible hypotheses), observed endpoints, target population and needed participant number must be defined a priori; (ii) correct and harmonised investigational instruments (e.g., design of the FFQ, sampling procedure, sample processing, tumour classification etc.) must be used, and (iii) adequate statistical analyses must be conducted.

***Isoflavone intake and breast cancer incidence***

*Evaluation of meta-analyses* Since 2006, seven meta-analyses and one systematic review examined the association between the dietary intake of isoflavones or soy proteins and the incidence of breast cancer in humans. They often included data of study populations differing in various characteristics, such as geographic area or menopausal status. In many cases, the statistical analyses were conducted on a subgroup of the included studies to answer specific questions. Because of this, the conclusions drawn by the meta-analyses were probably based on a subgroup of the total number of study participants, and declaring the total number of participants is only reasonable if the size of the respective subgroup is also specified.

As previously discussed, meta-analyses and systematic reviews that combine all study types and/or combine both Western and Asian populations should be interpreted with caution. The meta-analyses conducted with studies on Western populations did not demonstrate a statistically significant (neither positive nor negative) association with isoflavone intake, except for the beneficial effect described in the study by Chen et al. (2014; Table 4). However, in Western countries, isoflavone intake is estimated to be marginal [for details see “Exposure” and Table 2 in SKLM (2009)]. Therefore, it is not possible to draw any conclusions on the influence of isoflavones on breast cancer incidence based on these results. In contrast, the mean dietary intake is rather high in Asian populations and the exposure is assumed to result from a lifelong consumption of soy food starting early in life (see “Exposure”). In addition, there are considerable differences in the global diet composition when comparing Western and Asian populations (Morinaka et al. 2013), soy intake only accounting for a small proportion of this difference (Morinaka et al. 2013; Fernandez-Lopez et al. 2016). A systematic review by Nagata et al. (2014) summarised the data of five cohort studies and six case-control studies with Japanese women. The authors concluded that soy intake possibly decreases the risk of breast cancer among Japanese women. However, the authors of the study did not conduct statistical analyses (Nagata et al. 2014). The available meta-analyses and the systematic review suggest an inverse association between breast cancer incidence and isoflavone consumption in Asians or Asian-Americans (Table 4). This association, described as dose-dependent in some studies, was more pronounced in or limited to postmenopausal women (Table 4).

**Table 4:** Meta-analyses on the association between soy food or isoflavone intake and breast cancer incidence

References	Study type	Geographic area	Outcome		
			All women combined (prem. and postm.)	Western population (prem. and postm.)	Asian population (prem. and postm.)
Trock et al. (2006**)**	12 case-control 6 cohort or nested case-control	10 Asian 8 Western	All: ↓ prem.: ↓ > postm.: ↓	All: no association prem.: n.d. postm.: n.d.	All: no association prem.: n.d. postm.: n.d.
Qin et al. (2006**)**	14 case-control 7 cohort	10 Asian 11 Western	All: ↓ prem.: ↓ < postm.: ↓	All: n.d. prem.: n.d. postm.: n.d.	All*:↓ prem.: n.d. postm.: n.d.
Wu et al. (2008b**)**	14 case-control 5 cohort or nested case-control	8 Asian 11 Western	All: n.d. prem.: n.d. postm.: n.d.	All: no association prem.: n.d. postm.: n.d.	All*: ↓ prem.: ↓ postm.: ↓
Dong and Qin (2011**)**	14 cohort or nested case-control	4 Asian 10 Western	All: ↓ prem.: no association postm.: ↓	All: no association prem.: n.d. postm.: n.d.	All: ↓ prem.: n.d. postm.: n.d.
Xie et al. (2013**)**	15 case-control 7 cohort	12 Asian 10 Western	All: n.d. prem.: n.d. postm.: n.d.	All: no association prem.: no association postm.: no association	All: ↓ prem.: ↓ < postm.: ↓
Chen et al. (2014**)**	21 case-control 14 case-control or nested case-control	18 Asian 18 Western	All: n.d. prem.: ↓ postm.: ↓	All: n.d. prem.: no association postm.: ↓	All: n.d prem.: ↓ = postm.: ↓
Wu et al. (2015b**)**	14 case-control 1 cohort	15 Asian	All: n.d. prem.: n.d. postm.: n.d.	All: n.d. prem.: n.d. postm.: n.d.	All: ↓ prem.: n.d. postm.: n.d.

*Prem*. premenopausal, *postm*. postmenopausal, *n.d*. no data, *↓* increasing soy or isoflavone intake significantly decreased the risk of developing breast cancer; details concerning statistics are specified in the Online Annex chapter I.C, Table E

*One of the analysed studies includes Asian-Americans

The protective effect is weakened when prospective and retrospective case-control studies are considered separately in the meta-analyses. Five of the discussed meta-analyses reported that the reduction of breast cancer risk was solely observed or more pronounced in retrospective case-control studies, which generally make up the largest part of the analysed studies, than in prospective studies. However, not all meta-analyses addressing this issue additionally stratified by taking into account whether the data were obtained from Asian or Western populations, a fact that will have distorted the results. Trock et al. (2006) reported that the analysis of six cohort and nested case-control studies (four in Western or Asian-American populations and two in Asians populations) exhibited somewhat weaker associations (odd ratio [OR] 0.93) than the twelve analysed retrospective case-control studies (six in Western or Asian-American populations and six in Asian populations) (OR 0.83), although this difference was not significant. Qin et al. (2006) reported that six case-control studies (five in Asian populations and one in Asian-American populations) suggested an inverse association (risk ratio (RR) 0.72, 95% CI 0.53–0.98). In contrast, the combination of three cohort studies showed no association (RR 0.81, 95% CI 0.59–1.10), whereby the authors noted that two of them were conducted in Western women, their soy food consumption being very low (Qin et al. 2006). The study by Xie et al. (2013) reported a less pronounced risk reduction in three Asian cohort studies (RR 0.78, 95% CI 0.65–0.95) than in nine Asian case-control studies (OR 0.69, 95% CI 0.52–0.90), whereby this difference was not statistically significant. Chen et al. (2014) analysed data separately in pre- and postmenopausal women. Thirteen case-control studies carried out in premenopausal Asian women suggested an inverse association between soy isoflavone intake and breast cancer risk (OR 0.56, 95% CI 0.45–0.66). In contrast, four cohort studies in premenopausal Asian women did not reveal this association (OR 0.77, 95% CI 0.37–1.18). However, the difference between these study types was not statistically significant (OR 1.38, 95% CI 0.75–2.53, *p* = 0.31). In contrast, a significant difference (OR 1.68, 95% CI 1.02–2.77, *p* = 0.04) was reported in postmenopausal Asian women. Thirteen case-control studies showed a strong inverse association (OR 0.50, 95% CI 0.34–0.66) and five cohort studies did not (OR 0.84, 95% CI 0.54–1.14) (Chen et al. 2014). Wu et al. (2015b) reported that the meta-analysis of fourteen case-control studies in Asian women revealed a protective effect (OR 0.66, 95% CI 0.48–0.92), while the only included cohort study in Asian women did not reveal an association (OR 1.09, 95% CI 0.60–1.98). Three of the studies did not statistically address stratification according to the study design, or did not observe this bias in retrospective case-control studies. The meta-analyses of Dong and Qin (2011) exclusively considered prospective studies and reported no association in Western populations and an inverse association in Asians. In the study by Wu et al. (2008b), seven case-control studies conducted with Asians suggested a reduction in the risk of breast cancer associated with a high soy intake (OR 0.75, 95% CI 0.62–0.89). The only considered cohort study (also with Asians) reported an even more pronounced protective effect when the highest level of isoflavone intake (~ 20 mg or more isoflavones per day) was compared with the lowest intake (~ 5 mg or less isoflavone per day) (OR 0.46, 95% CI 0.25–0.84) (Wu et al. 2008b).

In summary, the detailed evaluation of the available meta-analyses shows that prospective studies on average reported higher OR or RR (ranging from 0.46 to 1.09) when compared to retrospective case-control studies (ranging from 0.50 to 0.83). However, adverse effects were never described. Since most of the meta-analyses did not stratify regarding the study population (Western/Asian) and study type (prospective/retrospective), these results should be interpreted with caution for a final evaluation of the risk.

*Evaluation of the prospective cohort studies* To convincingly exclude the occurrence of adverse effects due to isoflavone intake on breast cancer incidence in observational studies, the data of the prospective studies in the discussed meta-analyses (see Table 4) were examined in more detail. The results were differentiated according to the study population [Asian populations (Table 5) vs. Western (Table 6)] and exposure assessment [by FFQ (Tables 5, 6) vs. determination of urinary excretion/plasma concentration (Table 7)]. In studies using specimens, such as plasma or urine, the exposure determination is based on so-called biomarkers of exposure. In the case of isoflavones, the parent compounds or the corresponding metabolites (see “Isoflavone metabolism and concentrations in plasma and target tissues metabolism”) are used. However, the explanatory power of these markers depends on their ability to reflect the dose ingested, which may vary between individuals (see “Isoflavone metabolism and concentrations in plasma and target tissues metabolism”), and on the availability of appropriate analytical methods for their determination in biological matrices. These aspects are still under discussion (Rienks et al. 2017; Zamora-Ros et al. 2014).

**Table 5:** Prospective studies with Asian cohorts that analysed the association between breast cancer incidence and dietary intake of isoflavones

Reference	Cohort	*n*/*N*	Exposure	Effect estimate (CI) all	*P* for trend	Effect estimate (CI) prem.	*P*	Effect estimate (CI) postm.	*P*
Key et al. (1999**)**	Japanese, Hiroshima and Nagasaki	427/34,759	Tofu ≤ 1/week vs. ≥5/week	RR 1.07 (0.78–1.47)	0.712	RR 1.16* (0.56–2.38)	n.i.	RR 1.05* (0.73–1.49)	n.i.
			Miso soup ≤ 1/week vs. ≥5/week	RR 0.87 (0.68–1.12)	0.306	RR 1.03* (0.61–1.72)	n.i.	RR 0.83* (0.63–1.10)	n.i.
Lee et al. (2009**)**	Chinese, Shanghai	592/73,223	Soy isoflavones (mg/day), lowest vs. highest quintile, ≤ 15.93 vs. ≥54.97	RR 0.81 (0.61–1.07)	0.091	RR **0.44** (0.26–0.73)	**< 0.001**	RR 1.09 (0.78–1.52)	0.8
Nishio et al. (2007**)**	Japanese	145/30,454	Tofu < 3 times/week vs. almost daily	HR 1.14 (0.74–1.77)	0.55			HR 1.43 (0.81–2.52)	0.23
			Miso soup < 1 time/day vs. ≥2 cups/day	HR 1.01 (0.65–1.56)	0.94			HR 0.92 (0.52–1.62)	0.76
Wada et al. (2013**)**	Japanese, Takayama	172/15,607	Isoflavones (mg/day) mean of lowest vs. highest quartile, 19.9 vs. 67.4	HR 0.67 (0.44–1.03)	0.25	HR 1.52 (0.63–3.65)	0.14	HR **0.52** (0.32–0.85)	**0.046**
Wu et al. (2008a**)**/ Butler et al. (2010**)**	Chinese, Singapore	629/35,303	Soy isoflavones (mg/1000 kcal) < 10.6 vs. ≥10.6	RR **0.82** (0.7–0.97)	**0.019**	RR 1.04 (0.77–1.40)	0.82	RR **0.74** (0.61–0.9)	**0.003**
			Soy isoflavones (mg/day) median of lowest vs. highest quartile, 4.6 vs. 33.9					HR 0.86 (0.64–1.16)	0.15
			Total soy isoflavones (mg/1000 kcal/day) median of lowest vs. highest quartile 6.9 vs. 14.9	HR 0.82 (0.63–1.05)	0.03	HR 1.09 (0.68–1.73)	0.91	HR **0.7** (0.51–0.95)	**0.01**
Yamamoto et al. (2003**)**/ Iwasaki et al. (2008**)**	Japan **	179/21,852	Isoflavones (mg/day) mean of lowest vs. highest quartile, 6.9 vs. 25.3	RR **0.46** (0.25–0.84)	**0.043**	RR 0.66 (0.25–1.7)	0.97	RR **0.32** (0.14–0.71)	**0.006**
		144/288**	Genistein (mg/day) median of lowest vs. highest quartile, 15.7 vs. 27.3	OR 0.58 (0.29–1.18)	0.21	OR 0.62 (0.21–1.84)	0.43	OR 0.52 (0.19–1.42)	0.31
			Daidzein (mg/day) median of lowest vs. highest quartile, 9.4 vs. 17.1	OR 0.67 (0.33–1.39)	0.34	OR 0.67 (0.22–2.03)	0.53	OR 0.64 (0.23–1.72)	0.43
Li et al. (2005**)**	Chinese, Shanghai	155/1070^***^	Soy food (times/year) < 121 vs. >369	OR 1.0 (0.5–1.9)	0.81				

*Prem*. premenopausal, *postm*. postmenopausal, *n* number of cases, *N* number of controls; *HR* hazard ratio, *RR* risk ratio, *OR* odd ratio, *CI* confidence interval, *n.i*. no *P* value is given in the publication

*Women < or ≥ 50 years of age were considered as pre- or postmenopausal women, respectively, **Public Health Centre Based Prospective Study, ***matched controls; bold font indicates significant values, describing positive or negative associations between breast cancer incidence and dietary intake of isoflavones

**Table 6:** Prospective studies with Western (US and European) cohorts that analysed the association between breast cancer incidence and dietary intake of isoflavones

Reference	Cohort	*n/N*	Exposure	Effect estimate (CI) all	*P* for trend	Effect estimate (CI) prem.	*P*	Effect estimate (CI) postm.	*P*
Adebamowo et al. (2005**)**	USA, Nurses Health Study II	710/90,630	Total flavonols (mg/days) median of lowest vs. highest quintile 6.8 vs. 43.8			RR 1.05 (0.83–1.34)	0.96		
Brasky et al. (2010**)**	USA, VITAL cohort	880/35,016	Soy supplement user vs. non-user			HR 1.04 (0.74–1.48)	n.i.		
Hedelin et al. (2008**)**	Sweden	1014/45,448	Total isoflavonoids lowest quartile vs. highest quartile*	RR 0.98 (0.83–1.17)	n.s.	RR 1.04** (0.81–1.34)	n.s.	RR 0.93* (0.73–1.18)	n.s.
Horn-Ross et al. (2002b**)**	USA, California Teachers Cohort Study	711/11,526	Genistein (µg/day) 20th vs. 80th percentile < 290 vs. >1100	RR 1.0 (0.7–1.3)	0.9				
Keinan-Boker et al. (2004**)**	Netherlands, Dutch EPIC cohort	280/15,555	Total isoflavone (mg/d) lowest vs. highest quartile < 0.26 vs. >0.54	HR 0.98 (0.65–1.48)	0.92				
Touillaud et al. (2006**)**	France, E3N cohort	402/26,868	Total isoflavone (mg/day) lowest vs. highest quartile < 0.022 vs. >0.112			RR 1.0 (0.76–1.31)	0.48		
Travis et al. (2008**)**	UK, British EPIC cohort	585/37,643	Total isoflavone (mg/day) <10 vs. >20***	HR 1.17 (0.79–1.77)	0.36	HR 1.31 (0.95–1.81)	0.11	HR 0.95 (0.66–1.38)	0.80
Wang et al. (2009**)**	USA, Women’s Health Study	3234/38,408	Total flavonoid (mg/day) median of lowest vs. highest quintile, 8.88 vs. 47.44	RR 1.03 (0.85–1.25)	0.79				
Wang et al. (2014**)**	USA, CPS-II Nutrition Cohort	2116/56,630	Isoflavone (mg/day) lowest vs. highest quintile ≤ 0.024 vs. > 45.0	HR 1.04 (0.91–1.20)	0.64				
Zamora-Ros et al. (2013b**)**	Europe, EPIC Cohort	11,576/334,850	Isoflavone (mg/day) lowest vs. highest quintile < 0.22 vs. > 1.36	HR 1.0 (0.91–1.10)	0.734	HR 0.94 (0.77–1.16)	0.351	HR 1.00 (0.87–1.14)	0.702

*Prem*. premenopausal, *postm*. postmenopausal, *n.s*. not significant, *n* number of cases, *N* number of controls, *HR* hazard ratio, *RR* risk ratio, *OR* odd ratio, *CI* confidence interval, *n.i*. no *P* value is indicated in the publication

*The authors stated: ‘Exposures are categorized into quartiles and the lowest quartile was used as the reference category’. No data were given regarding mean/median dietary intake of isoflavonoids within quartiles, but the authors reported: “The isoflavonoid intake in this study was low (< 0.1 mg/day) as expected,...”, **the authors of this publication stated regarding the menopausal status of study participants: “mean age of natural menopause in Swedish women is 50 years of age, and women under or above 50 years of age can be considered as pre- or postmenopausal women, respectively”, ***for pre- and postmenopausal women the exposure was between < 10 vs. > 10

**Table 7:** Prospective studies with Western (US and European) and Asian cohorts that analysed the association between breast cancer incidence and urinary isoflavone and/or isoflavone metabolite excretion or plasma concentration

Reference	Cohort	*n/N*	Exposure	Effect estimate (CI) all	*P* for trend	Effect estimate (CI) prem.	*P*	Effect estimate (CI) postm.	*P*
den Tonkelaar et al. (2001**)**	Netherlands, breast cancer screening cohort	88/268*	Urinary genistein/creatinine (µmol/mol) median of lowest vs. highest tertile, 48.4 vs. 196.6					OR 0.83 (0.46–1.51)	0.06
Grace et al. (2004**)**	UK, EPIC Norfolk	Urine 114/219* Serum 97/187*	Urinary genistein/ creatinine (µg/mmol) lowest vs. highest quartile, < 3.8 vs. >19.3	OR 1.162 (0.973–1.387)	0.097				
			Serum genistein (ng/mL) lowest vs. highest quartile < 1.3 vs. >8.5	OR 1.237 (0.976–1.569)	0.077				
			Urinary daidzein/creatinine (µg/mmol) lowest vs. highest quartile, < 7.7 vs. >37.3	OR 1.123 (0. 963–1.309)	0.138				
			Serum daidzein (ng/mL) lowest vs. highest quartile < 1.0 vs. >4.5	OR **1.220** (1.005–1.481)	**0.044**				
			Urinary equol/creatinine (µg/mmol) lowest vs. highest quartile, < 0.0 vs. >0.7	OR **1.344** (1.063–1.699)	**0.013**				
			Serum equol (ng/mL) lowest vs. highest quartile < 0.1 vs. >0.2	OR **1.455** (1.051–2.017)	**0.024**				
Verheus et al. (2007**)**	Netherlands, Dutch EPIC cohort	383/383*	Plasma genistein, lowest vs. highest tertile**	OR 0.68 (0.47–0.98)	0.07	OR 0.8 (0.38–1.69)	0.65	OR 0.69 (0.45–1.04)	0.09
			Plasma daidzein, lowest vs. highest tertile**	OR 0.83 (0.58–1.19)	0.33	OR 0.80 (0.34–1.88)	0.44	OR 0.88 (0.59–1.32)	0.59
Ward et al. (2008**)**	UK, EPIC Norfolk	Urine 198/797* Plasma 219/891*	Urinary isoflavones***	OR 1.08 (1.0–1.16)	0.055	OR ****1.30 (1.04–164)	**0.022**	OR 1.01 (0.96–1.13)	0.372
			Plasma isoflavones***	OR 1.03 (0.95–1.11)	0.479				
			Urinary daidzein***	OR 1.05 (0.99–1.10)	0.096				
			Plasma daidzein***	OR 1.04 (0.98–1.10)	0.225				
			Urinary equol***	OR 1.03 (0.99–1.06)	0.131				
			Plasma equol***	OR 1.04 (0.98–1.10)	0.167				
		Urine 95/329* Plasma 105/365*	Urinary isoflavones (ER-positive subgroup)***	OR 1.09 (0.97–1.22)	0.154				
			Plasma isoflavones (ER-positive subgroup)***	OR 1.01 (0.91–1.12)	0.818				
			Urinary daidzein (ER-positive subgroup)***	OR 1.03 (0.96–1.10)	0.468				
			Plasma daidzein (ER-positive subgroup) ***	OR 1.05 (0.97–1.13)	0.260				
			Urinary equol (ER-positive subgroup)***	OR **1.07** (1.01–1.12)	**0.013**				
			Plasma equol (ER-positive subgroup)***	OR 1.01 (0.93–1.09)	0.887				
Iwasaki et al. (2008**)**	Japan Public Health Centre Based Prospective Study	144/288*	Plasma genistein (ng/mL) median of lowest vs. highest quartile, 31.9 vs. 353.9	OR **0.34** (0.16–0.74)	**0.02**	OR 0.14 (0.03–0.69)	0.2	OR 0.36 (0.12–1.12)	0.1
			Plasma daidzein (ng/mL) median of lowest vs. highest quartile, 0 vs. 53.7	OR 0.71 (0.35–1.44)	0.54	OR 0.49 (0.15–1.57)	0.48	OR 1.16 (0.43–3.15)	0.95
Goodman et al. (2009**)**	Hawaii, Los Angeles, Multiethnic Cohort Study	251/462*	Urinary genistein (nmoles/mg creatinine), lowest vs. highest quartile < 0.022 vs. >0.647					OR 0.79 (0.49–1.28)	0.29
			Urinary daidzein (nmoles/mg creatinine), lowest vs. highest quartile, < 0.183 vs. >2.536					OR 0.76 (0.47–1.21)	0.07
			Urinary equol (nmoles/mg creatinine), lowest vs. highest quartile, < 0.001 vs. >0.014					OR 0.99 (0.62–1.56)	0.80

*Prem*. premenopausal, *postm*. postmenopausal, *n* number of cases, *N* number of controls, *HR* hazard ratio, *CI* confidence interval, *conc*. concentration

*Matched controls, **genistein and daidzein plasma levels (ng/mL; median) of pre- and perimenopausal women: 3.08 (cases)/3.75 (controls) and 2.34 (cases)/2.62 (controls), postmenopausal women: 3.78 (cases)/4.89 (controls), 2.86 (cases)/3.27 (controls); ***genistein, daidzein and equol serum levels (ng/mL; median) of full study cohort: 4.77 (cases)/5.00 (controls), 1.98 (cases)/2.00 (controls), 0.01 (cases)/0.01 (controls), genistein, daidzein and equol urine (µg/mmol/creatinine; median) of full study cohort: 6.47 (cases)/5.71 (controls), 14.63 (cases)/ 14.82 (controls), 0.011 (cases)/0.011 (controls), genistein, daidzein and equol serum levels (ng/mL; median) of estrogen receptor-positive subgroup: 4.55 (cases)/4.80 (controls), 1.89 (cases)/1.83 (controls), 0.01 (cases)/0.01 (controls), genistein, daidzein and equol urine (µg/mmol/creatinine; median) of estrogen receptor-positive subgroup: 4.47 (cases)/5.67 (controls), 13.09 (cases)/15.30 (controls), 0.46 (cases)/0.04 (controls); ****pre- and perimenopausal women; bold font indicates significant values, describing positive or negative associations between breast cancer incidence and dietary intake of isoflavones

Only two out of seven Asian cohorts indicated a statistically significant risk reduction (Wu et al. 2008a; Yamamoto et al. 2003) in all women of the study populations. The results stratified for pre- and postmenopausal women were inconsistent; three cohorts showed a statistically significant risk reduction in postmenopausal women (Butler et al. 2010; Wada et al. 2013; Wu et al. 2008a; Yamamoto et al. 2003), whereas only one study indicated a risk reduction in premenopausal women (Lee et al. 2009) and one study showed no difference (Key et al. 1999). It should be noted that the cohorts with the largest number of cases (Lee et al. 2009; Wu et al. 2008a) yielded opposite effects with regard to the menopausal status (Table 5).

In contrast, ten US and European cohort studies showed no significant association between the exposure to dietary phytoestrogens and breast cancer incidence (Table 6). In three out of six prospective studies significant associations of plasma/serum/urinary total isoflavone and/or isoflavone metabolite concentrations with breast cancer risk were reported. However, the results are inconsistent. Iwasaki et al. (2008) described a statistically significant inverse association between genistein plasma concentrations and breast cancer risk, but no association between daidzein plasma concentrations and breast cancer risk. Grace et al. (2004) reported a significant association between daidzein (serum) and equol (urine and serum) concentrations and an increased breast cancer risk, whereas no significant association was found with the urinary daidzein concentration. Nevertheless, a study by Ward et al. (2008) making use of the same cohort (EPIC Norfolk) could not totally reproduce these observations. When all study participants were considered, no significant associations were demonstrated, but when the data were stratified by breast cancer type, a marginal increase of breast cancer risk in women suffering from ER-positive tumours with elevated urinary equol concentrations was reported. However, since only one out of six investigated isoflavones/metabolites exhibited a significant association, a chance finding cannot entirely be excluded. Upon stratification by menopausal status, there was an association between increased breast cancer risk and total urinary isoflavone concentration in pre- and perimenopausal women (Ward et al. 2008). These data require confirmation in studies with greater statistical power (Table 7).

*Isoflavone-containing supplements* Notably, whether the overall soy consumption resulted from soy-based food intake or from dietary supplements was not determined in many observational studies. The explicit association between isoflavone intake via supplements and breast cancer risk was only addressed by a few observational studies recently evaluated by EFSA (2015). The evaluation was based on data from four studies performed in Western populations, three case-control studies (Boucher et al. 2013; Obi et al. 2009; Rebbeck et al. 2007) and one cohort study (Brasky et al. 2010). EFSA (2015) concluded that there was no evidence of an association between the consumption of isoflavone-containing supplements and breast cancer risk.

*Influence of the equol producer status* A limited number of observational studies investigated the influence of the equol producer status on breast cancer incidence or markers of breast cancer risk. Wu et al. reported that the concentrations of plasma equol in Asian-American women with breast cancer were similar to those in healthy controls (Wu et al. 2004). In a prospective study on a European cohort (EPIC-Norfolk cohort), an enhanced breast cancer risk was associated with increased equol concentrations in serum and urine as well as with increased daidzein concentrations in serum. However, no significant associations of daidzein in urine or *O*-DMA, genistein and glycitein in urine and plasma with breast cancer risk were reported (Grace et al. 2004). In another study, also based on the EPIC-Norfolk cohort, it was shown that the breast cancer risk was associated with increased concentrations of urinary equol but not plasma equol in patients with ER-positive tumours (Ward et al. 2008). In an US cross-sectional study with postmenopausal women, the influence of the equol producer status on breast density was analysed (Fuhrman et al. 2008). Among equol producers, those reporting an intake of at least one soy food or supplement per week had a lower percentage breast density than those consuming less than one per week, whereas among non-producers, those with a weekly intake of at least one soy-based food or supplement were associated with a higher percentage breast density than those consuming less than one per week. In a study by Verheus et al. (2007) no association between equol producer status and breast cancer risk was observed.

*Summary* Overall, the data, albeit not consistent regarding a potential risk reduction, appear to exclude an adverse effect of the exposure to dietary isoflavones at the intake levels investigated on breast cancer incidence for pre- and postmenopausal women. In a few Asian study populations exposed to higher levels of dietary isoflavones than Western populations a statistically significant risk reduction of breast cancer was reported, predominantly in postmenopausal women. This protective effect was mainly observed in retrospective case-control studies and was in many cases not confirmed by the available prospective cohort studies. Thus, the reported protective effect may reflect the selection and information bias introduced by the retrospective case-control study design (Chen et al. 2014; Trock et al. 2006).

***Isoflavone intake and breast cancer recurrence*** The question whether isoflavone intake might influence breast cancer recurrence is also of particular interest. So far, two meta-analyses are available (see Table 8). In both meta-analyses an association between isoflavone intake (low vs. high level consumption) and a reduced risk of breast cancer recurrence was found. A stratification in Asian and Western populations was not performed.

**Table 8:** Meta-analyses of observational studies on the association between soy intake and breast cancer recurrence

Reference	Study type	Geographic area	Outcome		
			All women combined (prem. and postm.)	Western population (prem. and postm.)	Asian population (prem. and postm.)
Dong and Qin (2011**)**	4 cohort	2 Asian 2 Western	All: ↓ prem.: no association postm.: ↓	All: n.d. prem.: n.d. postm.: n.d.	All: n.d. prem.: n.d. postm.: n.d.
Chi et al. (2013**)**	4 cohort	2 Asian 2 Western	All: ↓ prem.: no association postm.: ↓	All: n.d. prem.: n.d. postm.: n.d.	All: n.d. prem.: n.d. postm.: n.d.

*Prem*. premenopausal, *postm*. postmenopausal, *n.d*. no data, *↓* increasing soy or isoflavone intake significantly decreases the risk of breast cancer recurrence; for details concerning statistics see Online Annex: chapter I.C, see Table G

### Effects on the thyroid hormone system

#### Mechanistic background

**Fig. 5:**
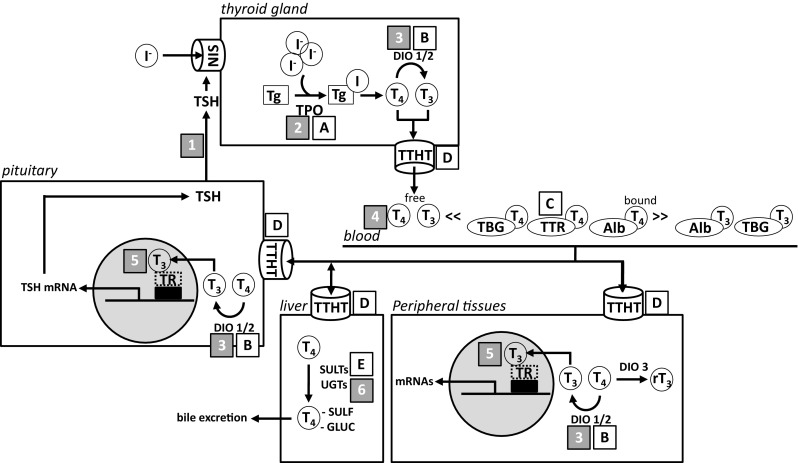
Synthesis and effects of thyroid hormones and potential targets of isoflavones. *TSH* thyroid-stimulating hormone (also known as thyrotropin), *TPO* thyroid peroxidase, *NIS* sodium/iodide symporter, *I*^*−*^ iodide, *Tg* thyroglobulin, *I* iodine, T4, 3,3′,5,5′-tetraiodo-l-thyronine (also known as l-thyroxine); T3, 3,3′,5-triiodo-l-thyronine; *DIO* iodothyronine deiodinases, *TBG* thyroxine-binding globulin, *Alb* albumin, *TTR* transthyretin, *TTHT* transmembrane thyroid hormone transporters, *TR* thyroid hormone receptor, *UGT* UDP-glucuronyltransferase, *SULT* sulfotransferases, *SULF* sulfonated thyroid hormones, *GLUC* glucuronidated thyroid hormones; filled square, key points in thyroid hormone cascade; open square, key points of thyroid hormone cascade that are potentially influenced by isoflavones

A crucial factor for the initiation of thyroid hormone synthesis is the pituitary thyroid-stimulating hormone (TSH, also known as thyrotropin). TSH induces an increase of the thyroid hormone concentration in blood by, e.g., mediating the uptake of iodide into follicular cells of the thyroid gland via the sodium/iodide symporter (NIS) (Fig. 5, no. 1) (Kaminsky et al. 1994; Riedel et al. 2001). The functional unit of the thyroid gland is the thyroid follicle. It is composed of the colloid lumen surrounded by the follicle cells (= thyrocytes). Iodide is oxidized to iodine in the colloid lumen, and then iodine is added to tyrosine residues of thyroglobulin (Tg) catalysed by thyroid peroxidase (TPO) at the cell colloid interface (Fig. 5, no. 2; Nakamura et al. 1984). Two iodinated tyrosyl residues (monoiodotyrosine and diiodotyrosine) of the thyroglobulin polypeptide chain are coupled to form the hormonally active iodothyronines; the major derivatives of thyroid hormones found in blood are 3,3′,5,5′-tetraiodo-l-thyronine (l-thyroxine, T4) and 3,3′,5-triiodo-l-thyronine (T3). T4 is converted to T3 by type 1 and type 2 iodothyronine deiodinases (DIO; DIO1, DIO2 and DIO3) of the selenoenzyme family (Fig. 5, no. 3), whereas DIO3 catalyses the conversion of T4 and T3 into the inactive forms 3,3′,5′-triiodothyronine (rT3) and 3,3′-diiodothyronine (T2). Upon fluid-phase endocytosis of Tg from the colloid followed by degradation in lysosomes, thyroid hormones are secreted by the thyrocytes into the blood stream. However, the detailed mechanism of hormone transport across the cell membrane is still under discussion (Bernal et al. 2015a; Marino and McCluskey 2000). Some proteins have been reported to act as transmembrane thyroid hormone transporters (here abbreviated: TTHT) such as MCT8, MCT10, LAT1, LAT2 or OATP1C1 (Bernal et al. 2015a, b; de Souza et al. 2015; Di Cosmo et al. 2010; Trajkovic-Arsic et al. 2010). Although T3 as well as T4 are secreted into the blood stream by the thyroid gland, the amount of T4 is much higher (ratio of approximately 1:20), thereby resulting in a higher T4 plasma concentration. Thus, peripheral T3 mainly derives from tissue T4 deiodination and only about 20% originate from thyroid follicle production. Thyroid hormones are mostly bound to plasma proteins, i.e., thyroxine-binding globulin (TBG), albumin (here abbreviated: Alb), transthyretin (TTR, formerly known as thyroxine-binding prealbumin, TBPA), and to a lesser extent to lipoproteins, which leads to a slower clearance, prolonged half-life, and higher serum concentration. Only a very small fraction of the thyroid hormones remains unbound and free (0.03% of total T4 and 0.3% of total T3 in serum). TBG binds the major fraction of circulating T4 as well as T3 (75% of serum T4 and T3). TTR binds 20% of T4 and less than 5% of T3 and is the most important thyroid hormone-binding protein in the cerebrospinal fluid (CSF). Albumin binds 5% of serum T4 and 20% of serum T3 (Fig. 5, no. 4; Feldt-Rasmussen and Rasmussen 2007). Thyroid hormones are transported into the cells of target organs, i.e., nearly all body tissues, by various TTHT. Within the cell, thyroid hormone action is mediated by nuclear thyroid hormone receptors (TRs), which exist in different isoforms and are expressed in a cell- and tissue-specific manner. Since T3 binds to TRs with a higher affinity than T4 in vitro (Samuels et al. 1974), T3 was considered to be the functional form and T4 the inactive prohormone of T3. Nevertheless, the role of T4 as a prohormone and possibly as an active hormone is still unclear. In general, T3 translocates into the cell nucleus, binds to TRs and thereby modulates gene expression (Fig. 5, no. 5). This leads to structural changes and modifications in the energy metabolism of various body tissues (Mullur et al. 2014). Besides deiodination, iodothyronines are mostly metabolised in the liver by UDP-glucuronyltransferases (UGTs) and sulfotransferases (SULTs), resulting in glucuronidated (-GLUC) and sulfonated (-SULF) thyroid hormones (Fig. 5, no. 6).

Isoflavones may interact with various different components of the thyroid hormone cascade (Fig. 5). In vitro studies have shown that soy isoflavones themselves can act as a substrate for TPO, i.e., they can be iodinated and, therefore, can act as competitive substrates (Divi et al. 1997). This hypothesis is supported by a human study reporting the detection of iodinated isoflavones in human urine after supplementation with isoflavonoids (see “Isoflavone metabolism and concentrations in plasma and target tissues metabolism”; Sosvorova et al. 2012). Furthermore, in the case of an iodine deficiency, direct inhibition of the enzyme by covalent binding of isoflavones has been reported (Fig. 5, A). The half-maximum inhibition (IC_50_) of TPO of porcine origin is already reached at a genistein and daidzein concentration of approx. 1–10 µM (Divi et al. 1997). Furthermore, genistein but not daidzein was reported to act as a potent inhibitor of human DIO1 (hDIO1) in vitro (Fig. 5, B), while no effect was observed in the case of hDIO2 and hDIO3 (Renko et al. 2015). Another target of isoflavones might be TTR. Genistein and related (iso)flavones are highly effective in inhibiting the binding of TTR to T4 and T3 (*K*_D_ = 40 nM, equimolar to T4 binding) in serum and CSF (Fig. 5, C). Thus, they may alter the kinetics and distribution of thyroid hormones in the body (Green et al. 2005; Radovic et al. 2006). At the present time it is being discussed whether isoflavones may also influence the transmembrane transport of thyroid hormones by specifically interacting with individual TTHTs such as MCT8, MCT10, LAT1, LAT2 or OATP1C1 (Fig. 5, D; Braun and Schweizer 2015; Johannes et al. 2016; Kinne et al. 2010). Genistein may even act as a powerful chaperone activator of mutated MCT8 in the Allan–Herndon–Dudley syndrome by rescuing expression of the mutated TTHT and favouring its functional expression in the plasma membrane (Braun and Schweizer 2017). Isoflavones may also impact thyroid hormone metabolism, e.g., by inhibiting sulfotransferases (Fig. 5, E; Ebmeier and Anderson 2004).

Whether effects reported in the aforementioned in vitro studies are relevant under realistic exposure conditions in humans is not yet clear, but a few studies described an influence of isoflavones on thyroid parameters, such as free T3, T4 (fT3, fT4) and TSH levels in humans (see “Human studies”).

#### Animal studies

The potential of isoflavones to influence the thyroid system in vivo was supported by results obtained in different animal studies (Chang and Doerge 2000; Divi et al. 1997; Doerge and Sheehan 2002; Silverstein et al. 2014; Sosic-Jurjevic et al. 2014; White et al. 2004). Chang and Doerge (2000) reported that the consumption of a standard soy-based rodent diet reduced TPO activity in rats by approximately 50% if compared to a soy-free diet. In a study by Silverstein et al. (2014), the effects of soy protein and isoflavones on thyroid function (on serum concentrations of total T3, free T4, and TSH) and the relationship between thyroid function and ovarian function (on the mean progesterone concentration in blood) was examined in adult female cynomolgus monkeys. In the first phase, reproductively intact animals were either fed with casein-lactalbumin or soy protein with isoflavones (approximately 9.3 mg isoflavone/kg bw/day) for 34 months; the feeding with soy was associated with increased concentrations of T3 in serum [significant positive association between T3 and genistein (*r* = 0.33, *p* = 0.03) and between T3 and total serum isoflavones (*r* = 0.31, *p* < 0.05)]. This increase in T3 was also associated with an increase in the progesterone levels (*p* < 0.01). In a second phase, half of the animals of each group were ovariectomised and continued to consume their pre-ovariectomy feed for another 34 months. The blood samples taken 9 months after ovariectomy revealed that soy in contrast to the casein-lactalbumin treatment in the first phase before ovariectomy prevented the decline in T4 after surgical menopause (Silverstein et al. 2014). It has been proposed that an altered sex steroid status may markedly influence the synthesis and stability of serum thyroid hormone distribution proteins and the regulation of the hypothalamic-pituitary-thyroid axis (Tahboub and Arafah 2009). The interference by isoflavones in this experimental non-human primate model for menopause might result in various compensatory adaptations such as the observed increased T4 and progesterone serum levels in the soy-treated group. Furthermore, no information was provided as to whether assays originally designed for the analysis of human serum samples were validated for the analysis of cynomolgus monkey serum known to have different binding properties regarding the thyroid hormones.

A recent study investigated the pharmacokinetics of isoflavones from soy infant formula in neonatal and adult rhesus monkeys. The study indicated that there are significant differences in the internal exposure when neonatal and adult monkeys are compared. Thus, the values for the dose-adjusted AUC and *C*_max_ of the active aglycone isoflavones were higher in neonates, thus indicating a higher internal exposure of young monkeys when compared to adults. Furthermore, the magnitude and frequency of equol production by the gut microbiome was significantly higher in adult monkeys (Doerge et al. 2016).

#### Human studies

Thyroid homeostasis is of relevance to many physiological processes and may be influenced by endogenous and/or exogenous (environmental/nutritional) factors. To evaluate the functioning of the thyroid hormone system in the clinic, biomarkers are used, whereby their physiological relevance is not yet entirely clear. Normally, the initial screening consists of measuring the TSH level in blood. If this parameter is elevated, free T4 concentrations are analysed to confirm the diagnosis of hypothyroidism (Garber et al. 2012, So et al. 2012). Determination of TPO antibody titres in serum is an adequate and sensitive method to specifically confirm a diagnosis of autoimmune thyroiditis, eventually leading to hypothyroidism (Hollowell et al. 2002).

As outlined above, isoflavones are discussed to affect the thyroid hormone system by interfering with many key processes operative in the hypothalamic/pituitary/thyroid axis. Under conditions of iodine deficiency, the published results support an association between an increased risk of developing goitre in infants (Hydovitz 1960; Pinchera et al. 1965) and adults when concomitantly consuming high amounts of soy foods (Doerge and Sheehan 2002). WHO/FAO recommended dietary reference values for iodine of 150 µg/day for adults (≥ 13 years) (WHO 2004) and 250 µg/day for pregnant and lactating women (WHO 2007), as summarised by EFSA (2014). These recommended intake values correspond to urinary iodine concentrations of 100 µg/L for adults and between 150 and 499 µg/L for pregnant and lactating women (WHO 2007).

In the early 1960s (anecdotal) reports on soy-induced goitre in babies and infants given soy milk instead of cow milk contributed to the mandatory iodination of baby and infant formulas (Hydovitz 1960; Pinchera et al. 1965; Van Middlesworth 1957). The impact of the long-term intake of soy protein-based infant formulas, which are retailed in Germany as “dietary food for special medical purposes”, on the development of infants has been addressed by various authors and paediatric medicine boards (Bhatia et al. 2008; ESPGHAN 2006; Koletzko et al. 2006; McCarver et al. 2011; Tillett 2010; Vandenplas et al. 2014).

##### Iodine deficiency

Importantly, human observational and interventional studies indicate that consumption of soy-based foods is unlikely to have an adverse effect on the thyroid gland system in healthy humans, if the iodine intake is adequate (Bruce et al. 2003). In this context it should be mentioned that iodine deficiency is still a worldwide problem. Zimmermann and Andersson (2012) reported that, globally, an insufficient iodine intake is observed in 29.8% of school-age children (246 million). Furthermore, a recent survey conducted with 1026 UK-resident women, which were pregnant or mother to a child aged up to 36 months, estimated the mothers’ dietary iodine intake in pregnancy using a FFQ (Combet et al. 2015). It was shown that the calculated median iodine intake without supplements (190 µg/day) was lower than the WHO’s recommended intake for pregnant women. Zimmermann et al. (2015) analysed the prevalence of iodine deficiency during pregnancy at the national level in 54 countries of the WHO European region. The iodine intake during pregnancy was adequate (as shown by a median urinary iodine concentration between 150 and 499 µg/L) in ten countries, whereas the intake was inadequate in 21 countries. Notably, in 23 of the examined countries (e.g., Germany, Uzbekistan, Kazakhstan, Hungary, and Sweden) no data were available (Zimmermann et al. 2015). Furthermore, a recent study by Johner et al. (2016) examined the iodine status of German adults nationwide by analysing 6978 spot urine samples. It was reported that the median iodine concentration in urine was 69 µg/L in men and 54 µg/L in women and that approx. 30% of the population had iodine levels in urine 40% below the population target value for iodine in the urine of 100 µg/L.

##### (Subclinical) hypothyroidism

The incidence of subclinical or overt thyroid hypofunction in women increases with age. About 10% of all women older than 55 suffer from subclinical hypothyroidism (Vanderpump and Tunbridge 2002) and many receive thyroid hormone replacement therapy (Mazer 2004). Thus, specifically postmenopausal women, representing the main target group for isoflavone-containing food supplements, are considered at risk with regard to potential side effects in the thyroid gland. In athyreotic or thyroidectomised humans requiring supplementation with T4 throughout life, potential effects of isoflavones may also deserve scrutiny. For example, this patient group includes those born without a functioning thyroid gland (one in 3500 live births worldwide) or those who had their thyroid gland completely or partially removed due to a thyroid tumour or a Morbus Basedow-type hyperthyroidism. This group, females in 75% of the cases, does not have a reserve of thyroid hormones stored in colloidal Tg anymore. Notably, isoflavones may compete with the duodenal absorption of orally administered T4 supplements.

##### Interplay with other agents interfering with the thyroid hormone system

Furthermore, it needs to be taken into consideration that other nutritive or environmental agents might interfere with the thyroid hormone system, iodide uptake as well as thyroid hormone synthesis, storage, metabolism and secretion, and therefore, there might be an interplay with isoflavones. For example, food constituents (especially those with anionic properties) such as perchlorate (Leung et al. 2014), thiocyanate and nitrate have been reported to inhibit iodide uptake in vitro through the NIS by binding to it competitively. For all three substances, additive inhibitory effects have been reported (Tonacchera et al. 2004). Perchlorate was found in groundwater and various foodstuffs (FDA 2004), e.g., tomatoes (Jackson et al. 2005), cow’s milk (Kirk et al. 2003) and eggs (Blount et al. 2008). Human exposure to thiocyanate could inter alia originate from glucosinolates, which are present in many vegetables, especially in crucifers (Eisenbrand and Gelbke 2016). Another important exposure route is cow milk, which can contain thiocyanate predominantly originating from cyanogenic glycosides and glucosinolates in the feed (Niemann and Anderson 2008). Furthermore, tobacco or its smoke contains cyanides, a thiocyanate source. Nitrate occurs ubiquitously in foods.

Thyroid hormones are pivotal in the foetal and infant development of the central nervous system. Since the thyroid gland of the foetus has not yet developed to a functional gland during the first trimester of pregnancy, the foetus depends on the transfer of iodide and iodothyronines from the mother via the placenta (Burrow et al. 1994; Landers and Richard 2017; Visser 2016). After birth, iodine is delivered via (breast) milk. Because iodine is transported into the placenta and mammary gland via NIS (Li et al. 2012), foetuses and infants are highly susceptible to the exposure to goitrogenic agents such as NIS-inhibitors, especially under conditions of iodine deficiency (Morreale de Escobar et al. 2004). For example, a study with 487 mother–child pairs suggests that high-end maternal perchlorate levels in hypothyroid/hypothyroxinemic pregnant women have an adverse effect on the cognitive development of the children (Taylor et al. 2014).

These data demonstrate that it is mandatory to accurately evaluate the impact of isoflavones on the hypothalamic/pituitary/thyroid axis and to consider additional factors, which influence thyroid function as well. Thus, a subclinical or clinical thyroid dysfunction or an insufficient iodine-supplementation might have an influence on the effects mediated by isoflavones and, therefore, need to be analysed and considered in the course of clinical studies.

##### Effects in human volunteers with thyroid dysfunction

A randomised, double-blind, crossover study in patients with subclinical hypothyroidism and adequate iodine intake investigated the effect of isoflavone supplements on the thyroid status by measuring urinary iodine excretion (Sathyapalan et al. 2011). Sixty patients were randomly divided into two groups. The participants were treated daily with either 2 or 16 mg isoflavones (sum of genistein, daidzein and glycitein) in 30 g soy protein powder for 8 weeks, followed by an 8-week washout phase. Thereafter, treatment arms were changed. Notably, six women from the high-dose group stopped supplementation because of the development of overt hypothyroidism, which was not reversible upon withdrawal of supplements. TSH levels increased by 57% (8.0 ± 0.8 vs. 13.1 ± 0.7 mU/L; *p* < 0.05) and fT4 levels decreased by 25% (12 ± 0.4 vs. 8.8 ± 0.1 pmol/L; *p* < 0.05). The urinary iodine excretion did not differ significantly between patients that developed overt hypothyroidism and the rest of the study population (median interquartile range, 272.5 ± 10.2 vs. 234 ± 6.2 µg/day; *p* = 0.2). Forty-eight participants completed the study. Within this subcohort, a difference concerning the mean TSH, fT4 and fT3 concentrations between the two intervention groups was not observed (fT3: *p* = 0.6; fT4: *p* = 0.4; TSH: *p* = 0.7). An improvement of the high blood pressure, insulin resistance and the C-reactive protein level (an inflammation and arteriosclerosis marker) was reported.

A few studies examined the effects of isoflavones on patients with (congenital) hypothyroidism. In a case-report of a woman with hypothyroidism after a near-total thyroidectomy, the need for higher oral doses of levothyroxine to reach therapeutic serum thyroid hormone concentrations was observed. It was reported that this was caused by the administration of levothyroxine concomitantly with a dietary supplement on soy protein basis that resulted in a decreased absorption of levothyroxine (Bell and Ovalle 2001). However, in a more recent study with 12 postmenopausal women that were on stable oral levothyroxine as supplementation therapy for hypothyroidism, Persiani et al. (2015) reported that the administration of 60 mg soy isoflavones did not affect the rate and extent of absorption of the concomitantly applied levothyroxine determined in plasma.

Conrad et al. (2004) retrospectively analysed patient records of infants suffering from congenital hypothyroidism that were either fed with soy formula (*n* = 8) or with non-soy formula (*n* = 70). Because of the study design, differences in treatment regimens and timing of sampling between the groups hampered the comparison of some values between the groups. The authors observed that patients of the soy diet group had a prolonged increase of the TSH levels when compared to the non-soy diet group. The median to achieve normal TSH concentrations of < 10 mU/L was 150 days (54–229 days) when compared to 40 days (19–189 days; *p* = 0.02) (Conrad et al. 2004).

A case report published by Fruzza et al. (2012) dealt with the effects of soy-based food in two children suffering from congenital hypothyroidism diagnosed during a new-born screening. Both patients were treated with levothyroxine. One child (patient 1) was fed with a soy-based formula beginning 5 days after birth for 3 weeks, whereas the second child (patient 2) received soy milk daily between the second and the fifth year of age. Both children later developed profound symptoms of hypothyroidism, as indicated by a decreased concentration of T4 and an increased concentration of TSH (patient 1: T4 = 2.6 µg/dL [normal: 11-21.5 µg/dL] and TSH = 248 µIU/mL [normal, 1–20 µIU/mL]; patient 2: fT4 < 0.4 ng/dL [normal: 0.71–1.85 ng/dL] and TSH = 248 µIU/mL). Upon medical treatment, both patients discontinued consuming the soy-based products. Although in one case readjustment of levothyroxine medication was needed, thyroid parameters in both children normalized (Fruzza et al. 2012).

The number of reports dealing with the potential risk of isoflavone supplementation in patients with an impaired function of the thyroid gland is very limited. However, the available studies indicate that isoflavones might exhibit a negative impact on the thyroid hormone system in patients with thyroid dysfunction. The mechanism of the isoflavone interaction with levothyroxine medication is not clear up to now.

##### Effects in postmenopausal and ovariectomised women

In the last years some randomised, placebo-controlled studies analysing the impact of isoflavones on postmenopausal or ovariectomised women (the surgical intervention leads to postmenopause-like conditions) were published (see Table 9). In most of these clinical intervention studies, no detrimental effects on the thyroid gland were observed. Nevertheless, four out of twelve studies reported marginal effects on some of the thyroid parameters, such as TBG, TSH, fT3 and fT4 levels or FTI (free thyroxine index). The studies listed in Table 9 had several weaknesses. Some studies did not consider the thyroid function at the beginning of the study and/or the iodine status of the participants, which potentially influenced the outcome of the intervention. Furthermore, in only three studies the primary endpoints examined were related to thyroid function (see * in Table 9). In most of the studies, the presence of chronic conditions, such as thyroid diseases, was an exclusion criterion. Apart from serum hormone analytics no information on any structural alterations of thyroid morphology by ultrasound analysis was given and thyroid autoantibody titres were not documented in most of these studies.

**Table 9:** Clinical intervention studies investigating a potential association between isoflavone or soy intake and thyroid gland dysfunction in postmenopausal or ovariectomised women

Reference	Study design (participant number and size of sub cohorts)	Menopausal status	Intervention			Outcome
			Form	Dose/day	Duration	
Duncan et al. (1999)*	int A: 17 int B: 18 cont: 18 crossover RCT (USA)	Postm.	Soy protein powder	cont: ~ 7.1 mg int A: ~65 mg int B: ~132 mg	3 × 3 mo.	No effect on total T4, fT4, total T3, fT3 and TSH Effect on TBG↓
Persky et al. (2002)*	int A: 24 int B: 23 cont: 26 RCT (USA)	Postm.	Soy protein in food	int A: 56 mg int B: 90 mg	6 mo.	Effect on (3 and 6 mo., 56 mg) T4↑, FTI↑ Effect on (3 and 6 mo., 90 mg) TSH↑ Effect on (6 mo., 90 mg) T3↑
Bruce et al. (2003)*	int: 22 cont: 16 RCT (USA) Both groups received 150 µg iodine/day	Postm.	Soy extract in tablet	90 mg	6 mo.	No effect on TSH, T3 and T4 serum conc./levels
Ryan-Borchers et al. (2008)	int A: 26 int B: 24 cont: 27 RCT (USA)	Postm.	Soy milk/ tablet	int A: soy milk (~ 71.6 mg isoflavones) int B: tablet (70 mg isoflavones)	4 mo.	No effect on TSH
Khaodhiar et al. (2008)	int A: 48 int B: 49 cont: 45 RCT (Israel)	Perim./ postm.	Capsule	int A 40 mg int B 60 mg	3 mo.	No effect on TSH, Tg, fT3, fT4, total T3 and total T4
Pop et al. (2008)	int: 18 cont: 12 RCT (USA)	Postm.	Capsule	~ 600 mg	3 mo.	No effects on T4, TSH, T3 uptake, FTI
Bitto et al. (2010)	int: 71/40 cont: 67/37 RCT (Italy)	Postm.	Tablet	genistein (54 mg)	36 mo.	No effect on THRα, THRβ, RARα and RARβ mRNA levels in peripheral blood monocytes TSH, fT3, fT4, TPO and TG serum conc./levels, anti-TMA antibody serum conc./level
Levis et al. (2011	int: 122/96/93 cont: 126/81/75 RCT (USA)	Postm.	Tablet	200 mg	24 mo.	No effect on serum conc./level of TSH and anti-TPO antibody
Steinberg et al. (2011)	int A: 135/122/119 int B: 134/123/117 cont: 134/128/126 RCT (USA)	Postm.	Tablet	int A: 80 mg int B: 120 mg	24 mo.	No effect on TSH serum level, fT4 serum level
Mittal et al. (2011)	int: 21 cont: 22 RCT (India)	Ovariect. women	Tablet	75 mg	3 mo.	No effects on TSH, fT4 and TBG serum levels, anti-TPO antibody serum level Effect on fT3↓
Alekel et al. (2015)	int A: 87 int B: 85 cont: 83 (USA)	Postm.	Tablet	int: A 80 mg int B 120 mg	36 mo.	No effect on TSH serum conc./ level, fT4 serum conc./level
Sathyapalan et al. (2017)	int: 100 cont: 100 (UK)	(Early) postm.	Snack	cont: 15 g soy protein int: 15 g soy protein + 66 mg isoflavones	6 mo.	No effect on fT3 Effect on TSH↑, fT4↑

*Int* intervention, *cont* control, *perim*. perimenopausal, *postm*. postmenopausal, *mo*. months, *ovariect*. ovariectomised, *fT3/T4* free T3/T4, *FTI* free thyroxine index, no effect, *p* > 0.05; *studies in which the examination of thyroid function was the primary endpoint; **↓**, values decreased compared to controls; **↑** values increased compared to controls; for details concerning statistics see Online Annex: chapter I.D, Table H

##### Effects in other cohorts

In recent years, a few studies dealing with the effects of isoflavones in other cohorts were published. In a study with 54 healthy young women (aged 18–25 years) the study participants consumed approx. 120 g (2 g/kg bw) of unprocessed boiled natural soybeans (Alfa Bio Slovakia) containing 1.2–4.2 mg of isoflavones per g (dry weight; Kurzer and Xu 1997; Ritchie et al. 2006) per day for 7 days (Hampl et al. 2008). The amount of soybeans consumed was chosen to approximately achieve the daily isoflavone intake in the Asian diet (20–150 mg of isoflavones per day) (Hampl et al. 2008). Serum concentrations of TSH, fT4, fT3, TPO and TG antibodies were determined before intervention, the first day following intervention and 7 days after termination of soy food consumption. The authors reported that the intervention did not lead to significant changes in the investigated parameters (Hampl et al. 2008).

Zhou et al. (2011) analysed the effects of a soy food-based diet [soy protein content with an average of 19 g/day; based on the database from the United States Department of Agriculture (USDA) and the manufacturer, the provided isoflavone content was approximately 36 mg/day] in comparison to an animal product-based diet in healthy premenopausal women. No significant effects on the TSH and free thyroid hormone levels due to the soy-containing diet were observed after 10 weeks of intervention (Zhou et al. 2011).

In an epidemiological study with 268 school children (139 girls and 129 boys; aged 8–15 years) from the Czech Republic, Milerova et al. (2006) analysed whether the serum concentrations of daidzein and genistein correlated with parameters of thyroid function. A significant positive association of genistein with the concentration of TG antibodies and a negative correlation with the thyroid volume was reported (Milerova et al. 2006).

Zung et al. (2010) described the outcome of a study on 12 children with hypercholesterolemia. The children were treated with toffee candies containing no isoflavones, 16 or 48 mg of isoflavones for 8 weeks. The analysis of thyroid parameters (TSH, T4 and total T3) showed no association with isoflavone intake (Zung et al. 2010).

A randomised, double-blind study performed in the UK investigated the effects of soy in men with type 2 diabetes mellitus and subclinical hypogonadism (Sathyapalan et al. 2016). Two hundred patients were randomly divided into two equally sized groups. The participants consumed a snack containing either 7.5 g isolated soy protein powder with 33 mg of isoflavones or 7.5 g of isoflavone-free soy protein (control) twice daily for 3 months. The TSH level significantly increased after 3 months of supplementation with the isoflavone-containing snack [Mean (SD) 1.81 (0.92) vs. 3.23 (1.03) mU/L] compared to the control supplementation [1.82 (0.93) vs. 1.96 (1.11) mU/L]. In the case of fT4, a significant decrease after the 3-month supplementation [12.68 (1.90) vs. 11.09 (2.00) pmol/L] when compared to the control supplementation [13.06 (1.74) vs. 12.74 (1.62) pmol/L] was reported. After the 3-month treatment, the fT3 level did not significantly change in either group (Sathyapalan et al. 2016).

Thyroid parameters can significantly vary during pregnancy, since the thyroid system adapts to the increased metabolic requirements. The TBG production increases, as does the total serum concentration of thyroid hormones, whereas TSH decreases. Furthermore, the β-human chorionic gonadotropin (HCG) level, which shows a similar mode of action as TSH, strongly increases in the first trimester. (Temporary) subclinical hypothyroidism is a relevant issue during pregnancy. A screening revealed that 3–10% of pregnant women suffered from subclinical hypothyroidism (Brabant et al. 2015). Thus, further research is needed to determine whether isoflavones are able to interfere with the thyroid gland function of pregnant women, and furthermore, whether they might cross the placental, blood–brain or blood-CSF barrier and thus interfere with foetal development and the foetal thyroid hormone system. As previously mentioned, foetal brain development crucially depends on thyroid hormones (Morreale de Escobar et al. 2004).

Li et al. (2011) analysed the effects of dietary isoflavone intake in 505 pregnant women. When women ingesting different amounts of isoflavones were compared, no association between dietary soy intake and thyroid parameters became apparent (Li et al. 2011). Notably, the authors stated that the women came from areas with an adequate iodine supply (median urinary iodine concentration of 180.8 µg/L). However, Combet et al. (2015) reported that the daily iodine intake of pregnant UK-resident women was insufficient (see “Human studies”).

Overall, studies in other cohorts (healthy premenopausal women, hypercholesterolemic children and pregnant women) as yet did not indicate adverse effects of isoflavone intake on thyroid parameters. Only one study (in diabetic men with subclinical hypogonadism) reported an effect of the isoflavone intake on clinical parameters. Whether this effect is physiologically relevant needs to be clarified.

##### Influence of isoflavones on thyroid carcinogenesis

Whether isoflavone intake influences the development or progression of thyroid cancer is still under discussion. In this context, only a small number of studies investigating a potential carcinogenic mode of action of isoflavones has been published so far. Yin et al. (1999) showed that various flavonoids, including genistein, induced an anti-proliferative effect in different human thyroid carcinoma cell lines. Although the underlying mechanism has not yet been fully elucidated, it has been suggested that the inhibition of TPO, e.g., by isoflavones, leads to a decrease of the circulating thyroid hormone levels. This decrease leads in turn to an increased secretion of TSH and triggers the growth of the thyroid. The persistent growth stimulus may induce the preferential selection of follicular cell clones with the potential for transformation (Williams 1990). In addition, it has been reported that E2 may induce direct effects in human thyroid cells, thereby modulating cell proliferation and organ function (Santin and Furlanetto 2011). However, it is not clear whether these effects are ER-mediated. Whether these potential effects triggered by estrogen signalling could also be expected to be of relevance in the case of estrogenic compounds such as isoflavones remains questionable at the present time.

The feeding of rats with an iodine-deficient diet containing 40% defatted soybean induced thyroid carcinoma (Kimura et al. 1976). This indicates that the role of iodine deficiency should also be discussed in the context of thyroid nodule diseases. It was hypothesized that iodine deficiency increases mutagenesis and thereby mediates nodular transformation directly by inducing the production of H_2_O_2_/free radicals or indirectly by increasing the number of cell divisions (Krohn et al. 2005).

Few observational studies have investigated an association between isoflavones and thyroid cancer. In a case-control study by Horn-Ross et al. (2002a), data on 817 thyroid cancer patients and 793 controls were considered. It was shown that the isoflavones daidzein and genistein were associated with thyroid cancer risk reduction when compared to other flavonoid compounds (OR 0.70, 95% CI 0.44–1.1, OR 0.65, 95% CI 0.41–1.0, for the highest vs. lowest quintile of daidzein and genistein, respectively; Horn-Ross et al. 2002a).

In a recent study by Xiao et al. (2014), the association between dietary flavonoid intake and thyroid cancer risk in 491,840 participants was investigated. It was reported that thyroid cancer risk was not associated with the dietary intake of isoflavones (Xiao et al. 2014).

Taken together the results from these few studies investigating the potential influence of isoflavones on thyroid cancer risk presently remain inconclusive.

## Key aspects to be considered for risk assessment

The key aspects relevant for the risk assessment of isoflavones, with special consideration of the influence on breast tissue and the thyroid hormone system, are summarised below.

### Isoflavone metabolism and concentrations in plasma and target tissues

#### Factors influencing bioavailability and biological activity

The absorption and in particular the microbial biotransformation of isoflavones have a major influence on their bioavailability. Both reveal large inter-individual variations, thereby leading to substantial differences with respect to isoflavone plasma concentrations and the quantitative and qualitative isoflavone metabolite profile. Furthermore, little is known regarding the individual variations of isoflavone absorption and biotransformation during lifetime. Since the various metabolites possess different estrogenic and anti-thyroid activities (see “Isoflavone metabolism and concentrations in plasma and target tissues metabolism” and “Mechanistic background”), the mediated biological effects might also vary between individuals. Of particular interest is the question whether the food matrix as such influences the biological activity of isoflavones.

#### Isoflavone concentration in human plasma and target tissues

The limited data indicate that isoflavones are predominantly present in breast tissue and plasma as phase II conjugates, whereas the amount of aglycones appears to be rather low. There is no indication that isoflavones accumulate in breast tissue. Preliminary data in humans indicate that isoflavones are also able to reach the thyroid gland (see “Isoflavone metabolism and concentrations in plasma and target tissues metabolism”).

#### Species differences

There are considerable species differences between humans and experimental animals specifically regarding the phase II and microbial metabolism of isoflavones. As this leads to considerably different isoflavone profiles in plasma and target tissues, it is reasonable to assume that biological activities also differ. The relative level of aglycones in plasma and target organs also differs between species. It has been reported to be low in humans and rats, but comparatively high in mice, particularly for daidzein (see “Isoflavone metabolism and concentrations in plasma and target tissues metabolism”).

### Influence of isoflavones on the female breast

#### Evidence from animal studies

Rodent tumour models were used to clarify defined questions regarding the influence of isoflavones on breast cancer development and progression and to better understand the mode of action of isoflavones. The key benefit of these animal studies lies in the possibility of identifying potential risks for humans, which for several reasons can neither be elucidated nor investigated in human studies.

Clearly, the validity of the toxicological conventional studies when performed according to the relevant guidelines (e.g., OECD) is generally agreed. However, findings in experimental animals due to isoflavone exposure cannot a priori be extrapolated to the situation in humans. The assessment of the results from animal studies should consider existing limitations of each animal model (see “Animal studies”) especially those which are specific for isoflavones, e.g., species differences in isoflavone biotransformation (see “Isoflavone metabolism and concentrations in plasma and target tissues metabolism”).

#### Evidence from human intervention studies

RCTs in general are the most suitable study type in evidence-based research, because they are able to show causal relationships. However, the available RCTs investigating the influence of isoflavones on breast cancer risk exhibit some limitations. The data from various studies are difficult to compare due to large differences regarding the study design and a rather low overall number of study participants (see “Human studies”). Furthermore, uncertainties concerning the validity of utilised surrogate markers have to be taken into account (see “Human studies”).

#### Evidence from observational studies

Observational studies are a valuable tool to evaluate the influence of isoflavone intake on breast cancer risk, since breast cancer can be investigated as a primary endpoint in comparatively large study populations. However, they merely report associations and not causal relationships. In addition, they might enclose biases in various directions and other distortive effects, which might increase the general uncertainties of the results. Furthermore, the available meta-analyses on observational studies have considerable limitations (see “Human studies”). Therefore, the data described are only helpful for risk assessment if distortive effects on the data are controlled by the study design. Since these effects where not sufficiently considered in many published meta-analyses, their results should be interpreted with caution.

#### Evidence from studies in breast cancer patients

The influence of isoflavones on an existing breast tumour might differ considerably from that on breast tissue in healthy women. Breast cancer patients and women with undetected breast cancer are considered as particular risk groups, and data from these groups are regarded to be of special relevance for risk assessment. However, the available data are rather limited and further studies are needed (see “Human studies”).

#### Is there an influence of a lifelong consumption of soy food on breast cancer risk?

In Asian countries it is assumed that the isoflavone intake mainly results from a lifelong consumption of soy food already starting early in life. It has been suggested that isoflavones may exert their potentially protective effects early in life by stimulating breast cell differentiation. Several animal studies appear to support this hypothesis (see “Animal studies”), but the underlying mechanisms are not yet fully understood.

#### Is there a differential response to isoflavones in pre- and postmenopausal women?

Some studies indicate that the time of exposure to isoflavones may play a pivotal role when assessing effects of estrogenic compounds on the female breast (see “Mechanistic background”). It has recently been postulated that estrogens enhance growth in breast cancer cell populations maintained in an estrogenic environment. These conditions might be comparable to those in premenopausal women. In contrast, estrogens trigger apoptosis in breast cancer cell populations that are adapted to long-term estrogen deprivation. These conditions might be comparable to those in postmenopausal women. However, further studies are required to investigate a differential response of pre- and postmenopausal women to isoflavones. In addition, it should be kept in mind that a breast tumour diagnosed during menopause might have begun to develop before menopause.

### Influence of isoflavones on the thyroid hormone system

#### Influence of iodine status

Potential anti-thyroid effects of isoflavones are considered to depend on the iodine status of the study participants. In this context, it should be mentioned that iodine deficiency is still a worldwide problem including Western populations (see “Human studies”). Taking into account the impact of an adequate iodine supply on the thyroid gland function, the relevance of studies on isoflavones that do not consider the iodine status of participants is questionable. At present, studies that systematically analyse the effect of isoflavones by separating it from that of iodine status on the hypothalamic/pituitary/thyroid hormone system are still missing.

#### Evidence from human studies

Only a limited number of studies are available. Many of the studies show a deficient study design (e.g., missing placebo controls, small number of study participants or missing control for iodine status). Some studies lack information on whether participants suffered from any thyroid dysfunction (such as subclinical hypothyroidism) at the beginning of the trial, which could have influenced the outcome of the studies (see “Human studies”).

#### Physiological relevance of altered clinical parameters

Blood TSH or free T4 levels are used in the clinic to evaluate thyroid gland function (see “Human studies”). These clinical parameters are also used in clinical and observational studies to assess the effects of isoflavones on the thyroid hormone system. However, at the present time, it is not entirely clear whether and to what extent an altered clinical parameter is also physiologically relevant.

#### Potential risk groups

An effect of isoflavones on the thyroid hormone system most likely could be expected in certain risk groups, which include persons with an iodine deficiency, a (subclinical) and congenital hypothyroidism as well as pregnant women due to the enhanced metabolic requirements of the thyroid system during pregnancy and the high sensitivity of the foetus to an impaired thyroid function. Postmenopausal as well as ovariectomised women are also regarded as potential risk groups, especially if their iodine supply is not adequate, since the incidence of (subclinical) hypothyroidism increases with age. It has to be noted that there might be additional risk groups, e.g., men with type 2 diabetes mellitus and subclinical hypogonadism (see “Human studies”).

## Conclusions

Conclusions arising from the re-evaluation of health effects of isoflavones in humans based on an analysis of interventional and observational human studies as well as experimental *in vitro* and animal studies are outlined below.

### Effects of isoflavones on the female breast

#### Animal and in vitro studies

Data from in vitro studies and animal experiments suggest that the estrogenic as well as the anti-estrogenic effects mediated by isoflavones are mechanistically plausible. Animal studies on the potential influence of isoflavones on tumour induction did not show significant differences in the incidence of adverse effects. Some studies found indications for preventive effects, especially in the case of a prepubertal exposure to isoflavones. In several studies addressing a potential promoting activity on the growth of already existing ER-positive mammary tumours, tumour cell growth was stimulated. However, in other studies, no such adverse effects were observed. The outcome strongly depends on the isoflavone tested, the study design as well as the animal models used. The relevance of the used animal models to understand the situation in humans remains unclear at the present time due to limitations of these models and species differences.

#### Interventional studies

Isoflavones (40–120 mg/day, for 6–36 months) did not affect the breast density and the expression of proliferation markers in breast tissue samples of healthy postmenopausal women, while marginal adverse effects on these parameters were reported for healthy premenopausal women. Again, the relevance of these markers remains uncertain at the present time.

In one pilot study the effect of isoflavone exposure on different parameters in pre- and early postmenopausal women with invasive breast cancer scheduled for resection were analysed. The expression of Ki-67 and the apoptosis marker caspase-3 was not affected by isoflavones (approximately 100 mg/day, for 7–30 days), whereas the expression of a number of breast cancer-related genes was altered when compared to the placebo group. Further studies are needed to verify these pilot results and to determine whether these effects are time- and dose-dependent.

Further intervention studies analysed various other endpoints, e.g., the volume of the nipple aspirate fluid (NAF), the expression level of growth factors or markers of oxidative stress and inflammation/immune response. Some of these endpoints have been suggested to be related to breast cancer risk, although their relevance is not yet clear. The evidence from these studies is, therefore, considered inconclusive.

#### Observational studies

The data, albeit not consistent regarding a potential risk reduction, appear to exclude an adverse effect of the exposure to dietary isoflavones on breast cancer incidence at the investigated intake levels (up to approximately 70 mg). In a few Asian study populations exposed to higher levels of dietary isoflavones than Western populations, in which soy intake is usually low, a significant breast cancer risk reduction was reported, predominantly in postmenopausal women. In most of the meta-analyses, this protective effect was mainly observed in retrospective case-control studies and was not confirmed by the available prospective studies. Thus, these effects may reflect a selection and information bias introduced by the retrospective case-control study design.

The very few data investigating the impact of isoflavone intake on breast cancer recurrence reported a protective effect in Asian as well as in Western populations. Only a few observational studies addressed the association between isoflavone intake via dietary supplements and breast cancer risk and no evidence of an association was found. Studies examining the influence of the equol producer status on breast cancer incidence are limited in number and inconclusive up to now.

### Effects of isoflavones on the thyroid hormone system

#### Animal and in vitro studies

Various in vitro and animal studies reported that isoflavones are able to interfere with different checkpoints of the hypothalamic/pituitary/thyroid system. An influence on clinical markers for thyroid function such as TSH, fT4 and fT3 was also reported in several studies. Whether these effects might be relevant under realistic exposure conditions in humans is still unclear.

#### Studies in non-risk groups

The non-risk groups include healthy, non-pregnant, premenopausal women with an adequate iodine status. Isoflavones did not lead to functional alterations in the thyroid hormone system of this population group. A study on hypercholesterolemic children did not reveal alterations of thyroid hormone blood parameters/biomarkers (TSH, T4, T3, TBG) due to isoflavone intake.

#### Studies in risk groups

The population groups in which isoflavones might influence the thyroid hormone system include individuals suffering from iodine deficiency and (subclinical) hypothyroidism, which is especially relevant for the group of postmenopausal and ovariectomised women. The few available (and limited) studies in patients with an impaired function of the thyroid gland, i.e., with (subclinical/congenital) hypothyroidism, indicate that isoflavones might exert a negative effect on the thyroid hormone system. In some studies with postmenopausal or ovariectomised women, slight alterations of the thyroid hormone blood parameters (TSH, T4, T3, TBG) following isoflavone intake were observed. These effects need further research to clarify their reversibility and physiological relevance. It has to be pointed out that the studies on postmenopausal or ovariectomised women showed limitations in their study design (especially considering the iodine status and the thyroid function of the study populations at the beginning of the study). In addition, pregnant women must be considered as a risk group. In the only study with pregnant women, no adverse effects due to the consumption of soy foods were reported. Studies in further potential risk groups are scarce. An RCT with men suffering from type 2 diabetes mellitus and subclinical hypogonadism reported an association between the intake of an isoflavone-containing snack and a significant increase of the TSH concentration in blood, whereas fT3 was not affected.

#### Influence of isoflavones on thyroid carcinogenesis

The influence of isoflavone intake on thyroid cancer risk has not yet been adequately analysed in observational studies and the evidence is inconclusive.

### Summary

The available human studies do not indicate that an isoflavone exposure as reported in the thoroughly studied Asian population or as investigated in clinical studies (i.e., about 100 mg/day) negatively influences breast cancer risk or the thyroid hormone system in healthy women. However, the SKLM points out that particular attention should be given to the susceptible risk groups. These include people with an iodine deficiency^4^ (especially during pregnancy), (subclinical) hypothyroidism, and/or (congenital) thyroid dysfunction as well as women with breast cancer or with a history of breast cancer.

As a measure of precaution, the SKLM recommends such risk groups to abstain from the intake of isoflavone-containing supplements. As a pragmatic guidance for these groups, the SKLM also recommends the dietary intake of isoflavones through consumption of soy foods not to exceed the median intake in Asian countries of around 50 mg/day considered as safe.

## Research needs

There is a need for further scientific research to be able to perform a conclusive risk assessment on the impact of isoflavones on breast tissue and the thyroid hormone system. In particular, the following topics should be addressed:

- uptake of isoflavones and metabolites as well as isoflavone and metabolite profile in target tissues, also addressing species-specific differences
- effects of isoflavone aglycones and conjugated forms on the membrane estrogen receptor GPER
- effects of isoflavone metabolites [e.g., (anti-)estrogenicity and goitrogenicity] in target tissues
- impact of the microbiota on the bioavailability of isoflavones
- development of adequate biomarkers for increased breast cancer risk
- physiologically based biokinetic (PBBK) models to predict in vivo dose–response curves for estrogenic activity in target tissues or modulation of the hypothalamic/pituitary thyroid system in humans
- impact of isoflavones on the female breast tissue in pre- and postmenopausal women investigated in RCTs with a standardised and validated study design/protocol
- impact of isoflavones on breast cancer recurrence
- systematic identification of GPER in breast cancer diagnosis
- association between isoflavone intake via supplements and breast cancer risk
- influence of isoflavones in mediating goitrogenic effects, particularly in risk groups (e.g., individuals with an iodine deficiency and subclinical hypothyroidism, patients without a functioning thyroid gland, thyroid hormone-substituted patients, pregnant women) and analysis of the underlying mechanisms
- integration/application of -omics technologies, especially metabolomics in further studies

## Electronic supplementary material

Below is the link to the electronic supplementary material.

Supplementary material 1 (DOCX 370 KB)

## Footnotes

1 Patient exclusion criteria: i.a., a history of other active malignancy within 2 years, regular soy consumption, malabsorption disorders, hormone replacement therapy, tamoxifen or raloxifene treatment in the last 3 months.

2 Time period between completion of chemotherapy/endocrine treatment and entry into the trial: from 6 months up to 4 or more years, without evidence of recurrence.

3 Five year Gail risk > 1.7% or known BRCA1/BRCA2 mutation carriers or family history consistent with hereditary breast cancer or biopsies exhibiting atypical hyperplasia.

4 According to the National Nutrition Survey II, 53% of women are below the recommended value for iodine intake in Germany, also when taking into account the use of iodised salt (NVSII (2008) Nationale Verzehrsstudie II: Teil, Ergebnisbericht: Max Rubner-Institut, Bundesforschungsinstitut für Ernährung und Lebensmittel.
