# Supplementary material for: Effects of isoflavones on breast tissue and the thyroid hormone system in humans: a comprehensive safety evaluation
Source: Arch Toxicol. 2018 Aug 21;92(9):2703–48. doi: 10.1007/s00204-018-2279-8 (PMC6132702; doi:10.1007/s00204-018-2279-8)
Supplement: Supplementary file 1 — Supplementary material 1 (DOCX 370 KB) [file 204_2018_2279_MOESM1_ESM.docx]

Annex

1. Details concerning included studies

##### I.A Effect of isoflavones on breast cancer: Evidence from animal studies

**Table A/B:** Animal studies investigating the effect of isoflavones on mammary carcinogenesis. BW, body weight; DAI, daidzein; DIN, daidzin; DMBA, dimethylbenz[*a*]anthracene; E2, 17β-estradiol; EMS, ethyl methanesulphonate; ERα, estrogen receptor α; GCP^TM^, genistein combined polysaccharide; GEN, genistein; GIN, genistin; GLY, glycitein; IF, isoflavone; MNU, 1-methyl-1-nitrosourea (=*N*-nitroso-*N*-methylurea); MPA, medroxyprogesterone acetate; SP, soy protein; SPI, soy protein isolate. *Red, green and blue arrows indicate statistically significant adverse, statistically significant beneficial and no effect, respectively, of IF treatment on mammary carcinogenesis when compared to negative control groups. **Calculated, based on given data in publication.

| **A. IF exposure started before tumour onset** | | | | | | | |
| --- | --- | --- | --- | --- | --- | --- | --- |
| **Studies in chemically-induced tumour models** | | | | | | | |
| **Animal model** | **Animal / Strain**  **(female)** | **IF source and administration route** | **IF amount in diet**  **[ppm = µg/g diet]** | **IF dose/day;**  **IF blood level Y/N?** | **Treatment period and end of experiment** | **Effect of IF treatment compared to negative control groups regarding mammary carcinogenesis*** | **Ref.** |
| **Chemically-induced tumour model**  **DMBA**  Tumour induction in offspring started on postpartum day 50 | **Rat**  Sprague-Dawley CD  Intact | GEN by **subcutaneous injection** | Soy-free diet | 5 mg GEN/rat at postpartum day 2, 4 and 6  IF level measured in serum at postpartum day 50 | Neonatal GEN exposure:  Postpartum day 2, 4 and 6  End of experiment at postpartum day 230 | In offspring tumour latency **↑**, tumour incidence and multiplicity **↓** | Lamartiniere et al. (1995) |
| **Chemically-induced tumour model**  **DMBA**  Tumour induction in offspring started on postpartum day 50 | **Rat**  Sprague-Dawley CD  Intact | GEN enriched **diet** | - 25 GEN - 250 GEN - Control diet soy-free | Not provided  IF level measured in serum, mammary glands and milk | Perinatal GEN exposure (via dams only):  From conception (2 weeks before breeding) to postpartum day 21 (weaning) (**for ~8 weeks****)  End of experiment at postpartum day 200 | In offspring tumour multiplicity **↓**.  GEN 250 µg/g diet group (50% reduction)  GEN 25 µg/g diet group (20% reduction) | Lamartiniere et al. (2002)  Fritz et al. (1998) |
| **Chemically-induced tumour model**  **DMBA**  Tumour induction in offspring started on postpartum day 50 | **Rat**  Sprague-Dawley CD  Intact | GEN enriched **diet** | - 250 GEN - Control diet soy-free | Not provided  IF level measured in serum | 1) Prenatal GEN exposure (via dams only):  During breeding and pregnancy of dams (**for ~3 weeks****)  End of experiment at postpartum day 230  2) Prepubertal GEN exposure (via dams only):  From parturition to postpartum day 21 (weaning) (**for ~3 weeks****)  End of experiment at postpartum day 180  3) Prepubertal (via dams only) following adult GEN exposure:  From parturition to postpartum day 21 (**for ~3 weeks****, via dams only) and from postpartum day 100 to 180 (**for ~11.4 weeks****)  End of experiment at postpartum day 180 | 1) Prenatal exposure:  In offspring mammary carcinogenesis **↔**  2) Prepubertal exposure:  In offspring tumour multiplicity **↓**  3) Prepubertal and adult exposure:  In offspring tumour multiplicity **↓** | Lamartiniere et al. (2002) |
| **Chemically-induced tumour model**  **DMBA**  Tumour induction started postnatal day 50 | **Rat**  Sprague-Dawley  Intact and ovariectomised (2 weeks post-carcinogen treatment) | IF enriched **diet**  (IFs contain: 49.7% GIN, 5.3% DIN, 34.5% glycitin) | - 100 IFs (sum of GIN, DIN, glycitin) - 500 IFs (sum of GIN, DIN, glycitin) - 1000 IFs (sum of GIN, DIN, glycitin) - Control diet soy-free | Not provided  IF blood level not measured | IF exposure:  Started 2 weeks + 1 day after tumour induction until end of experiment (**for ~22 weeks****)  End of experiment 24 weeks after tumour induction | Intact rats: Tumour incidence **↓**, tumour multiplicity **↓**, tumour latency **↑** (except 100 µg/g diet group: **↔**), tumour size **↔**  Ovariectomised rats: Tumour incidence **↓**, tumour multiplicity **↓**, tumour latency **↑**, tumour size **↔** | Ma et al. (2014) |
| **Chemically-induced tumour model**  **DMBA**  Tumour induction in offspring started on postnatal day 45 or 50 | **Rat**  Sprague-Dawley  Intact | GEN (or zearalenone) by **subcutaneous injection** | Diet is not described | 0.1, 0.5 or 1.5 mg/kg BW/day  IF blood level not measured | Prenatal GEN exposure (via dams only):  Between days 15-20 of pregnancy (**for ~6 days****)  End of experiment 18-20 weeks after carcinogen administration | In offspring of GEN groups:  Tumour latency **↔**, tumour size **↔**, tumour multiplicity **↔** Tumour incidence **↑** (except 0.5 mg/kg BW/day group: **↔**) | Hilakivi-Clarke et al. (1999a) |
| **Chemically-induced tumour model**  **DMBA**  Tumour induction started postnatal day 45 | **Rat**  Sprague-Dawley  Intact | GEN (or zearalenone) by **subcutaneous injection** | Diet is not described | 20 µg GEN/rat  (2 mg/kg BW (day 7) to 0.7 mg/kg BW (day 20))  IF blood level not measured | GEN exposure:  Postnatal day 7, 10, 14, 17 and 20  End of experiment 19 weeks after carcinogen administration | GEN group: Tumour incidence **↔**, tumour multiplicity **↓**, tumour growth rate **↓** | Hilakivi-Clarke et al. (1999b) |
| **Chemically-induced tumour model**  **DMBA**  Tumour induction started at 8 weeks of age | **Rat**  Sprague-Dawley  Intact | GEN, DAI, equol or tamoxifen by **orally administration** | Phytoestrogen-free diet | GEN: 0.73 mg/kg BW/day  DAI: 0.69 or 6.9 mg/kg BW/day  Equol: 0.65 mg/kg BW/day  IF blood level not measured | IF exposure:  From 8 weeks of age **for 3 weeks** (end of experiment) | DAI and equol group: Tumour growth and size **↓**  GEN and tamoxifen group: Tumour growth and size **↔** | Liu et al. (2012) |
| **Chemically-induced tumour model**  **DMBA**  Tumour induction started at 50 days of age | **Rat**  Sprague-Dawley  Intact | SPI enriched **diet** | - Low: 30 IFs - Medium: 400 IFs - High: 810 IFs - Control diet SP-free, but contained soybean oil (IF content not described) | IF dose is provided in detail (for each IF) in Tab. 2 of the publication  IF blood level not measured | IF exposure:  From day 36 to day 127 of age (end of experiment) (**for 13 weeks**) | Tumour latency **↔**, tumours/tumour bearing rat **↔**, tumour incidence **↓** | Appelt and Reicks (1999) |
| **Chemically-induced tumour model**  **DMBA + MPA**  Tumour induction started at 7 weeks of age (start of MPA administration) followed by DMBA administration started at 9 weeks of age | **Mouse**  ERα-KO and ERα-wildtype (mixed C57Bl/6J and 129SVJ background)  Intact | GEN enriched **diet** | - 1000 GEN - Control diet soy-free | ~100 mg/kg BW/day** at the end of experiment  IF blood level not measured | GEN exposure:  Start of exposure is not clearly described; definitely from 6 to 34 weeks of age (end of experiment) (**for ~28 weeks****) | *ERα-KO mice developed no tumours with GEN or control diet*  ERα-wildtype mice: Tumour size **↔**, tumour latency **↔**, tumour incidence **↔**, number of malignant tumours **↑** | Day et al. (2001) |
| **Chemically-induced tumour model**  **DMBA + MPA**  Tumour induction started at 6 weeks of age (start of MPA administration) followed by DMBA administration started at 7 weeks of age | **Mouse**  Heterozygous *Brca1^+/-^* (129Sv/C57BL/6 background) and wild-type (strain not clearly described)  Intact | GEN enriched **diet** | - 500 GEN - Control diet SP-free, but contained soybean oil (IF content not described) | Not provided  IF blood level not measured | Prepubertal GEN exposure:  From postnatal day 15 to postnatal day 30 (**for ~16 days****)  End of experiment: 20 weeks after last dose of DMBA administration (30 weeks of age**) | In Brca1^+/-^ and wild-type mice tumour incidence **↔** and **↓**, respectively | de Assis et al. (2011) |
| **Chemically-induced tumour model**  **MNU**  Tumour induction started at 7 weeks of age | **Rat**  CD/Crj Sprague-Dawley  Intact | Soybean, miso, or biochanin A enriched **diet** | - 2% or 10% powered soybeans in diet (IF content not described) - 10% miso in diet (IF content not described) - 10 or 50 biochanin A - Control diet (IF or soy content not described) | Not provided  IF level measured in plasma in a preliminary experiment | IF exposure:  Started at 7 weeks of age until end of experiment (**for ~18 weeks****)  End of experiment at 19 weeks after MNU administration | Tumour incidence **↔** (except biochanin A 50 µg/g diet group: **↓**)  Tumour multiplicity**↓** (except in 2% soybean diet group: **↔**) | Gotoh et al. (1998) |
| **Chemically-induced tumour model**  **MNU**  Tumour induction in offspring started at postnatal day 50 | **Rat**  Sprague-Dawley  Intact | SPI enriched **diet** | - 430 IFs (in sum, including 276 GEN and 132 DAI) in SPI enriched diet - Control diet soy-free | Not provided  IF blood level not measured | Prenatal (via dams only) following lifelong IF exposure:  From gestation day 4 to post-NMU day 115 (end of experiment) (**for ~26-27 weeks****) | In offspring tumour incidence **↓**, tumour latency **↑**, tumour multiplicity **↔**, tumour weight **↔** (tendency to higher tumour grade **↑**) | Simmen et al. (2005) |
| **Chemically-induced tumour model**  **MNU**  Tumour induction in offspring started at postnatal day 51 | **Rat**  Sprague-Dawley  Intact | SPI or GEN enriched **diet** | - SPI group: 394 IFs (in sum, including 216 GEN and 160 DAI) - GEN group: 250 GEN - Control diet soy-free | Not provided  IF blood level not measured | 1) Prenatal (via dams only) following lifelong SPI exposure:  From gestation day 4 to postnatal day 149 (end of experiment) (**for ~23-24 weeks****)  2) Prenatal SPI or GEN exposure (via dams only):  From gestation day 4 to delivery of pups (**for ~18 days****)  End of experiment at postnatal day 149 | 1) Lifelong exposure:  In offspring tumour incidence **↓**, tumour multiplicity **↔**  2) Prenatal exposure:  In offspring of SPI group: Tumour latency **↑**, tumour multiplicity **↔**, higher grade tumours **↓** (tendency)  In offspring of GEN group: No difference to control group | Su et al. (2007) |
| **Chemically-induced tumour model**  **MNU**  Tumour induction started at 45 days of age | **Rat**  Sprague-Dawley  Intact | GEN by **subcutaneous injection** | IF or soy content of diet not described | 1 mg/kg BW/day  IF blood level not measured | GEN exposure:  Started at 45 days of age **for 20 weeks** (end of experiment) | Tumour incidence **↔**, tumour latency **↔**, tumour multiplicity **↑**, tumour size **↑**, metastases **↑** | Kijkuokool et al. (2006) |
| **Chemically-induced tumour model**  **MNU**  Tumour induction started at 50 days of age | **Rat**  F-344  Intact | SPI or IF-depleted SPI enriched **diet** | Control diet (SP-free, but contained soybean oil) enriched with:   1. 20% SPI (212 GEN and 108 DAI**) 2. 10% SPI (106 GEN and 54 DAI**) 3. 20% IF-depleted SPI (14 GEN and 10 DAI**) 4. 10% IF-depleted SPI (7 GEN and 5 DAI**) 5. Nothing (pure control diet, IF content not described) | Estimated by authors [µg/day/rat]:   1. 2110 GEN and 1410 DAI 2. 1060 GEN and 707 DAI 3. 146 GEN and 126 DAI 4. 73 GEN and 63 DAI 5. 0 GEN and 0 DAI   IF level measured in urine | All rats received a standard diet (contained SP and soy oil) until 43 days of age  Experimental IF exposure:  Started at 43 days of age until approx. 18 weeks after MNU administration (end of experiment) (**experimental IF exposure for ~19 weeks****) | Tumour incidence **↔**, tumour latency **↔**, tumour multiplicity **↔**  Trend towards tumour inhibition in both soy groups (SPI and IF-depleted SPI) compared to control group, but not statistically significant | Cohen et al. (2000) |
| **Chemically-induced tumour model**  **EMS**  Tumour induction started 4 weeks after birth | **Rat**  Wistar King A  Intact | GEN or soy enriched **diet** | - 30 GEN - 1000 GEN - Soy-containing diet (no information about IF content) - Control diet IF-free | Not described  IF level measured in plasma (16 weeks of age ) | Within breeding perinatal exposure (via dams) to soy-containing diet until weaning (4 weeks after birth)  Experimental IF exposure:  Started after weaning period (4 weeks after birth) until appearance of tumour more than 5 mm in size (to age of 196 days maximum) (end of experiment) (**experimental IF exposure for ~24 weeks** maximum**) | GEN group: Tumour latency **↓** (but not significant)  Soy-containing diet group: Tumour latency **↑** (only significant for comparison to GEN-containing diets, but not for comparison to control diet) | Ono et al. (2012) |
| **Chemically-induced tumour model**  **E2**  Tumour induction in offspring started at postnatal day 45 | **Rat**  August-Copenhagen-Irish (ACI/SegHsd)  Intact | Soy extract enriched **diet** | - 476 IFs (in sum as aglycone equivalents, including 236 GEN, 195 DAI and 45 GLY) - Control diet IF-free | 54-30 mg/kg BW/day (sum of aglycone equivalents of GEN, DAI and Gly)  IF level measured in plasma | Prenatal (via dams only) following lifelong IF exposure:  From conception until postnatal day 285 (end of experiment) (**for ~43-44 weeks****) | In offspring tumour incidence **↓**, tumour multiplicity **↓**, tumour latency **↓** | Möller et al. (2016) |
| **Studies in genetically-induced tumour models** | | | | | | | |
| **Animal model** | **Animal**  **(female)** | **IF source and administration route** | **IF amount in diet**  **[ppm = µg/g diet]** | **IF dose/day;**  **IF blood level Y/N?** | **Treatment period and end of experiment** | **Effect of IF treatment compared to negative control groups regarding mammary carcinogenesis*** | **Ref.** |
| **Genetically-induced tumour model**  **Transgenic** | **Mouse**  MMTV-neu/ErbB-2  Intact  One full-term pregnancy during the first 3 months of life | GEN, DAI, or soy extract enriched **diet** | - 250 GEN - 250 DAI - 250 GEN aglycone equivalents in soy extract enriched diet (no details about other IFs) - Control diet SP-free, but contained soybean oil (IF content not described) | ~0.75 mg/day  (~31 mg/kg BW/day at end of study**)  IF blood level not measured | IF exposure:  Started at 7 weeks of age until postnatal day ~231-238 (end of experiment) (**for** **~26-27 weeks****) | Tumour latency **↑**, Tumour burden (size, multiplicity) **↔** at 34 weeks of age | Jin and MacDonald (2002) |
| **Genetically-induced tumour model**  **Transgenic**  Implantation of either placebo or tamoxifen pellet started at 8 weeks of age | **Mouse**  Wild-type erbB-2/neu    Intact | Soy meal-based **diet** or isolated IF enriched diet | - Soy meal-based diet: 491 IFs (including 214 GEN and 277 DAI) - IF-enriched diet (low): 211 IFs (including 137 GEN and 74 DAI) - IF-enriched diet (high): 491 IFs (including 214 GEN and 277 DAI) - Control diet IF-free | Not provided  IF blood level not measured | IF exposure:  Started at 4-5 weeks of age until 60 weeks of age (end of experiment) (**for ~55-56 weeks****) | With placebo implant: Tumour latency **↔** (no statistically significant differences between the four diet groups) | Liu et al. (2005) |
| **Genetically-induced tumour model**  **Transgenic**  Implantation of either placebo, tamoxifen or E2 pellet started at 8 weeks of age | **Mouse**  Wild-type erbB-2/neu  Intact | SP-based **diet** | - SP-based diet: 491 IFs (including 214 GEN and 277 DAI) - Control diet estrogen activity-free | Not provided  IF blood level not measured | IF exposure:  Started at 4-5 weeks of age until 60 weeks of age (end of experiment) (**for ~55-56 weeks****) | With placebo pellet: Tumour latency **↑**    With E2 pellet: Tumour latency **↑** | Yang et al. (2003) |
| **B. IF exposure started after tumour onset** | | | | | | | |
| **Studies in xenograft models** | | | | | | | |
| **Animal model** | **Animal**  **(female)** | **IF source and administration route** | **IF amount in diet**  **[ppm = µg/g diet]** | **IF dose/day;**  **IF blood level Y/N?** | **Treatment period and end of experiment** | **Effect of IF treatment compared to negative control groups regarding mammary carcinogenesis*** | **Ref.** |
| **Xenograft model**  **MCF-7 cells**  ER positive tumours | **Mouse**  Athymic nude  Ovariectomised (at 21 days of age) | GEN or GIN enriched **diet** | - 750 GEN aglycone equivalents in both enriched diets - Control diet described as IF-free (but contained soybean oil) | Not provided  IF level measured in plasma, but not shown in detail | For initial tumour growth mice were implanted with an E2 pellet (2 mg) before MCF-7 cells were injected. The E2 pellet was removed at start of the experimental exposure.  Experimental IF exposure:  Started when tumours reached an average tumour size of 40 mm² (~4 weeks), **for 11 weeks** (end of experiment) | *In negative control regression of tumours*  Both groups (GEN and GIN): Tumour growth **↑**, no statistically significant difference between both groups  After removal of GEN and GIN exposure, tumour size **↓** in both groups | Allred et al. (2001b) |
| **Xenograft model**  **MCF-7 cells**  ER positive tumours | **Mouse**  Athymic nude  Ovariectomised (at 21 days of age) | GEN or SPI (60% of IF in aglycone form) enriched **diet** | - GEN enriched diets:   - 15 GEN   - 150 GEN   - 300 GEN - SPI enriched diets:   - 15 GEN aglycone equivalents   - 150 GEN aglycone equivalents   - 300 GEN aglycone equivalents - Control diet soy-free | Not provided  IF blood level not measured | For initial tumour growth mice were implanted with an E2 pellet (2 mg) before MCF-7 cells were injected. The E2 pellet was removed at start of the experimental exposure.  Experimental IF exposure:  Started when tumours reached an average tumour size of 40 mm² (~4 weeks), **for 29 weeks** (end of experiment) | *In negative control regression of tumours*  150 and 300 µg/g groups (SPI and GEN, respectively): Tumour growth **↑** in dose-dependent manner (regression of tumour during first 10-12 weeks, afterwards tumour regrowth until end of experiment (week 29))  GEN 15 µg/g diet and SPI groups: Tumour growth **↓** (not different from negative control) | Allred et al. (2001a) |
| **Xenograft model**  **MCF-7 cells**  ER positive tumours | **Mouse**  Athymic nude  Ovariectomised (at 21 days of age) | **Diet** enriched with different IF sources (soy molasses, soy extract, GIN, mixed IF, soy flour plus mixed IF) | - In all IF enriched diets ~750 GEN aglycone equivalents - Complete IF content in enriched diets as sum of aglycone equivalents of GEN, DAI and GLY:**   - Soy molasses: 1482   - Soy extract: 1524   - GIN: 837   - mixed IF: 1210   - Soy flour plus mixed IF: 1455 - Control diet soy-free | Not provided  IF level measured in plasma, but not shown in detail | For initial tumour growth mice were implanted with an E2 pellet (2 mg) before MCF-7 cells were injected. The E2 pellet was removed at start of the experimental exposure.  Experimental IF exposure:  Started when tumours reached an average tumour size of 40 mm² (~4 weeks), **for 11 weeks** (end of experiment) | *In negative control regression of tumours*  Soy flour group: Tumour size **↔**  All other IF-treated groups: Tumour growth **↑** with different rates | Allred et al. (2004b)  Liu et al. (2015) |
| **Xenograft model**  **MCF-7 cells**  ER positive tumours | **Mouse**  Athymic BALB/c (nude)  Ovariectomised (at 21 days of age) | GEN enriched **diet** | - 125 GEN - 250 GEN - 500 GEN - 1000 GEN - Control diet soy-free | Not provided  IF level measured in plasma | For initial tumour growth mice were implanted with an E2 pellet (2 mg) before MCF-7 cells were injected. The E2 pellet was removed at start of the experimental exposure.  Experimental GEN exposure:  Started when tumours reached an average tumour size of 38 mm², **for 22 weeks** (end of experiment) | *In negative control regression of tumours*  GEN 125 µg/g diet group: Tumour size **↓** (not different from negative control)  GEN-treated groups (250-1000 µg/g diet): Dose-dependent tumour growth **↑** | Ju et al. (2001) |
| **Xenograft model**  **MCF-7 cells**  ER positive tumours | **Mouse**  Athymic BALB/c (nude)    Ovariectomised (at 21 days of age) | DAI or (R,S)-equol enriched **diet** | - 125 DAI - 250 DAI or (R,S)-equol - 500 DAI or (R,S)-equol - 1000 DAI or (R,S)-equol - Control diet soy-free | Not provided  IF level measured in plasma | For initial tumour growth mice were implanted with an E2 pellet (2 mg) before MCF-7 cells were injected. The E2 pellet was removed at start of the experimental exposure.  Experimental IF exposure:  Started when tumours reached an average tumour size of 35-37 mm², **for 21 weeks** (DAI) or **for 37 weeks** ((R,S)-equol) (end of experiment) | *In negative control regression of tumours*  All (R,S)-equol groups: Tumour size **↓** (not different from negative control)  All DAI groups: Tumour size **↓** (slower regression of tumours compared to negative control  In DAI 500 and 1000 µg/g diet group: slowly tumour growth after 18 weeks of regression) | Ju et al. (2006) |
| **Xenograft model**  **MCF-7 cells**  ER positive tumours | **Mouse**  Athymic BALB/c  Ovariectomised (at 21 days of age) | GEN or SPI enriched **diet** | - GEN enriched diets:   - 500 GEN   - 750 GEN - SPI enriched diet:   - 270 IFs (including 180 GEN, 72 DAI, 18 GLY) - Control diet described as GEN-free (but contained soybean oil) | Not provided  IF level measured in plasma | For initial tumour growth mice were implanted with an E2 implant before MCF-7 cells were injected. The E2 implant was removed at start of the experimental exposure.  Different experimental exposure scenarios started when tumours reached an average tumour size of ~40 mm².  Exposure scenarios (treatment/withdrawal):  GEN 500 (**23 weeks**/10 weeks)  GEN 750 (**15 weeks**/9 weeks)  SPI (**31 weeks**/9 weeks) | *In negative control regression of tumours*  Tumour growth **↑** with different rates  More aggressive and advanced growth phenotypes in tumours of GEN 500 µg/g diet group and SPI group than in GEN 750 µg/g diet group | Andrade et al. (2015) |
| **Xenograft model**  **MCF-7 cells**  ER positive tumours | **Mouse**  Athymic (Balb/c nu/nu)  Ovariectomised (before 4-5 weeks of age, exact date of ovariectomy is not described) | SPI, flaxseed or SPI plus flaxseed enriched **diet** | - SPI enriched diet (20% SPI):   - 626 IFs** (including 322 GEN, 262 DAI, 42 GLY)** - Flaxseed enriched diet (10% flaxseed) - SPI plus flaxseed enriched diet (20% SPI and 10% flaxseed):   - 626 IFs** (including 322 GEN, 262 DAI, 42 GLY)** - Control diet soy-free | Not provided  IF blood level not measured | For initial tumour growth mice were implanted with an E2 pellet (1.7 mg) before MCF-7 cells were injected. The E2 pellet was removed at start of the experimental exposure.  Experimental IF exposure:  Started when tumours reached an average tumour size of 35 mm² (~6 weeks), **for 25 weeks** (end of experiment) | *In negative control group and flaxseed group tumour regression over 25 weeks*  SPI group: Tumour growth **↑** (tumour regression during the first 10 weeks, stop of regression for 8 weeks and tumour regrowth steadily to end of experiment)  SPI plus flaxseed group: Tumour growth **↓** (similar to negative control) | Saarinen et al. (2006)  Power et al. (2008) |
| **Xenograft model**  **MCF-7 cells**  ER positive tumours | **Mouse**  Athymic (Balb/c nu/nu)  Ovariectomised (before 5-6 weeks of age, exact date of ovariectomy is not described) | GEN, lignans or GEN plus lignans by **subcutaneous injection** | Soy-free diet | GEN group: 10 mg GEN/kg BW/day  GEN plus lignans: 3.33 mg GEN/kg BW/day  IF blood level not measured | For initial tumour growth mice were implanted with an E2 pellet (1.7 mg) before MCF-7 cells were injected. The E2 pellet was removed at start of the experimental exposure.  Experimental GEN exposure:  Started when tumours reached an average tumour size of 35 mm², **for 22 weeks** (end of experiment) | *In negative control regression of tumours*  GEN group:  Tumour regression only in the first 4 weeks; from week 4-22 tumour size remained; tumour size **↑** at end of experiment compared to negative control  GEN plus lignans group: Tumour regression over whole treatment period; tumour size **↔** at end of experiment compared to negative control | Power et al. (2006) |
| **Xenograft model**  **MCF-7 cells**  ER positive tumours | **Mouse**  Athymic nude (nu/nu-BALB/c)  Ovariectomised (before 8 weeks of age, exact date of ovariectomy is not described) | GEN or DAI by **orally administration** | Phytoestrogen-free diet | GEN: 7.3 mg/kg BW/day  DAI: 6.9 mg/kg BW/day  IF blood level not measured | For initial tumour growth mice were implanted with an E2 pellet (0.72 mg) before MCF-7 cells were injected. The E2 pellet remained during the whole study.  Experimental IF exposure:  Started when tumours reached an average tumour size of ~6 mm diameter (28 mm² **), **for 4 weeks** (end of experiment) | *In control group progression of tumours (E2 pellet was implanted in all groups during the whole study)*  GEN group: Tumour growth **↔** (compared to control)  DAI group: Tumour growth **↓** (compared to control) | Liu et al. (2012) |
| **Xenograft model**  **MCF-7 cells**  ER positive tumours | **Mouse**  SCID  Intact | Soy extract or purified IFs enriched **diet**  Soy extract contained (as aglycone equivalents) 50.8% GEN, 40.5% DAI, and 8.7% GLY  Purified IFs contained (as aglycone equivalents) 90.1% GEN, 9.1% DAI, and 0.8% GLY | - Soy extract enriched diet:   - 0.1% soy extract: 519 IFs**   - 0.5% soy extract: 2595 IFs** - Purified IFs enriched diet:   - 0.028% purified IFs: 280 IFs**   - 0.14% purified IFs: 1400 IFs** - Control diet SP-free, but contained soybean oil (IF content not described) | Not provided  IF blood level not measured | IF exposure:  Started 2 weeks before implantation of E2 pellet and MCF-7 cells (age of 6-9 weeks**), end of experiment 8 weeks after tumour cell implantation (IF exposure **for 10 weeks****) | *In control group progression of tumours (E2 pellet was implanted in all groups during the whole study)*  Soy extract and purified IFs group: Tumour growth **↓** and tumour weight **↓** (both dose-dependent) compared to control | Zhou et al. (2004) |
| **Xenograft model**  **MCF-7 cells**  ER positive tumours  **MDA-MB-231 cells**  ER negative tumours | **Mouse**  HSD: Athymic Nude-nu  For MCF-7 protocol: Ovariectomised (6-7 weeks of age**)  For MDA-MB-231 protocol: Intact | Soy extract by **oral gavage** | Phytoestrogen-free diet | 50 mg soy extract/kg BW/5 days per week: 8.4 mg IFs (GIN+DIN)/kg BW/5 days per week  100 mg soy extract/kg BW/5 days per week: 16.8 mg IFs (GIN+DIN)/kg BW/5 days per week  IF level measured in plasma in a preliminary experiment | MCF-7 protocol:  IF exposure started 1 week after ovariectomy, i.e. the day before tumour cell inoculation, **for 5 weeks** (end of experiment) (with or without E2 pellet (0.18 mg) implanted at start of IF exposure)  MDA-MB-231 protocol:  IF exposure started with the day of cell inoculation **for ~40 days (read out of diagram)** (end of experiment) | MCF-7 protocol with E2 pellet:  *In control group (with E2 pellet, implanted during the whole study) progression of tumours*  Both soy extract groups (with E2 pellet): Tumour growth **↔** (compared to control group with E2 pellet)  MCF-7 protocol without E2 pellet:  *In control group (without E2 pellet) regression of tumours*  Both soy extract groups (without E2 pellet): Tumour growth **↔** (compared to control group without E2 pellet)  MDA-MB-231experiment:  *In control group progression of tumours*  Both soy extract groups: Tumour growth **↔** | Gallo et al. (2006) |
| **Xenograft model**  **MCF-7 cells**  ER positive tumours  **MDA-MB-231 cells**  ER negative tumours | **Mouse**  Nude (nu/nu mutants on a BALB/c background)  Intact | GEN by **subcutaneous injection** | Diet is not described | 0.1, 0.2, 0.5 GEN mg/kg BW/every other day  IF blood level not measured | GEN exposure:  Started 6 weeks after inoculation with tumour cells **for 2 weeks** (end of experiment)  In the experiment with MCF-7 cells, mice additionally received an E2 pellet (0.72 mg) 3 days before injection of tumour cells. The E2 pellet remained during the whole study. | *In control group (both, in MCF-7 and MDA-MB-231 model) progression of tumours*  GEN group (both, in MCF-7 and MDA-MB-231 model): Tumour growth **↓** (compared to corresponding control group) in a dose-dependent manner (only significant in 0.5 mg dose group) | Shao et al. (1998) |
| **Xenograft model**  **MCF-7-E10 cells**  ER positive tumours | **Mouse**  Athymic BALB/c (nude)    Ovariectomised (4 weeks of age) | GEN, *S*-equol or fermented soy germ enriched **diet** | - 250 or 500 GEN - 250 or 500 *S*-equol - 250 or 500 IFs in fermented soy germ group (sum of aglycone equivalents of DAI, GEN and GLY) - Control diet soy-free | Not provided  IF blood level not measured | For initial tumour growth mice were implanted with an E2 pellet (2 mg) one week before MCF-7-E10 cells were injected (6 weeks of age). The E2 pellet was removed at start of the experimental exposure.  Experimental IF exposure:  Started when tumours reached an average tumour size of 34 mm², **for 25 weeks** (end of experiment) | *In negative control initially regression of tumour size. After 10 weeks tumours slightly started to increase in all groups.*  Tumour growth **↔** (no significant difference between negative control and treatment groups)  In GEN 250 µg/g diet and GEN 500 µg/g diet group tumour growth was slightly greater than in the negative control group (not significant and no concentration dependence). | Onoda et al. (2011) |
| **Xenograft model**  **MDA-MB-231 cells**  ER negative tumours | **Mouse**  BALB/cA Jcl-nu athymic  Intact | GEN combined polysaccharide (GCP^TM^, functional food supplement) enriched **diet** | - Control diet (SP and soybean oil based) enriched with 1% GCP^TM^ (including 1160 GEN, 285 DAI and 135 GLY)** - Control diet SP and soybean oil based (IF content not described) | 162.4 ± 10.8 mg GEN/kg BW/day  GEN level measured in tumour tissues (no GEN detected in control group) | IF exposure:  From day of tumour cell injection (6 weeks of age) to end of experiment (**for ~ 4 weeks****); end of experiment at 28th day after tumour inoculation | *In control group progression of tumours*  Tumour growth **↓**, tumour weight **↓** | Yuan et al. (2003) |
| **Xenograft model**  **Aggressive bone metastatic variant of MDA-MB-435**  ER negative tumours | **Mouse**  Athymic nu/nu  Intact | GEN, DAI or a mixture of GEN, DAI and GLY by **oral gavage** | Phytoestrogen-free diet | - GEN: 10 mg/kg BW 3x per week - DAI: 10 mg/kg BW 3x per week - GEN 10, DAI 9 and GLY 1 mg/kg BW 3x per week   IF blood level not measured | IF exposure:  Started 1 week after tumour cell injection **for 78 days** (end of experiment) | *In negative control progression of tumours*  GEN group: Mammary tumour growth **↓**, lung, heart and kidney metastases **↔**, bone and liver metastases **↓**  DAI group: Tumour growth **↑**, lung and heart metastases **↑**, bone, liver and kidney metastases **↔**  GEN+DAI+GLY group: Tumour growth **↔**, lung, heart, bone, liver and kidney metastases **↑** | Martinez-Montemayor et al. (2010) |
| **Studies in allograft models** | | | | | | | |
| **Animal model** | **Animal**  **(female)** | **IF source and administration route** | **IF amount in diet**  **[ppm = µg/g diet]** | **IF dose/day;**  **IF blood level Y/N?** | **Treatment period and end of experiment** | **Effect of IF treatment compared to negative control groups regarding mammary carcinogenesis*** | **Ref.** |
| **Allograft model**  **Mouse F3II-cells**  ER positive tumours | **Mouse**  Balb/c  Intact | GEN, DAI or soy extract enriched **diet** | - GEN enriched diets:   - 250   - 500   - 750 - DAI enriched diets:   - 188   - 376   - 564 - Soy extract enriched diets:   - 0.2% soy extract: 250 GEN and 188 DAI**   - 0.4% soy extract: 500 GEN and 376 DAI**   - 0.6% soy extract: 750 GEN and 564 DAI** - Control diet SP-free, but contained soybean oil (IF content not described) | Not provided  GEN level measured in plasma in a preliminary experiment | IF exposure:  Started 5 days prior to injection with F3II cells until 21-25 days after cell inoculation (end of experiment) (**for ~4 weeks****) | Soy extract groups (0.2% and 0.6%): Tumour weight **↓** (0.4% group: reduced, but not significant)  GEN 750 µg/g diet group: Tumour weight **↓**  GEN groups (250 and 500 µg/g diet) and DAI groups (188, 376, and 564 µg/g diet): Tumour weight **↔** | Hewitt and Singletary (2003) |
| **Allograft model**  **LM3 mouse mammary tumour cells**  ER negative tumours | **Mouse**  Balb/c  Intact | Non-fermented soy product or fermented soy product or fermented soy product enriched with IFs. Administration by **oral gavage** | Diet contained IFs (810 ± 10 µg/g diet) | Non-fermented and fermented soy product: IFs 0.05 mg/day (~2.0-2.8 mg/kg BW/day**)  Fermented soy product enriched with IFs: 0.09 mg/day (~3.6-5.0 mg/kg BW/day**)  IF blood level not measured | IF exposure:  Started 10 days before tumour cell injection and thereafter for 30 days (end of experiment) (**for ~5-6 weeks****) | Tumour size **↓** (fermented soy product < non-fermented soy product = fermented soy product enriched with IFs < control) | Kinouchi et al. (2012) |
| **Studies in chemically-induced tumour models** | | | | | | | |
| **Animal model** | **Animal**  **(female)** | **IF source and administration route** | **IF amount in diet**  **[ppm = µg/g diet]** | **IF dose/day;**  **IF blood level Y/N?** | **Treatment period and end of experiment** | **Effect of IF treatment compared to negative control groups regarding mammary carcinogenesis*** | **Ref.** |
| **Chemically-induced tumour model**  **MNU**  Tumour induction started 21 days of age | **Rat**  Sprague-Dawley  Ovariectomised (after tumour development, 6 weeks after carcinogen exposure) | GEN enriched **diet** | - 750 Gen - Control diet soy-free | Not provided  GEN level measured in plasma | GEN exposure:  Started after tumour development (6 weeks after carcinogen exposure) for 90 days (end of experiment) (**for ~12-13 weeks****) | *After ovariectomy regression of tumours in all groups (also in negative control)*  Weight of estrogen-dependent adenocarcinomas **↑** | Allred et al. (2004a) |

##### I.B Effects on the female breast: Clinical intervention studies in healthy women

Hooper et al. (2010) considered data obtained from eight randomized controlled trials (RCTs) (first column, Table C). The data of these RCTs were published in 18 full-text publications, one published manuscript and an abstract (second column, Table C). Fritz et al. (2013) included some of the studies that were already considered by Hooper et al. (2010) and one that was not analysed within that meta-analysis due to the publication date (third column, Table C).

**Table C:** Cohorts and studies that were considered within the meta-analyses of Hooper et al. (2010) and Fritz et al. (2013)

| **RCTs considered in Hooper et al. (2010)** | **Publications considered**  **in Hooper et al. (2010)** | **Publications considered**  **in Fritz et al. (2013)** |
| --- | --- | --- |
| Atkinson (2004) | Atkinson et al. (2004) | Atkinson et al. (2004) |
|  | Kataoka et al. (2008) | Kataoka et al. (2008) |
| Marini (2008) | Marini et al. (2007) |  |
|  | Marini et al. (2008) | Marini et al. (2008) |
|  | D'Anna et al. (2009) |  |
|  | Atteritano et al. (2009) |  |
| Maskarinec (2002) | Maskarinec et al. (2002) |  |
|  | Maskarinec et al. (2003) | Maskarinec et al. (2003) |
| Maskarinec (2004) | Maskarinec et al. (2004a) |  |
|  | Maskarinec et al. (2004b) | Maskarinec et al. (2004b) |
| Maskarinec (2009; OPUS) | Maskarinec et al. (2009b) | Maskarinec et al. (2009b) |
|  |  | Steinberg et al. (2011) |
| Powles (2008) | Powles et al. (2008) | Powles et al. (2008) |
| Tice (2009; PREVENT) | Tice (2005) |  |
|  | Tice et al. (2005) |  |
| Verheus (2008; Finesse) | Kok et al. (2004) |  |
|  | Kok et al. (2005b) |  |
|  | Kok et al. (2005a) |  |
|  | Kreijkamp-Kaspers et al. (2005) |  |
|  | Kreijkamp-Kaspers et al. (2004) |  |
|  | Verheus et al. (2008) |  |

**Table D:** Statistical results and subgrouping of the meta-analysis by Hooper et al. (2010)

| **Factor** | **Subgroup** | **Number of studies** | **Number of participants** | **Mean difference (95% CI)** | **p-value for heterogeneity, I^2^***** |
| --- | --- | --- | --- | --- | --- |
| **Main analysis** | Overall analysis (no subgroup) | 7 | 1,149 | 0.69 (–0.78 to 2.17) | 0.14, 36% |
| **Menopausal status*** | Premenopausal | 5 | 519 | 1.83 (0.25 to 3.40) | 0.85, 0% |
|  | Postmenopausal | 4 | 592 | –1.10 (–3.22 to 1.03) | 0.23, 30% |
|  | Perimenopausal | 1 | 16 | –0.37 (–6.12 to 5.38) | - |
| **Duration**** | 6 to <18 months | 7 | 1,014 | –0.20 (–1.29 to 0.88) | 0.33, 13% |
|  | 18 to <30 months | 4 | 846 | 0.08 (–1.34 to 1.51) | 0.65, 0% |
|  | 30 + months | 2 | 241 | 3.22 (–0.18 to 6.63) | 0.62, 0% |

CI, confidence intervals

*Since the menopausal status of some study participants of the Atkinson RCT is not known, the total number of participants is smaller, when subgrouped by menopausal status, than in the overall analysis.

**In some trials, data were measured more than once or using more than one technique, so that numbers do not add up to the total number of study participants.

***Heterogeneity was assessed using Cochran´s test and the I^2^ test and assumed to be present when I^2^ > 50% (Hooper et al. 2010).

##### I.C Effects on the female breast: Observational studies

In Table 4 (chapter 5.1.3), the results of seven meta-analyses examining the association between dietary intake of isoflavones or soy proteins and the incidence of breast cancer in humans are discussed. The statistical analysis is additionally indicated in the following table.

**Table E:** Meta-analyses on the association between soy food or isoflavone intake and breast cancer incidence

| **Reference** | **Outcome** | | |
| --- | --- | --- | --- |
|  | **All women combined**  **(prem. and postm.)** | **Western population**  **(prem. and postm.)** | **Asian population**  **(prem. and postm.)** |
| **Trock et al. (2006)** | All: ↓^1^  prem.: ↓^2^ > postm.: ↓^3^ | All: no association^4^  prem.: n.d.  postm.: n.d. | All: no association^5^  prem.: n.d.  postm.: n.d. |
| **Qin et al. (2006)** | All: ↓^6^  prem.: ↓^7^ < postm.: ↓^8^ | All: n.d.  prem.: n.d.  postm.: n.d. | All***:**↓^9^  prem.: n.d.  postm.: n.d. |
| **Wu et al. (2008)** | All: n.d.  prem.: n.d.  postm.: n.d. | All: no association^10^  prem.: n.d.  postm.: n.d. | All*****: ↓^11^  prem.: ↓^12^  postm.: ↓^13^ |
| **Dong and Qin (2011)** | All: ↓^14^  prem.: no association^15^  postm.: ↓^16^ | All: no association^17^  prem.: n.d.  postm.: n.d. | All: ↓^18^  prem.: n.d.  postm.: n.d. |
| **Xie et al. (2013)** | All: n.d.  prem.: n.d.  postm.: n.d. | All: no association^19^  prem.: no association^20^  postm.: no association^21^ | All: ↓^22^  prem.: ↓^23^ < postm.: ↓^24^ |
| **Chen et al. (2014)** | All: n.d.  prem.: ↓^25^  postm.: ↓^26^ | All: n.d.  prem.: no association^27^ postm.: ↓^28^ | All: n.d.  prem.: ↓^29^ = postm.: ↓^30^ |
| **Wu et al. (2015)** | All: n.d.  prem.: n.d.  postm.: n.d. | All: n.d.  prem.: n.d.  postm.: n.d. | All: ↓^31^  prem.: n.d.  postm.: n.d. |

Prem., premenopausal; postm., postmenopausal; n.d., no data; ↓, increasing soy or isoflavone intake significantly decreased the risk of developing breast cancer; * one of the included studies is with Asian-Americans

Statistics as stated by the authors:

^1^ = OR=0.86, 95% CI=0.75–­­0.99

^2^ = OR=0.70, 95% CI=0.58–0.85

^3^ = OR=0.77, 95% CI=0.60–0.98

^4^ = OR=0.84 95% CI=0.70–1.00

^5^ = OR=0.89, 95% CI=0.71–1.12

^6^ = RR=0.75, 95% CI=0.59–0.95

^7^ = RR=0.64, 95% CI=0.53–0.77

^8^ = RR=0.73, 95% CI=0.58–0.93

^9^ = RR=0.71, 95% CI=0.52–0.93

^10^ = OR=1.04, 95% CI=0.97–1.11

^11^ = OR=0.73, 95% CI=0.61–0.89

^12^ = OR=0.65, 95% CI=0.50–0.85

^13^ = OR=0.63, 95% CI=0.46–0.85

^14^ = RR=0.89, 95% CI=0.79-0.99

^15^ = RR=0.90, 95% CI=0.64–1.15

^16^ = RR=0.78, 95% CI=0.63–0.93

^17^ = RR=0.97, 95% CI=0.87–1.06

^18^ = RR=0.76, 95% CI=0.65–0.86

^19^ = RR/OR=0.98, 95% CI=0.87–1.11

^20^ = RR/OR=1.00, 95% CI=0.98–1.02

^21^ = RR/OR=0.99, 95% CI=0.87–1.12

^22^ = RR/OR=0.68, 95% CI=0.52–0.89

^23^ = RR/OR=0.63, 95% CI=0.50–0.80

^24^ = RR/OR=0.46, 95% CI=0.28–0.78

^25^ = OR=0.74, 95% CI=0.64–0.85

^26^ = OR=0.75, 95% CI=0.63–0.86

^27^ = OR=0.90, 95% CI=0.77–1.04

^28^ = OR=0.92, 95% CI=0.83–1.00; not statistically significant trend

^29^ = OR=0.59, 95% CI=0.48–0.69

^30^ = OR=0.59, 95% CI=0.44–0.74

^31^ = OR=0.68, 95% CI=0.50–0.93

In Table 8 (chapter 5.1.3), the results of two meta-analyses examining the association between dietary intake of isoflavones or soy proteins and the recurrence of breast cancer in humans are discussed. The statistical analysis is additionally indicated in the following table.

Table G: Meta-analyses of observational studies on the association between soy intake and breast cancer recurrence

| **Reference** | **Outcome** | | |
| --- | --- | --- | --- |
|  | **all women combined**  **(prem. and postm.)** | **Western population**  **(prem. and postm.)** | **Asian population**  **(prem. and postm.)** |
| **Dong and Qin (2011)** | All: ↓^1^  prem.: no association^2^  postm.: ↓^3^ | All: n.d.  prem.: n.d.  postm.: n.d. | All: n.d.  prem.: n.d.  postm.: n.d. |
| **Chi et al. (2013)** | All: ↓^4^  prem.: no association^5^  postm.: ↓^6^ | All: n.d.  prem.: n.d.  postm.: n.d. | All: n.d.  prem.: n.d.  postm.: n.d. |

Prem., premenopausal; postm., postmenopausal; ↓, increasing soy or isoflavone intake significantly decreases the risk of breast cancer recurrence

Statistics as stated by the authors:

^1^ = RR=0.84, 95% CI=0.70–0.99

^2^ = RR=0.88, 95% CI=0.66–1.10

^3^ = RR=0.78, 95% CI=0.58–0.97

^4^ = HR=0.74, 95% CI=0.64–0.85

^5^ = HR=0.91, 95%CI=0.72–1.14

^6^ = HR=0.67, 95%CI=0.56–0.80

##### I.D Effects on the thyroid hormone system: RCTs investigating the effects in postmenopausal and ovariectomised women

In the last years some RCTs analysing the impact of isoflavones on postmenopausal or ovariectomised women were published (chapter 5.2.3; Table 9). In the following table the statistics are additionally indicated.

Table H: Clinical intervention studies investigating a potential association between isoflavone or soy intake and thyroid gland dysfunction in postmenopausal or ovariectomised women

| **Reference** | **Outcome** |
| --- | --- |
|  |  |
| **(Duncan et al. 1999)^✰^** | - No effect on:   total T4, fT4, total T3, fT3 and TSH   - **Effect on:**   TBG↓^1^ |

| **Persky et al. (2002)^✰^** | - **Effect on** (3 and 6 mo., 56 mg):   T4↑^2^, FTI↑^3^   - **Effect on** (3 and 6 mo., 90 mg):   TSH↑^4^   - **Effect on** (6 mo., 90 mg):   T3↑^5^ |
| --- | --- |
| **Bruce et al. (2003)^✰^** | - No effect on:   TSH, T3 and T4 serum conc./levels |
| **Ryan-Borchers et al. (2008)** | - No effect on:   TSH |
| **Khaodhiar et al. (2008)** | - No effect on:   TSH, Tg, fT3, fT4, total T3 and total T4 |
| **Pop et al. (2008)** | - No effects on:   T4, TSH, T3 uptake, FTI |
| **Bitto et al. (2010)** | - No effect on:   THRα, THRβ, RARα and RARβ mRNA levels in peripheral blood monocytes,  TSH, fT3, fT4, TPO and TG serum conc./levels, anti-TMA antibody serum conc./level |
| **Levis et al. (2011)** | - No effect on:   serum conc./level of TSH and anti-TPO antibody |
| **Steinberg et al. (2011)** | - No effect on:   TSH serum level, fT4 serum level |
| **Mittal et al. (2011)** | - No effects on:   TSH, fT4 and TBG serum levels, anti-TPO antibody serum level   - **Effect on**   fT3↓^6^ |
| **Alekel et al. (2015)** | - No effect on:   TSH serum conc./ level, fT4 serum conc./level |
| **Sathyapalan et al. (2017)** | - No effect on:   fT3   - **Effect on**   TSH↑^7^, fT4↑^8^ |

Perim.; perimenopausal; postm., postmenopausal; mo., months; fT3/T4, free T3/T4; FTI, free thyroxine index; no effect, p > 0.05; ✰, studies in which the examination of thyroid function was the primary endpoint; ↓, values decreased compared to controls; ↑ values increased compared to controls

Statistics as stated by the authors:

^1^ = p=0.03

^2^ = p=0.02

^3^ = p=0.03

^4^ = p=0.01

^5^ = p=0.04

^6^ = p=0.02

^7^ = p=<0.01

^8^ = p=<0.01

1. Influence of isoflavones on other endpoints in healthy women

To date the discussion predominantly focuses on the biological effects of isoflavones that are induced by ER-mediated mechanisms, but numerous ER-independent effects have also been described (Andres et al. 2011; Gallo et al. 2005; Hwang et al. 2006; Ko 2014; Kuiper et al. 1998; Setchell and Cassidy 1999). For example, isoflavones may interfere with estrogen metabolising enzymes, which may in turn result in an alteration of the concentration of circulating estrogens. A meta-analysis of 47 clinical trials by Hooper et al. (2009) showed that soy or isoflavone intake in the form of isoflavone extract, soy protein isolate, whole soy or soy foods (25-200 mg isoflavones/day) did not significantly affect circulating levels of E2, estrone, or sex hormone binding globulin (SHBG) in pre- and postmenopausal women, although amongst premenopausal women there was a significant decrease of 20 % in the level of luteinizing hormone (LH) and follicle-stimulating hormone (FSH). These findings are in agreement with those reported in a recent systematic review by Fritz et al. (2013), who did not detect any changes in the circulating E2 levels in 18 randomised clinical trials. This study included 4 recent and 2 older clinical trials that were not considered by Hooper et al. (2009) in their meta-analysis. Moreover, no significant effects of soy isoflavones (~50 mg isoflavones/day) on the levels of E2 and estrone sulphate (E1S) in nipple aspirate fluid (NAF) and serum of premenopausal women were reported (Maskarinec et al. 2011b). In a study by Khan et al. (2012), no isoflavone-related effects on hormone (E2 and estrone) and protein levels as well as on the median plasma concentration of FSH and SHBG and the E2:SBHG ratio were observed in the NAF of pre- and postmenopausal women following intervention (235 mg of mixed isoflavones/day for six months).

2-Hydroxy (2-OH) metabolites of estrone appear to inhibit breast cell proliferation, while 16α-OH estrone metabolites have been suggested to promote different steps of tumorigenesis (Yager and Liehr 1996; Zhu and Conney 1998). Therefore, an increase in the urinary 2-OH estrone to 16α-OH estrone ratio has been postulated to represent a cancer protective shift in estrogen metabolism (Morimoto et al. 2012). The impact of soy isoflavone consumption on the estrogen metabolism was investigated in several intervention studies, and these reported inconsistent results. In some of them, a significant increase in the urinary 2-OH estrone:16α-OH estrone ratio was observed in pre- as well as in postmenopausal women (Lu et al. 2000; Morimoto et al. 2012; Nettleton et al. 2005; Xu et al. 1998; Xu et al. 2000), while in other studies no such effect was described in premenopausal women after soy intervention (Brown et al. 2002; Martini et al. 1999; Maskarinec et al. 2012).

The NAF is in constant contact with the ductal epithelium, the site of development of most breast cancer types (Maskarinec et al. 2011b). Thus, NAF has been used to detect changes in the expression of cellular and non-cellular markers of breast cancer risk associated with soy intake (Maskarinec et al. 2011b). In addition, the volume of NAF is regarded as a marker of breast tissue proliferative activity (Fritz et al. 2013) and women producing NAF are thought to be at a higher risk of developing breast cancer (Maskarinec et al. 2013). Nevertheless, the ability to produce NAF at all or the volume of aspirated secretion may also depend on factors such age, ethnicity and onset of menarche, among others (Petrakis 1993)). A study by Maskarinec et al. (2008) indicated that isoflavones are present in the breast fluid and may directly act on the breast tissue. In a cross-over study in premenopausal women, the ingestion of an isoflavone supplement (genistein dose: 38 mg/day) for six months was found to increase the NAF volume (Petrakis et al. 1996). In contrast, in a randomised cross-over study in premenopausal women, following an administration of a high soy diet (two daily servings of soy food, 50 mg of isoflavones) for six months, no differences in NAF volume were detected (Maskarinec et al. 2011a).

The tumour suppressor genes breast cancer 1 and 2 (BRCA1 and BRCA2) are involved in the maintenance of the genomic integrity and transcriptional regulation of breast cells (Marini et al. 2008; Welcsh and King 2001). In a randomised, double-blind, placebo-controlled study involving 138 postmenopausal women that consumed 54 mg of genistein aglycone daily for three years BRCA 1 and 2 mRNA levels in the blood remained unaltered (Marini et al. 2008).

In a randomised, double-blind, placebo-controlled intervention study in pre- and postmenopausal women (Khan et al. 2012) a significant increase in the level of expression of 14 out of 28 genes involved in estrogen response or associated with breast epithelial atypia was determined in the soy group (235 mg of isoflavones/day; n=49), but not in the control group (n=49).

Data presented by Pudenz et al. (2014) suggest that epigenetic gene regulation, such as DNA methylation, histone tail modifications and non-coding RNAs, represent a potential novel target of isoflavones. Alterations in DNA methylation due to an early exposure to isoflavones might influence breast cancer risk later on (Qin et al. 2009). However, studies addressing epigenetic changes are scarce. A randomised, double-blind trial in premenopausal women (n=34) that ingested 40 or 140 mg of isoflavones daily throughout one menstrual cycle suggested that isoflavone intake might induce dose-specific changes in the methylation pattern of cancer-related genes (retinoic acid receptor-β2 [RARβ2] and cyclin D2 [CCDN2]). RARβ2 and CCND2 methylation was significantly reduced in blood samples of women with low circulating levels of genistein and were increased in women with high circulating levels of genistein (Qin et al. 2009).

Chronic inflammation is regarded as a potential risk factor for breast cancer. Intervention studies determining the effects of soy isoflavones on markers of chronic inflammation, e.g. C-reactive protein (CRP), interleukin-6, leptin or the anti-inflammatory adiponectin, showed conflicting results. Several studies did not observe any effect of soy isoflavones on these markers in premenopausal women (Maskarinec et al. 2009a) or in postmenopausal women (e.g. (D'Anna et al. 2005; Greany et al. 2008; Nikander et al. 2003; Ryan-Borchers et al. 2006; Tormala et al. 2008), while others described a decrease of CRP levels (Hall et al. 2005; Nasca et al. 2008; Yildiz et al. 2005) or an increase of adiponectin levels following soy isoflavone consumption in postmenopausal women (Charles et al. 2009).

Growth factors play an important role in cell regulation and tumorigenesis. Insulin-like growth factor (IGF)-1 is a mitogenic and antiapoptotic peptide hormone. Binding of IGF-1 to IGF-binding protein (IGFBP)-3 regulates the effects of IGF-1 (McLaughlin et al. 2011). Increased levels of both IGF-1 and IGFBP-3 have been associated with an enhanced breast cancer risk in animal and human studies (Hankinson et al. 1998; Renehan et al. 2004; Toniolo et al. 2000). Several intervention studies investigated the effect of soy or isoflavone intake on IGF-1 and IGFBP-3 blood levels. Results showed that the blood levels of IGF-1 and IGFBP-3 significantly increased after consumption of soy, soy protein or an isoflavone supplement in different population groups (pre/postmenopausal women, healthy men, cancer patients; Arjmandi et al. 2003; Dewell et al. 2007; Khalil et al. 2002; Maskarinec et al. 2005; McLaughlin et al. 2011; Woodside et al. 2006).

Nitrosative stress, which is associated with nitric oxide (NO) production, has been linked to the development of cancer at different sites including breast tissue (Pervin et al. 2010; Roberts et al. 2009; Sen et al. 2012). Isoprostane formation is directly associated with nitrosative stress caused by NO synthesis *in vivo* and is therefore considered as a marker for such a process (Marnett 2000; Tanaka et al. 2007). Several intervention studies investigating the effect of soy food or isoflavone consumption on urinary F(2)-isoprostane concentrations in pre- or postmenopausal women reported inconsistent results. In a 6-month intervention study, slightly higher isoprostane levels were observed with a high soy diet (approximately 50 mg isoflavones/day) than with a low soy diet (less than 75 mg isoflavone/week) (Sen et al. 2012). Other studies detected no difference in the isoprostane levels in urine (Djuric et al. 2001; Nhan et al. 2005; Ryan-Borchers et al. 2006) or plasma (Djuric et al. 2001). In contrast, an intervention study observed a significant decrease in the plasma concentration of F(2)-isoprostane after a high soy intake (56 mg isoflavones/day) versus a low soy intake (< 2 mg isoflavones/day) (Wiseman et al. 2000).

In summary, clinical studies addressing the influence of soy, soy protein or isolated isoflavones on other endpoints than mammographic density or proliferation markers, e.g. on estrogen metabolism, DNA methylation, genetic markers of breast cancer risk, growth factors or markers of nitrosative stress and inflammation, described conflicting results.

**References**

Alekel DL, Genschel U, Koehler KJ, et al. (2015) Soy Isoflavones for Reducing Bone Loss Study: effects of a 3-year trial on hormones, adverse events, and endometrial thickness in postmenopausal women. Menopause 22(2): 185-197

Allred CD, Allred KF, Ju YH, et al. (2004a) Dietary genistein results in larger MNU-induced, estrogen-dependent mammary tumors following ovariectomy of Sprague-Dawley rats. Carcinogenesis 25(2): 211-218

Allred CD, Allred KF, Ju YH, Goeppinger TS, Doerge DR, Helferich WG (2004b) Soy processing influences growth of estrogen-dependent breast cancer tumors. Carcinogenesis 25(9): 1649-1657

Allred CD, Allred KF, Ju YH, Virant SM, Helferich WG (2001a) Soy diets containing varying amounts of genistein stimulate growth of estrogen-dependent (MCF-7) tumors in a dose-dependent manner. Cancer Res 61(13): 5045-5050

Allred CD, Ju YH, Allred KF, Chang J, Helferich WG (2001b) Dietary genistin stimulates growth of estrogen-dependent breast cancer tumors similar to that observed with genistein. Carcinogenesis 22(10): 1667-1673

Andrade JE, Ju YH, Baker C, Doerge DR, Helferich WG (2015) Long-term exposure to dietary sources of genistein induces estrogen-independence in the human breast cancer (MCF-7) xenograft model. Mol Nutr Food Res 59(3): 413-423

Andres S, Abraham K, Appel KE, Lampen A (2011) Risks and benefits of dietary isoflavones for cancer. Crit Rev Toxicol 41(6): 463-506

Appelt LC, Reicks MM (1999) Soy induces phase II enzymes but does not inhibit dimethylbenz[a]anthracene-induced carcinogenesis in female rats. J Nutr 129(10): 1820-1826

Arjmandi BH, Khalil DA, Smith BJ, et al. (2003) Soy protein has a greater effect on bone in postmenopausal women not on hormone replacement therapy, as evidenced by reducing bone resorption and urinary calcium excretion. J Clin Endocrinol Metab 88(3):1048-1054

Atkinson C, Warren RM, Sala E, et al. (2004) Red-clover-derived isoflavones and mammographic breast density: a double-blind, randomized, placebo-controlled trial [ISRCTN42940165]. Breast Cancer Res 6(3): R170-R179

Atteritano M, Mazzaferro S, Frisina A, et al. (2009) Genistein effects on quantitative ultrasound parameters and bone mineral density in osteopenic postmenopausal women. Osteoporos Int 20(11): 1947-1954

Bitto A, Polito F, Atteritano M, et al. (2010) Genistein aglycone does not affect thyroid function: results from a three-year, randomized, double-blind, placebo-controlled trial. J Clin Endocrinol Metab 95(6): 3067-3072

Brown BD, Thomas W, Hutchins A, Martini MC, Slavin JL (2002) Types of dietary fat and soy minimally affect hormones and biomarkers associated with breast cancer risk in premenopausal women. Nutr Cancer 43(1): 22-30

Bruce B, Messina M, Spiller GA (2003) Isoflavone supplements do not affect thyroid function in iodine-replete postmenopausal women. J Med Food 6(4): 309-316

Charles C, Yuskavage J, Carlson O, et al. (2009) Effects of high-dose isoflavones on metabolic and inflammatory markers in healthy postmenopausal women. Menopause 16(2):395-400

Chen M, Rao Y, Zheng Y, et al. (2014) Association between soy isoflavone intake and breast cancer risk for pre- and post-menopausal women: a meta-analysis of epidemiological studies. PloS One 9(2): e89288

Chi F, Wu R, Zeng YC, Xing R, Liu Y, Xu ZG (2013) Post-diagnosis soy food intake and breast cancer survival: a meta-analysis of cohort studies. Asian Pac J Cancer Prev 14(4): 2407-2412

Cohen LA, Zhao Z, Pittman B, Scimeca JA (2000) Effect of intact and isoflavone-depleted soy protein on NMU-induced rat mammary tumorigenesis. Carcinogenesis 21(5): 929-935

D'Anna R, Baviera G, Corrado F, Cancellieri F, Crisafulli A, Squadrito F (2005) The effect of the phytoestrogen genistein and hormone replacement therapy on homocysteine and C-reactive protein level in postmenopausal women. Acta Obst Gynecol Scand 84(5): 474-477

D'Anna R, Cannata ML, Marini H, et al. (2009) Effects of the phytoestrogen genistein on hot flushes, endometrium, and vaginal epithelium in postmenopausal women: a 2-year randomized, double-blind, placebo-controlled study. Menopause 16(2): 301-306

Day JK, Besch-Williford C, McMann TR, Hufford MG, Lubahn DB, MacDonald RS (2001) Dietary genistein increased DMBA-induced mammary adenocarcinoma in wild-type, but not ER alpha KO, mice. Nutr Cancer 39(2): 226-232

de Assis S, Warri A, Benitez C, Helferich W, Hilakivi-Clarke L (2011) Protective effects of prepubertal genistein exposure on mammary tumorigenesis are dependent on BRCA1 expression. Cancer Prev Res 4(9): 1436-1448

Dewell A, Weidner G, Sumner MD, et al. (2007) Relationship of dietary protein and soy isoflavones to serum IGF-1 and IGF binding proteins in the Prostate Cancer Lifestyle Trial. Nutr Cancer 58(1): 35-42

Djuric Z, Chen G, Doerge DR, Heilbrun LK, Kucuk O (2001) Effect of soy isoflavone supplementation on markers of oxidative stress in men and women. Cancer Lett 172(1): 1-6

Dong JY, Qin LQ (2011) Soy isoflavones consumption and risk of breast cancer incidence or recurrence: a meta-analysis of prospective studies. Breast Cancer Res Treat 125(2): 315-323

Duncan AM, Underhill KE, Xu X, Lavalleur J, Phipps WR, Kurzer MS (1999) Modest hormonal effects of soy isoflavones in postmenopausal women. J Clin Endocrinol Metab 84(10):3479-3484

Fritz H, Seely D, Flower G, et al. (2013) Soy, red clover, and isoflavones and breast cancer: a systematic review. PloS One 8(11): e81968

Fritz WA, Coward L, Wang J, Lamartiniere CA (1998) Dietary genistein: perinatal mammary cancer prevention, bioavailability and toxicity testing in the rat. Carcinogenesis 19(12): 2151-2158

Gallo D, Ferlini C, Fabrizi M, Prislei S, Scambia G (2006) Lack of stimulatory activity of a phytoestrogen-containing soy extract on the growth of breast cancer tumors in mice. Carcinogenesis 27(7): 1404-1409

Gallo D, Zannoni GF, Apollonio P, et al. (2005) Characterization of the pharmacologic profile of a standardized soy extract in the ovariectomized rat model of menopause: effects on bone, uterus, and lipid profile. Menopause 12(5): 589-600

Gotoh T, Yamada K, Yin H, Ito A, Kataoka T, Dohi K (1998) Chemoprevention of N-nitroso-N-methylurea-induced rat mammary carcinogenesis by soy foods or biochanin A. Jpn J Cancer Res 89(2): 137-142

Greany KA, Nettleton JA, Wangen KE, Thomas W, Kurzer MS (2008) Consumption of isoflavone-rich soy protein does not alter homocysteine or markers of inflammation in postmenopausal women. Eur J Clin Nutr 62(12): 1419-1425

Hall WL, Vafeiadou K, Hallund J, et al. (2005) Soy-isoflavone-enriched foods and inflammatory biomarkers of cardiovascular disease risk in postmenopausal women: interactions with genotype and equol production. Am J Clin Nutr 82(6): 1260-1268; quiz 1365-1366

Hankinson SE, Willett WC, Colditz GA, et al. (1998) Circulating concentrations of insulin-like growth factor-I and risk of breast cancer. Lancet 351(9113): 1393-1396

Hewitt AL, Singletary KW (2003) Soy extract inhibits mammary adenocarcinoma growth in a syngeneic mouse model. Cancer Lett 192(2): 133-143

Hilakivi-Clarke L, Cho E, Onojafe I, Raygada M, Clarke R (1999a) Maternal exposure to genistein during pregnancy increases carcinogen-induced mammary tumorigenesis in female rat offspring. Oncol Rep 6(5): 1089-1095

Hilakivi-Clarke L, Onojafe I, Raygada M, et al. (1999b) Prepubertal exposure to zearalenone or genistein reduces mammary tumorigenesis. Br J Cancer 80(11): 1682-1688

Hooper L, Madhavan G, Tice JA, Leinster SJ, Cassidy A (2010) Effects of isoflavones on breast density in pre- and post-menopausal women: a systematic review and meta-analysis of randomized controlled trials. Hum Reprod Update 16(6): 745-760

Hooper L, Ryder JJ, Kurzer MS, et al. (2009) Effects of soy protein and isoflavones on circulating hormone concentrations in pre- and post-menopausal women: a systematic review and meta-analysis. Hum Reprod Update 15(4): 423-440

Hwang IK, Lee YB, Yoo KY, et al. (2006) Soybean isoflavones alter parvalbumin in hippocampus of mid-aged normal female, ovariectomized female, and normal male rats. Acta Pharmacol Sin 27(1): 59-65

Jin Z, MacDonald RS (2002) Soy isoflavones increase latency of spontaneous mammary tumors in mice. J Nutr 132(10): 3186-3190

Ju YH, Allred CD, Allred KF, Karko KL, Doerge DR, Helferich WG (2001) Physiological concentrations of dietary genistein dose-dependently stimulate growth of estrogen-dependent human breast cancer (MCF-7) tumors implanted in athymic nude mice. J Nutr 131(11): 2957-2962

Ju YH, Fultz J, Allred KF, Doerge DR, Helferich WG (2006) Effects of dietary daidzein and its metabolite, equol, at physiological concentrations on the growth of estrogen-dependent human breast cancer (MCF-7) tumors implanted in ovariectomized athymic mice. Carcinogenesis 27(4): 856-863

Kataoka M, Atkinson C, Warren R, et al. (2008) Mammographic density using two computer-based methods in an isoflavone trial. Maturitas 59(4): 350-357

Khalil DA, Lucas EA, Juma S, Smith BJ, Payton ME, Arjmandi BH (2002) Soy protein supplementation increases serum insulin-like growth factor-I in young and old men but does not affect markers of bone metabolism. J Nutr 132(9): 2605-2608

Khan SA, Chatterton RT, Michel N, et al. (2012) Soy isoflavone supplementation for breast cancer risk reduction: a randomized phase II trial. Cancer Prev Res 5(2): 309-319

Khaodhiar L, Ricciotti HA, Li L, et al. (2008) Daidzein-rich isoflavone aglycones are potentially effective in reducing hot flashes in menopausal women. Menopause 15(1): 125-132

Kijkuokool P, Parhar IS, Malaivijitnond S (2006) Genistein enhances *N*-nitrosomethylurea-induced rat mammary tumorigenesis. Cancer Lett 242(1): 53-59

Kinouchi FL, Maia DC, de Abreu Ribeiro LC, et al. (2012) A soy-based product fermented by *Enterococcus faecium* and *Lactobacillus helveticus* inhibits the development of murine breast adenocarcinoma. Food Chem Toxicol 50(11): 4144-4148

Ko KP (2014) Isoflavones: Chemistry, analysis, functions and effects on health and cancer. Asian Pac J Cancer Prev 15(17): 7001-7010

Kok L, Kreijkamp-Kaspers S, Grobbee DE, Lampe JW, van der Schouw YT (2005a) A randomized, placebo-controlled trial on the effects of soy protein containing isoflavones on quality of life in postmenopausal women. Menopause 12(1): 56-62

Kok L, Kreijkamp-Kaspers S, Grobbee DE, Lampe JW, van der Schouw YT (2005b) Soy isoflavones, body composition, and physical performance. Maturitas 52(2): 102-110

Kok L, Kreijkamp-Kaspers S, Grobbee DE, van der Schouw YT (2004) Design and baseline characteristics of a trial on health effects of soy protein with isoflavones in postmenopausal women. Maturitas 47(1): 21-29

Kreijkamp-Kaspers S, Kok L, Bots ML, Grobbee DE, Lampe JW, van der Schouw YT (2005) Randomized controlled trial of the effects of soy protein containing isoflavones on vascular function in postmenopausal women. Am J Clin Nutr 81(1): 189-195

Kreijkamp-Kaspers S, Kok L, Grobbee DE, et al. (2004) Effect of soy protein containing isoflavones on cognitive function, bone mineral density, and plasma lipids in postmenopausal women: a randomized controlled trial. JAMA 292(1): 65-74

Kuiper GG, Lemmen JG, Carlsson B, et al. (1998) Interaction of estrogenic chemicals and phytoestrogens with estrogen receptor β. Endocrinology 139(10): 4252-4263

Lamartiniere CA, Cotroneo MS, Fritz WA, Wang J, Mentor-Marcel R, Elgavish A (2002) Genistein chemoprevention: timing and mechanisms of action in murine mammary and prostate. J Nutr 132(3): 552S-558S

Lamartiniere CA, Moore JB, Brown NM, Thompson R, Hardin MJ, Barnes S (1995) Genistein suppresses mammary cancer in rats. Carcinogenesis 16(11): 2833-2840

Levis S, Strickman-Stein N, Ganjei-Azar P, Xu P, Doerge DR, Krischer J (2011) Soy isoflavones in the prevention of menopausal bone loss and menopausal symptoms: a randomized, double-blind trial. Arch Int Med 171(15): 1363-1369

Liu B, Edgerton S, Yang X, et al. (2005) Low-dose dietary phytoestrogen abrogates tamoxifen-associated mammary tumor prevention. Cancer Res 65(3): 879-886

Liu X, Suzuki N, Santosh Laxmi YR, Okamoto Y, Shibutani S (2012) Anti-breast cancer potential of daidzein in rodents. Life Sci 91(11-12): 415-419

Liu Y, Hilakivi-Clarke L, Zhang Y, et al. (2015) Isoflavones in soy flour diet have different effects on whole-genome expression patterns than purified isoflavone mix in human MCF-7 breast tumors in ovariectomized athymic nude mice. Mol Nutr Food Res 59(8): 1419-1430

Lu LJ, Cree M, Josyula S, Nagamani M, Grady JJ, Anderson KE (2000) Increased urinary excretion of 2-hydroxyestrone but not 16α-hydroxyestrone in premenopausal women during a soya diet containing isoflavones. Cancer Res 60(5): 1299-1305

Ma D, Zhang Y, Yang T, Xue Y, Wang P (2014) Isoflavone intake inhibits the development of 7,12-dimethylbenz(a)anthracene(DMBA)-induced mammary tumors in normal and ovariectomized rats. J Clin Biochem Nutr 54(1): 31-38

Marini H, Bitto A, Altavilla D, et al. (2008) Breast safety and efficacy of genistein aglycone for postmenopausal bone loss: a follow-up study. J Clin Endocrinol Metab 93(12): 4787-4796

Marini H, Minutoli L, Polito F, et al. (2007) Effects of the phytoestrogen genistein on bone metabolism in osteopenic postmenopausal women: a randomized trial. Ann Intern Med 146(12): 839-847

Marnett LJ (2000) Oxyradicals and DNA damage. Carcinogenesis 21(3): 361-370

Martinez-Montemayor MM, Otero-Franqui E, Martinez J, De La Mota-Peynado A, Cubano LA, Dharmawardhane S (2010) Individual and combined soy isoflavones exert differential effects on metastatic cancer progression. Clin Exp Metastasis 27(7): 465-480

Martini MC, Dancisak BB, Haggans CJ, Thomas W, Slavin JL (1999) Effects of soy intake on sex hormone metabolism in premenopausal women. Nutr Cancer 34(2):133-139

Maskarinec G, Franke AA, Williams AE, et al. (2004a) Effects of a 2-year randomized soy intervention on sex hormone levels in premenopausal women. Cancer Epidemiol Biomarkers Prev 13(11 Pt 1): 1736-1744

Maskarinec G, Hebshi S, Custer L, Franke AA (2008) The relation of soy intake and isoflavone levels in nipple aspirate fluid. Eur J Cancer Prev 17(1): 67-70

Maskarinec G, Morimoto Y, Conroy SM, Pagano IS, Franke AA (2011a) The volume of nipple aspirate fluid is not affected by 6 months of treatment with soy foods in premenopausal women. J Nutr 141(4): 626-630

Maskarinec G, Morimoto Y, Heak S, et al. (2012) Urinary estrogen metabolites in two soy trials with premenopausal women. Eur J Clin Nutr 66(9): 1044-1049

Maskarinec G, Ollberding NJ, Conroy SM, et al. (2011b) Estrogen levels in nipple aspirate fluid and serum during a randomized soy trial. Cancer Epidemiol Biomarkers Prev 20(9): 1815-1821

Maskarinec G, Steude JS, Franke AA, Cooney RV (2009a) Inflammatory markers in a 2-year soy intervention among premenopausal women. J Inflamm 6: 9

Maskarinec G, Suzuki S, Pagano IS, Morimoto Y, Franke AA, Ehya H (2013) Cytology in nipple aspirate fluid during a randomized soy food intervention among premenopausal women. Nutr Cancer 65(8): 1116-1121

Maskarinec G, Takata Y, Franke AA, Williams AE, Murphy SP (2004b) A 2-year soy intervention in premenopausal women does not change mammographic densities. J Nutr 134(11): 3089-3094

Maskarinec G, Takata Y, Murphy SP, Franke AA, Kaaks R (2005) Insulin-like growth factor-1 and binding protein-3 in a 2-year soya intervention among premenopausal women. Br J Nutr 94(3): 362-367

Maskarinec G, Verheus M, Steinberg FM, et al. (2009b) Various doses of soy isoflavones do not modify mammographic density in postmenopausal women. J Nutr 139(5): 981-986

Maskarinec G, Williams AE, Carlin L (2003) Mammographic densities in a one-year isoflavone intervention. Eur J Cancer Prev 12(2): 165-169

Maskarinec G, Williams AE, Inouye JS, Stanczyk FZ, Franke AA (2002) A randomized isoflavone intervention among premenopausal women. Cancer Epidemiol Biomarkers Prev 11(2): 195-201

McLaughlin JM, Olivo-Marston S, Vitolins MZ, et al. (2011) Effects of tomato- and soy-rich diets on the IGF-I hormonal network: a crossover study of postmenopausal women at high risk for breast cancer. Cancer Prev Res 4(5): 702-710

Mittal N, Hota D, Dutta P, et al. (2011) Evaluation of effect of isoflavone on thyroid economy & autoimmunity in oophorectomised women: a randomised, double-blind, placebo-controlled trial. Indian J Med Res 133: 633-640

Möller FJ, Pemp D, Soukup ST, et al. (2016) Soy isoflavone exposure through all life stages accelerates 17beta-estradiol-induced mammary tumor onset and growth, yet reduces tumor burden, in ACI rats. Arch Toxicol 90(8): 1907-1916

Morimoto Y, Conroy SM, Pagano IS, et al. (2012) Urinary estrogen metabolites during a randomized soy trial. Nutr Cancer 64(2): 307-314

Nasca MM, Zhou JR, Welty FK (2008) Effect of soy nuts on adhesion molecules and markers of inflammation in hypertensive and normotensive postmenopausal women. Am J Cardiol 102(1): 84-86

Nettleton JA, Greany KA, Thomas W, Wangen KE, Adlercreutz H, Kurzer MS (2005) The effect of soy consumption on the urinary 2:16-hydroxyestrone ratio in postmenopausal women depends on equol production status but is not influenced by probiotic consumption. J Nutr 135(3): 603-608

Nhan S, Anderson KE, Nagamani M, Grady JJ, Lu LJ (2005) Effect of a soymilk supplement containing isoflavones on urinary F2 isoprostane levels in premenopausal women. Nutr Cancer 53(1): 73-81

Nikander E, Metsa-Heikkila M, Tiitinen A, Ylikorkala O (2003) Evidence of a lack of effect of a phytoestrogen regimen on the levels of C-reactive protein, E-selectin, and nitrate in postmenopausal women. J Clin Endocrinol Metab 88(11): 5180-5185

Ono M, Koga T, Ueo H, Nakano S (2012) Effects of dietary genistein on hormone-dependent rat mammary carcinogenesis induced by ethyl methanesulphonate. Nutr Cancer 64(8): 1204-1210

Onoda A, Ueno T, Uchiyama S, Hayashi S, Kato K, Wake N (2011) Effects of S-equol and natural S-equol supplement (SE5-OH) on the growth of MCF-7 *in vitro* and as tumors implanted into ovariectomized athymic mice. Food Chem Toxicol 49(9): 2279-2284

Persky VW, Turyk ME, Wang L, et al. (2002) Effect of soy protein on endogenous hormones in postmenopausal women. Amer J Clin Nutr 75(1): 145-153

Pervin S, Chaudhuri G, Singh R (2010) NO to breast: when, why and why not? Current Pharm Des 16(4): 451-462

Petrakis NL (1993) Nipple aspirate fluid in epidemiologic studies of breast disease. Epidemiol Rev 15(1): 188-195

Petrakis NL, Barnes S, King EB, et al. (1996) Stimulatory influence of soy protein isolate on breast secretion in pre- and postmenopausal women. Cancer Epidemiol Biomarkers Prev 5(10): 785-794

Pop EA, Fischer LM, Coan AD, Gitzinger M, Nakamura J, Zeisel SH (2008) Effects of a high daily dose of soy isoflavones on DNA damage, apoptosis, and estrogenic outcomes in healthy postmenopausal women: a phase I clinical trial. Menopause 15(4 Pt 1): 684-692

Power KA, Chen JM, Saarinen NM, Thompson LU (2008) Changes in biomarkers of estrogen receptor and growth factor signaling pathways in MCF-7 tumors after short- and long-term treatment with soy and flaxseed. J Steroid Biochem Mol Biol 112(1-3): 13-19

Power KA, Saarinen NM, Chen JM, Thompson LU (2006) Mammalian lignans enterolactone and enterodiol, alone and in combination with the isoflavone genistein, do not promote the growth of MCF-7 xenografts in ovariectomized athymic nude mice. Int J Cancer 118(5):1316-1320

Powles TJ, Howell A, Evans DG, et al. (2008) Red clover isoflavones are safe and well tolerated in women with a family history of breast cancer. Menopause Int 14(1): 6-12

Pudenz M, Roth K, Gerhauser C (2014) Impact of soy isoflavones on the epigenome in cancer prevention. Nutrients 6(10): 4218-4272

Qin LQ, Xu JY, Wang PY, Hoshi K (2006) Soyfood intake in the prevention of breast cancer risk in women: a meta-analysis of observational epidemiological studies. J Nutr Sci Vitaminol 52(6): 428-436

Qin W, Zhu W, Shi H, et al. (2009) Soy isoflavones have an antiestrogenic effect and alter mammary promoter hypermethylation in healthy premenopausal women. Nutr Cancer 61(2): 238-244

Renehan AG, Zwahlen M, Minder C, O'Dwyer ST, Shalet SM, Egger M (2004) Insulin-like growth factor (IGF)-I, IGF binding protein-3, and cancer risk: systematic review and meta-regression analysis. Lancet 363(9418): 1346-1353

Roberts RA, Laskin DL, Smith CV, et al. (2009) Nitrative and oxidative stress in toxicology and disease. Toxicol Sci 112(1): 4-16

Ryan-Borchers T, Chew B, Park JS, McGuire M, Fournier L, Beerman K (2008) Effects of dietary and supplemental forms of isoflavones on thyroid function in healthy postmenopausal women. Topics Clin Nutr 23(1): 13-22

Ryan-Borchers TA, Park JS, Chew BP, McGuire MK, Fournier LR, Beerman KA (2006) Soy isoflavones modulate immune function in healthy postmenopausal women. Am J Clin Nutr 83(5): 1118-1125

Saarinen NM, Power K, Chen J, Thompson LU (2006) Flaxseed attenuates the tumor growth stimulating effect of soy protein in ovariectomized athymic mice with MCF-7 human breast cancer xenografts. Int J Cancer 119(4): 925-931

Sathyapalan T, Aye M, Rigby AS, et al. (2017) Soy reduces bone turnover markers in women during early menopause: A randomized controlled trial. J Bone Miner Res 32(1): 157-164

Sen C, Morimoto Y, Heak S, Cooney RV, Franke AA, Maskarinec G (2012) Soy foods and urinary isoprostanes: results from a randomized study in premenopausal women. Food Funct 3(5): 517-521

Setchell KD, Cassidy A (1999) Dietary isoflavones: biological effects and relevance to human health. J Nutr 129(3): 758S-767S

Shao ZM, Wu J, Shen ZZ, Barsky SH (1998) Genistein exerts multiple suppressive effects on human breast carcinoma cells. Cancer Res 58(21): 4851-4857

Simmen RC, Eason RR, Till SR, et al. (2005) Inhibition of NMU-induced mammary tumorigenesis by dietary soy. Cancer Lett 224(1): 45-52

Steinberg FM, Murray MJ, Lewis RD, et al. (2011) Clinical outcomes of a 2-y soy isoflavone supplementation in menopausal women. Am J Clin Nutr 93(2): 356-367

Su Y, Eason RR, Geng Y, Till SR, Badger TM, Simmen RC (2007) In utero exposure to maternal diets containing soy protein isolate, but not genistein alone, protects young adult rat offspring from NMU-induced mammary tumorigenesis. Carcinogenesis 28(5): 1046-1051

Tanaka Y, Wood LA, Cooney RV (2007) Enhancement of intracellular gamma-tocopherol levels in cytokine-stimulated C3H 10T1/2 fibroblasts: relation to NO synthesis, isoprostane formation, and tocopherol oxidation. BMC Chemical Biol 7: 2

Tice JA (2005) Soy and tamoxifen for breast cancer prevention in high risk pre-menopausal women. Final report for the US Army Medical Research and Material Command. <http://wwwdticmil/dtic/tr/fulltext/u2/a460295pdf> (accessed 15/02/2018)

Tice JA, Guthrie N, Shepherd J, Kerlikowske K, Esserman L (2005 ) Soy for the prevention of breast cancer - a randomized trial. MD, 8-11 June 2005, Era of Hope Meeting Baltimore (cited from Hooper et al., 2010)

Toniolo P, Bruning PF, Akhmedkhanov A, et al. (2000) Serum insulin-like growth factor-I and breast cancer. Int J Cancer 88(5): 828-832

Tormala R, Appt S, Clarkson TB, et al. (2008) Impact of soy supplementation on sex steroids and vascular inflammation markers in postmenopausal women using tibolone: role of equol production capability. Climacteric 11(5): 409-415

Trock BJ, Hilakivi-Clarke L, Clarke R (2006) Meta-analysis of soy intake and breast cancer risk. J Natl Cancer Inst 98(7): 459-471

Verheus M, van Gils CH, Kreijkamp-Kaspers S, et al. (2008) Soy protein containing isoflavones and mammographic density in a randomized controlled trial in postmenopausal women. Cancer Epidemiol Biomarkers Prev 17(10): 2632-2638

Welcsh PL, King MC (2001) BRCA1 and BRCA2 and the genetics of breast and ovarian cancer. Hum Mol Genet 10(7): 705-713

Wiseman H, O'Reilly JD, Adlercreutz H, et al. (2000) Isoflavone phytoestrogens consumed in soy decrease F(2)-isoprostane concentrations and increase resistance of low-density lipoprotein to oxidation in humans. Am J Clin Nutr 72(2): 395-400

Woodside JV, Campbell MJ, Denholm EE, et al. (2006) Short-term phytoestrogen supplementation alters insulin-like growth factor profile but not lipid or antioxidant status. J Nutr Biochem 17(3): 211-215

Wu AH, Yu MC, Tseng CC, Pike MC (2008) Epidemiology of soy exposures and breast cancer risk. Br J Cancer 98(1): 9-14

Wu YC, Zheng D, Sun JJ, Zou ZK, Ma ZL (2015) Meta-analysis of studies on breast cancer risk and diet in Chinese women. Int J Clin Exp Med 8(1): 73-85

Xie Q, Chen ML, Qin Y, et al. (2013) Isoflavone consumption and risk of breast cancer: a dose-response meta-analysis of observational studies. Asia Pac J Clin Nutr 22(1): 118-127

Xu X, Duncan AM, Merz BE, Kurzer MS (1998) Effects of soy isoflavones on estrogen and phytoestrogen metabolism in premenopausal women. Cancer Epidemiol Biomarkers Prev 7(12): 1101-1108

Xu X, Duncan AM, Wangen KE, Kurzer MS (2000) Soy consumption alters endogenous estrogen metabolism in postmenopausal women. Cancer Epidemiol Biomarkers Prev 9(8): 781-786

Yager JD, Liehr JG (1996) Molecular mechanisms of estrogen carcinogenesis. Ann Rev Pharmacol Toxicol 36: 203-232

Yang X, Edgerton SM, Kosanke SD, et al. (2003) Hormonal and dietary modulation of mammary carcinogenesis in mouse mammary tumor virus-c-erbB-2 transgenic mice. Cancer Res 63(10): 2425-2433

Yildiz MF, Kumru S, Godekmerdan A, Kutlu S (2005) Effects of raloxifene, hormone therapy, and soy isoflavone on serum high-sensitive C-reactive protein in postmenopausal women. Int J Gynaecol Obstet 90(2): 128-133

Yuan L, Wagatsuma C, Yoshida M, et al. (2003) Inhibition of human breast cancer growth by GCP (genistein combined polysaccharide) in xenogeneic athymic mice: involvement of genistein biotransformation by β-glucuronidase from tumor tissues. Mutat Res 523-524: 55-62

Zhou JR, Yu L, Mai Z, Blackburn GL (2004) Combined inhibition of estrogen-dependent human breast carcinoma by soy and tea bioactive components in mice. Int J Cancer 108(1): 8-14

Zhu BT, Conney AH (1998) Functional role of estrogen metabolism in target cells: review and perspectives. Carcinogenesis 19(1): 1-27
